# First Total Syntheses and Antimicrobial Evaluation of Penicimonoterpene, a Marine-Derived Monoterpenoid, and Its Various Derivatives

**DOI:** 10.3390/md12063352

**Published:** 2014-06-03

**Authors:** Jian-Chun Zhao, Xiao-Ming Li, James B. Gloer, Bin-Gui Wang

**Affiliations:** 1 Key Laboratory of Experimental Marine Biology, Institute of Oceanology, Chinese Academy of Sciences, Nanhai Road 7, Qingdao 266071, China; E-Mails: jianchun017@163.com (J.-C.Z.); lixmqd@aliyun.com (X.-M.L.); 2 University of Chinese Academy of Sciences, Yuquan Road 19A, Beijing 100049, China; 3 Department of Chemistry, University of Iowa, Iowa City, IA 52242, USA; E-Mail: james-gloer@uiowa.edu

**Keywords:** (±)-penicimonoterpene, total synthesis, antimicrobial activity, structure-activity relationship

## Abstract

The first total synthesis of marine-derived penicimonoterpene (±)-**1** has been achieved in four steps from 6-methylhept-5-en-2-one using a Reformatsky reaction as the key step to construct the basic carbon skeleton. A total of 24 new derivatives of **1** have also been designed and synthesized. Their structures were characterized by analysis of their ^1^H NMR, ^13^C NMR and HRESIMS data. Some of them showed significant antibacterial activity against *Aeromonas hydrophila*, *Escherichia coli*, *Micrococcus luteus*, *Staphylococcus aureus*, *Vibrio anguillarum*, *V*. *harveyi* and/or *V*. *parahaemolyticus*, and some showed activity against plant-pathogenic fungi (*Alternaria brassicae*, *Colletotrichum gloeosporioides* and/or *Fusarium graminearum*). Some of the derivatives exhibited antimicrobial MIC values ranging from 0.25 to 4 μg/mL, which were stronger than those of the positive control. Notably, Compounds **3b** and **10** showed extremely high selectively against plant-pathogenic fungus *F. graminearum* (MIC 0.25 μg/mL) and pathogenic bacteria *E. coli* (MIC 1 μg/mL), implying their potential as antimicrobial agents. SAR analysis of **1** and its derivatives indicated that modification of the carbon-carbon double bond at C-6/7, of groups on the allylic methylene unit and of the carbonyl group at C-1, effectively enhanced the antimicrobial activity.

## 1. Introduction

In recent years, there has been increasing interest in research on marine natural products, since an enormous range of chemically diverse biologically active metabolites have been discovered from marine organisms [1,2,3,4]. Recently, marine-derived fungi have triggered our interest, due to their ability to produce structurally interesting bioactive secondary metabolites [5,6,7,8,9].

**Figure 1:**
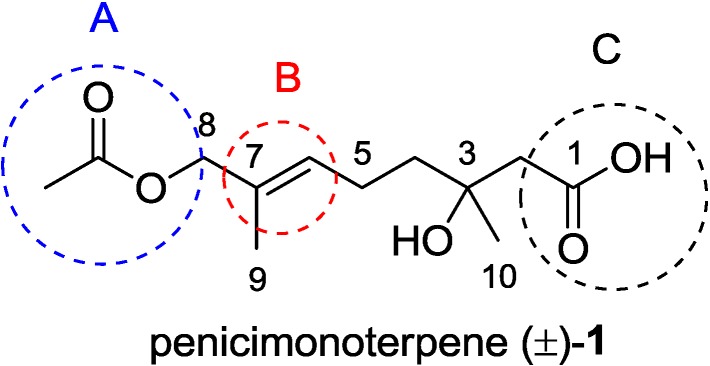
Structure of penicimonoterpene (±)-**1**.

Terpenoids comprise an important class of bioactive agents. Despite their relatively simple structures, some of these compounds exhibit interesting biological activities [10,11,12,13,14]. We have recently reported the isolation and identification of a new monoterpenoid, penicimonoterpene (+)-**1** (Figure 1), from a marine-derived endophytic isolate of the fungus, *Penicillium chrysogenum* QEN-24S [15]. This compound exhibited potent activity against the plant pathogen, *Alternaria brassicae*, comparable to that of a positive control [15].

Herein, we report a short synthesis of (±)-**1** featuring a Reformatsky reaction as a key step. This approach has the merits of low cost, mild reaction conditions and easy access to diversity-oriented derivatives for potential structure-activity relationship (SAR) investigation. In antimicrobial assays, penicimonoterpene (±)-**1** not only displayed antifungal activity against *A. brassicae*, but also showed potent antibacterial activity against marine bacteria, including *Aeromonas hydrophila*, *Vibrio harveyi*, and *V. parahaemolyticus*. Therefore, we became interested in designing and synthesizing diverse antibacterial inhibitors using (±)-**1** as a model compound. Modifications were focused on variation of the substituents at the eight-position (Section A in Figure 1), the carbon-carbon double bond at C-6/7 (Section B) and carboxyl substituents at C-1 (Section C).

## 2. Results and Discussion

### 2.1. Chemistry

#### 2.1.1. Synthesis of Penicimonoterpene (±)-**1**

**Figure 2:**
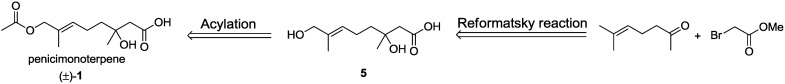
Retrosynthetic analysis for penicimonoterpene (±)-**1**.

We report herein a practical and efficient racemic synthesis of **1** from 6-methylhept-5-en-2-one using a Reformatsky reaction as the key step. Retrosynthetic analysis (Figure 2) led to **5** as the key target intermediate, which could be accessible via three routes, as listed in Scheme I.

**Scheme I:**
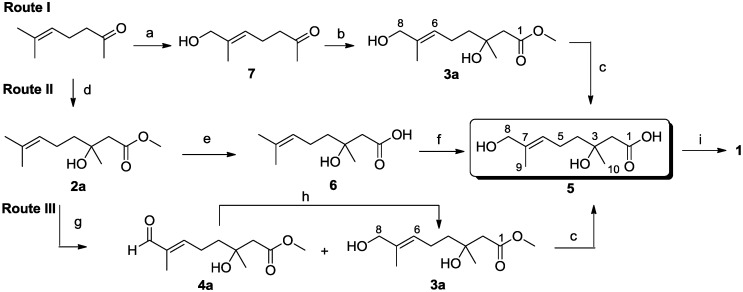
Three routes for the total synthesis of penicimonoterpene (±)-**1**. Reagents and conditions: (**a**) SeO_2_ (0.1 eq), *t*-BuOOH (3.0 eq), CH_2_Cl_2_, room temperature (rt.), 19 h, 38%; (**b**) BrCH_2_COOCH_3_ (2.0 eq), Zn (2.0 eq), THF, reflux, 2 h, 4%; (**c**) KOH (2.2 eq), MeOH: H_2_O (*v*/*v*, 3:1), reflux 5 h, 85%; (**d**) BrCH_2_COOCH_3_ (2.0 eq), Zn (2.0 eq), THF, reflux, 2 h, 85%; (**e**) KOH (2.2 eq), MeOH: H_2_O (*v*/*v*, 3:1), reflux 5 h, 94%; (**f**) SeO_2_ (0.1 eq), *t*-BuOOH (3.0 eq), CH_2_Cl_2_, rt., 19 h, 11%; (**g**) SeO_2_ (0.1 eq), *t*-BuOOH (3.0 eq), CH_2_Cl_2_, rt., 19 h, 25% for **4a**, 29% for **3a**; (**h**) NaBH_4_ (1.0 eq), MeOH, rt., 1 h, 69%; (**i**) Ac_2_O (1.5 eq), 4-dimethylaminopyridine (DMAP) catalyst (cat.), Et_3_N (1.5 eq), CH_2_Cl_2_, rt., overnight, 55%.

In Route I, selective oxidation of 6-methylhept-5-en-2-one with SeO_2_ in CH_2_Cl_2_ [16,17,18] yielded allylic alcohol **7**, which was converted to methyl ester **3a** by a Reformatsky reaction using 2.0 eq of BrCH_2_COOCH_3_ in the presence of 2.0 eq of Zn powder in refluxing THF. Methyl ester **3a** was then hydrolyzed with KOH to afford key intermediate **5**. However, this route was not considered satisfactory, because the yield of the Reformatsky reaction step was only 4%.

In Route II, the Reformatsky reaction of 6-methylhept-5-en-2-one with BrCH_2_COOCH_3_ yielded **2a** in a good yield (85%). After refluxing with 2.2 eq of KOH in a mixture of MeOHH_2_O (*v*/*v*, 3/1), Compound **2a** was hydrolyzed in excellent yield (94%) to produce Compound **6**, which was subsequently oxidized to give **5** with 11% yield. Unfortunately, Compound **5** proved to be difficult to purify from by-products formed in the reaction, leading to the poor yield obtained for this step. In Route III, treatment of Compound **2a** with 3.0 eq of *t*-BuOOH in the presence of 0.1 eq of SeO_2_ afforded a chromatographically separable mixture of aldehyde **4a** (25%) and alcohol **3a** (29%). The former could be converted into the latter (69%) by reduction with NaBH_4_ in MeOH. In an effort to improve the efficiency of this process, we studied the conditions for the selective oxidation of the allylic methyl groups of **2a**. A microwave irradiation method using SeO_2_ and *t*-BuOOH adsorbed on SiO_2_ [19] also afforded **4a**, but in low yield (13%). After optimization by extensive variation of the number of equivalents of SeO_2_ and the reaction time, it was found that the best results were obtained with 0.1 eq of SeO_2_, and 3.0 eq *t*-BuOOH in CH_2_Cl_2_, reacting for 19 h at room temperature, yielded Compound **3a** in 29% yield and Compound **4a** in 25% yield. The key intermediate, **5**, was obtained in 85% yield by saponification of **3a** with no protection of the tertiary hydroxyl group. Treatment of intermediate **5** with 1.5 eq of Ac_2_O, a catalytic amount of 4-dimethylaminopyridine (DMAP) and 1.5 eq of Et_3_N at room temperature for 12 h gave **1** in 55% yield.

As expected, based on the selectivity rules and site for the SeO_2_-mediated oxidation reaction [20], as well as the mechanism of this process [21], the Δ^6,7^-double bond in alcohol **3a** was found to have the *E*-configuration on the basis of a NOESY correlation between H-6 (δ 5.39) and H_2_-8 (δ 3.98). The ^1^H NMR, ^13^C NMR, MS and HRMS data for synthetic (±)-**1** were identical to those of reported natural penicimonoterpene [15].

#### 2.1.2. Synthesis of Diverse Derivatives of (±)-**1**

In order to investigate the influence of structural changes on the antimicrobial activities of **1**, a series of derivatives of **1** were synthesized by modification of three main structural features of **1**, including the 8-acetoxy group, the C-6/7 double bond and the carboxyl group at C-1. The syntheses of these derivatives are summarized in Scheme II and Table 1.

**Scheme II:**
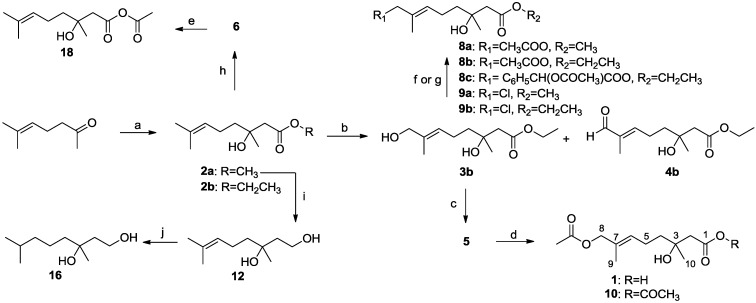
Synthetic routes for diverse derivatives of penicimonoterpene (±)-**1** (**2**–**10**, **12**, **16** and **18**). Reagents and conditions: (**a**) BrCH_2_COOCH_3_ or BrCH_2_COOCH_2_CH_3_ (2.0 eq), Zn (2.0 eq), THF, reflux, 2 h, 85% for **2a**, 95% for **2b**; (**b**) SeO_2_ (0.1 eq), *t*-BuOOH (3.0 eq), CH_2_Cl_2_, rt., 19 h, 81% for **3b** and 16% for **4b**; (**c**) KOH (2.2 eq), MeOH: H_2_O (*v*/*v*, 3:1), reflux 5 h, 91%; (**d**) Ac_2_O (1.5 eq), DMAP (cat.), Et_3_N (1.5 eq), CH_2_Cl_2_, rt., 12 h, 55% for **1**, 18% for **10**; (**e**) Ac_2_O (2.0 eq), DMAP (cat.), Et_3_N (2.0 eq), CH_2_Cl_2_, rt., 24 h, 85%; (**f**) Ac_2_O (1.5 eq), DMAP (cat.), Et_3_N (1.5 eq), CH_2_Cl_2_, rt., 12 to 18 h, 91%–95% for **8a** and **8b**; *R*-(−)-*O*-acyl mandelic acid (1.1 eq), *N,N*′-dicyclohexylcarbodiimide (DCC, 1.1 eq), DMAP (0.1 eq) CH_2_Cl_2_, rt., 16 h, 81% for **8c**; (**g**) PPh_3_ (1.5 eq), NCS (*N*-chlorosuccinimide) (1.5 eq), dry CH_2_Cl_2_, 0 °C, overnight, 69%–77% for **9a** and **9b**; (**h**) KOH (2.2 eq), MeOH: H_2_O (*v*/*v*, 3:1), reflux 5 h, 94%; (**i**) LiAlH_4_ (3.0 eq), THF, 0 °C for 2 h, then 65 °C for 12 h, 97% from **2a**; (**j**) Pd/C, H_2_, EtOAc, rt., 12 h, 89%.

**Table 1:** Hydrogenation of diverse derivatives of penicimonoterpene (±)-**1** to afford **11**, **13**–**15**, **17**, **19** and **20**.

Entry	Substrate	Product	Yield (%)
1	**2a**	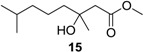	93
2	**8a**		76
3	**9a**		25
4	**2b**	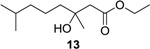	90
5	**8b**		88
6	**8c**		96
7	**9b**		15
8	**10**	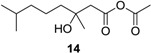	93
9	**18**		89
10	**1**	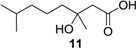	92
11	**6**		67
12	**5**	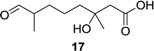	45
13	**3a**	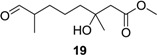	27
14	**3b**	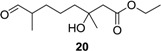	25

Reagents and conditions: Pd/C, H_2_, EtOAc, rt., 12–36 h, 15%–96%.

For the synthesis of these derivatives, BrCH_2_COOCH_2_CH_3_ was used to react with 6-methylhept-5-en-2-one in refluxing THF in the presence of Zn powder to generate **2b**, which was then oxidized with SeO_2_ and *t*-BuOOH to yield Compounds **3b** (81% yield) and **4b** (16% yield) (Scheme II). Saponification of **3a** or **3b** in the presence of KOH, then adjusting the pH to 2~3, yielded Compound **5** in 85%–91% yield with no detectable formation of a cyclic by-product [22]. The use of LiOH for this process gave a very poor yield (11%). Acetylation of **5** was attempted by treatment with 1.5 eq of Ac_2_O in the presence of a catalytic amount of 4-dimethylaminopyridine (DMAP) and 1.5 eq of Et_3_N in CH_2_Cl_2_ at room temperature for 12 h. The major product was *rac*-penicimonoterpene **1** (55% yield), with anhydride product **10** forming in 18% yield. Using the same acetylation method, anhydride **18** was obtained in 85% yield from **6**. Upon treatment of **2a** with 3.0 eq of LiAlH_4_ in anhydrous THF at 0 °C for 2 h, then raising the temperature to 65 °C for 12 h, reduction product **12** was obtained in 97% yield. Treatment of **12** with H_2_ in the presence of 10% Pd/C gave **16** in 89% yield.

In an attempt to enhance the antibacterial activities, we envisaged modification at C-8 by introducing electron-withdrawing groups, such as Cl and Br (Scheme II). Chlorine-substituted products **9a** and **9b** were successfully prepared in 69%–77% yield by using 1.5 eq of NCS (*N*-chlorosuccinimide) as the chlorinating reagent in the presence of PPh_3_ at 0 °C overnight. Unfortunately, NBS (*N*-bromosuccinimide) treatment failed to give the targeted bromination products, possibly because NBS had a tendency to be more reactive than NCS, leading to the bromine-substituted products being less stable, or because other products were formed.

Hydrogenation of **1**, **2a**–**b**, **3a**–**b**, **5**, **6**, **8a**–**c**, **9a**–**b**, **10** and **18** proceeded with H_2_ using 10% Pd/C as the catalyst to give corresponding products, as shown in Table 1. The Pd-catalyzed isomerization of primary allylic alcohols [23], **3a**, **3b**, and **5**, into the corresponding saturated aldehydes have been achieved at room temperature. Another interesting result was that allylic ester groups (e.g., in **8b** and **8c**) were easily removed by catalytic hydrogenation in addition to the reduction of the olefin unit at room temperature with excellent yields. It is worth noting that ester substrates **1**, **8a**–**c** and **10**, prepared by acylation of the corresponding alcohols, as well as chlorine-substituted products **9a** and **9b** were transformed into related methyl products **11**, **13**, **14** and **15** by hydrogenolysis using 10% Pd/C as the catalyst with good yields (76%–96%), while subjecting the parent allylic alcohols, **3a**, **3b** and **5**, to the same conditions did not lead to the corresponding methyl products. These results indicated that chloro, as well as ester groups, might be as valuable as protecting groups, since they could be easily removed by catalytic hydrogenation in high yields.

In summary, we have efficiently synthesized penicimonoterpene (±)-**1** for the first time, as well as 24 racemic derivatives via simple routes and at low cost. Of 24 derivatives, Compounds **3a**–**b**, **4a**–**b**, **8a**–**c**, **9a**–**b**, **10**, **14** and **17**–**20** were new, and these products were fully characterized by NMR and HRESIMS (see Supplementary Information).

### 2.2. Antibacterial Activity and SAR Analysis

Synthetic Compounds **1**–**20** were tested *in vitro* for antibacterial activity against two Gram-positive bacteria (*Micrococcus luteus* and *Staphylococcus aureus*) and five Gram-negative bacteria, including *Escherichia coli* and the marine bacteria, *Aeromonas hydrophila*, *Vibrio anguillarum*, *V. harveyi* and *V. parahaemolyticus*. Many of these derivatives exhibited antibacterial effects. Compounds **13**–**17** showed significant activity, with MIC values ranging from 0.25 to 64 μg/mL, as listed in Table 2. Several observations were made regarding structure-activity relationships among these compounds. Reduction of the carbon-carbon double bond at C-6/7, significantly increasing the antibacterial activity (e.g., **13** *vs*. **2b**, **14** *vs*. **18**, **15** *vs*. **2a**, **16** *vs*. **12**, except for Compounds **19** and **20**, which contain an aldehyde group). For compounds containing the double bond at C-6/7, the general order of antibacterial potency for substituents at C-1 was COOH > COOCH_2_CH_3_ > COOCH_3_ > COOCOCH_3_ (except in the case of *E*. *coli*, for which Compound **10** showed better selective activity). Hydroxylation at C-8 (as in **3a** and **3b**) resulted in loss of activity.

Notably, hydrogenated Compounds **13**–**17** and aldehyde **4b** showed inhibitory activity to all tested bacterial strains, and in many cases, the activity was stronger than or comparable to the corresponding positive controls (Table 2). For *V. parahaemolyticus*, Compounds **9b** and **12** showed activity similar to that of the positive control (the common antibacterial agent, chloramphenicol). Compound **10** exhibited better selective activity against *E*. *coli* with a MIC value of 1 μg/mL, which is 16-fold more potent than that of **1** and two-fold more active than chloramphenicol. Compound **11** exhibited an MIC value of 4 μg/mL against *S*. *aureus*, making it more potent than chloramphenicol in the assay (MIC = 8 μg/mL).

**Table 2:** Minimum inhibitory concentration (MIC, μg/mL) of **1** and its derivatives against seven bacterial strains ^a,b^.

Compounds	A. h.	V. a.	V. h.	V. p.	E. c.	M. l.	S. a.
**1**	64	>64	16	64	16	64	8
**2a**	>64	>64	>64	>64	>64	>64	>64
**2b**	32	64	>64	>64	>64	64	>64
**3a**	>64	>64	>64	>64	>64	>64	>64
**3b**	>64	>64	>64	>64	>64	>64	>64
**4a**	>64	32	64	>64	64	>64	>64
**4b**	64	32	64	32	32	16	32
**5**	>64	>64	32	>64	>64	>64	64
**6**	>64	>64	32	>64	>64	>64	2
**8a**	64	>64	32	>64	>64	>64	64
**8b**	32	64	>64	>64	64	>64	32
**8c**	>64	>64	>64	>64	>64	>64	>64
**9a**	64	>64	8	>64	>64	>64	>64
**9b**	32	>64	8	0.5	>64	>64	64
**10**	64	>64	>64	>64	1	>64	>64
**11**	16	>64	>64	>64	8	32	4
**12**	>64	>64	32	0.5	>64	>64	>64
**13**	8	0.5	32	32	32	64	2
**14**	1	0.25	2	0.5	1	4	0.25
**15**	0.5	0.25	0.25	0.25	0.5	64	0.25
**16**	0.5	0.25	0.25	0.25	0.5	16	0.5
**17**	0.5	0.25	0.5	0.25	0.5	16	0.25
**18**	32	>64	16	16	8	16	64
**19**	>64	>64	>64	>64	>64	>64	>64
**20**	32	>64	64	32	64	>64	64
**Ch ^c^**	0.5	4	0.5	0.5	2	2	8

^a^ A. h., *A. hydrophila*; E. c., *E. coli*; M. l., *M. luteus*; S. a., *S. aureus*; V. a., *V. anguillarum*; V. h., *V. harveyi*; V. p., *V. parahaemolyticus*; Ch, Chloramphenicol; ^b^ Average of three replicates; ^c^ Positive control.

### 2.3. Antifungal Activity and SAR Analysis

The inhibitory effects of Compounds **1**–**20** against three plant-pathogenic fungi are summarized in Table 3. Compound **1** exhibited activity against *A. brassicae* with an MIC value of 64 μg/mL. Compounds **4b**, **11**, **13**–**15** and **19** showed activity against *C. gloeosporioides*, and the activity of Compound **14** (MIC = 8 μg/mL) in this assay was twice as potent as that of the positive control (amphotericin B, MIC = 16 μg/mL). In assays against *F. graminearum*, eight compounds, including **1**, **3b**, and **11**–**16**, displayed activity, with MIC values ranging from 0.25 to 64 μg/mL. Among them, Compound **3b** showed particularly noteworthy activity (MIC = 0.25 μg/mL), as it was 128-fold more potent than amphotericin B (MIC = 32 μg/mL) in this assay. Compound **3b** showed extremely high selectively against *F. graminearum* and might have potential as an antifungal agent. Four other compounds (**11**–**15**) were also more potent (two- to eight-fold) than the positive control.

**Table 3:** Minimum inhibition concentration (MIC) of **1** and its derivatives (μg/mL) against three plant-pathogenic fungi ^a^.

Compounds	*A. brassicae*	*C. gloeosporioides*	*F. graminearum*
**1**	64	>64	32
**2a**	>64	>64	>64
**2b**	>64	>64	>64
**3a**	>64	>64	>64
**3b**	>64	>64	0.25
**4a**	>64	>64	>64
**4b**	>64	32	>64
**5**	>64	>64	>64
**6**	>64	>64	>64
**8a**	>64	>64	>64
**8b**	>64	>64	>64
**8c**	>64	>64	>64
**9a**	>64	>64	>64
**9b**	>64	>64	>64
**10**	>64	>64	>64
**11**	>64	32	4
**12**	>64	>64	16
**13**	>64	32	16
**14**	>64	8	8
**15**	>64	16	16
**16**	>64	>64	64
**17**	>64	>64	>64
**18**	>64	>64	>64
**19**	>64	64	>64
**20**	>64	>64	>64
Amphotericin B ^b^	16	16	32

^a^ Average of three replicates; ^b^ positive control.

Based on the above data, newly synthesized Compounds **3b**, **11**, **14** and **15** were found to be the most effective, especially against *F. graminearum*. From an SAR standpoint, it is noteworthy that the oxidation of the methyl to a hydroxymethyl group at C-8 (**2b** *vs.* **3b**) or replacement of the methyl ester group at C-1 by an ethyl ester (**3a** *vs.* **3b**) significantly increased the antifungal activity. Compounds with a reduced double bond at C-6/7 (**11** and **13**–**15**) also showed better inhibitory activities against *C. gloeosporioides* and *F. graminearum* (except for those containing an aldehyde group, such as **17**, **19** and **20**).

## 3. Experimental Section

### 3.1. General

Chemicals and instruments: 2-Methyl-2-hepten-6-one was purchased from TCI Company. *t*-Butyl hydroperoxide (70% in water), BrCH_2_COOCH_3_ and BrCH_2_COOCH_2_CH_3_ were purchased from J&K Company (Qingdao, China). THF was dried over LiAlH_4_ and distilled prior to use. CH_2_Cl_2_ was distilled over CaH_2_. The reagents were all analytically or chemically pure. All solvents and liquid reagents were dried by standard methods in advance and distilled before use according to Perrin and Armarego [24]. Thin-layer chromatography (TLC) was performed using silica gel GF-254 plates (Qing-Dao Chemical Company, Qingdao, China) with detection by UV (254 nm). NMR spectra were recorded on Bruker Advance 500 spectrometers with tetramethylsilane (TMS) as an internal standard. Chemical shifts were reported in parts per million (ppm, δ) downfield from tetramethylsilane. Proton coupling patterns were described as singlet (s), doublet (d), triplet (t), quartet (q), multiplet (m) and broad (br). Mass spectra were recorded by electrospray ionization (ESI) using a VG Autospec 3000 mass spectrometer.

Plant-pathogenic fungi: All strains of fungi were provided by Qindao Agricultural University. The strains were retrieved from a storage tube and incubated in potato dextrose agar media (PDA) at 28 °C for a week to get new mycelia for the antifungal assay.

Bacterial strains: The strains were provided by the Institute of Oceanology, Chinese Academy of Sciences, and cultured at 28 or 37 °C in nutrient agar (NA).

### 3.2. Synthesis of Compounds **1**–**20**

#### 3.2.1. Synthesis of Methyl (±)-3-Hydroxy-3,7-dimethyloct-6-enoate (**2a**)

2-Methyl-2-hepten-6-one (3.16 g, 25.0 mol) and BrCH_2_COOCH_3_ (7.65 g, 50.0 mol) in 50 mL of anhydrous THF were added to a suspension of Zn (3.27 g, 50.0 mol) in anhydrous THF (50 mL) at reflux. After the reaction was initiated, the other portion of the solution was added dropwise to the reaction mixture. The mixture was allowed to stir at reflux until the color of the Zn changed from gray to brownish. After 2 h, the reaction mixture was cooled to room temperature, and the reaction was quenched with 100 mL of 10% AcOH. The reaction mixture was extracted with EtOAc (3 × 100 mL). The combined organic layers were washed with brine and dried with anhydrous Na_2_SO_4_. The solvent was evaporated *in vacuo*, and the crude product was purified by column chromatography (EtOAc–petroleum ether, 1:7) to yield **2a** (4.25 g, 85%) as a pale yellow oil; *Rf* 0.32 (EtOAc–petroleum ether, 1:7); ^1^H NMR (500 MHz, CDCl_3_) δ 5.07 (t, *J* = 6.3 Hz, 1H, CH_2_CH=), 3.69 (s, 3H, OCH_3_), 3.08 (s, 1H, OH), 2.51 (d, *J* = 15.5 Hz, 1H, CH_2_COOCH_3_), 2.43 (d, *J* = 15.5 Hz, 1H, CH_2_COOCH_3_), 2.02 (m, 2H, =CHCH_2_), 1.66 (s, 3H, CH_3_), 1.59 (s, 3H, CH_3_), 1.52 (m, 2H, =CHCH_2_CH_2_), 1.22 (s, 3H, COHCH_3_); ^13^C NMR (125 MHz, CDCl_3_) δ 173.4, 131.9, 124.2, 71.0, 51.7, 44.9, 41.9, 26.8, 25.7, 22.8, 17.7; HRESIMS *m*/*z* 201.1486 [M + H]^+^ (calcd. for C_11_H_21_O_3_, 201.1485).

#### 3.2.2. Synthesis of Ethyl (±)-3-Hydroxy-3,7-dimethyloct-6-enoate (**2b**)

This compound was obtained from BrCH_2_COOCH_2_CH_3_ by a method analogous to that used for **2a**. **2b** (3.05 g, 95%); *Rf* 0.35 (EtOAc–petroleum ether, 1:7); ^1^H NMR (500 MHz, CDCl_3_) δ 5.06 (t, *J* = 6.3 Hz, 1H, CH_2_CH=), 4.14 (q, *J* = 7.1 Hz, 2H, OCH_2_CH_3_), 3.39 (br s, 1H, OH), 2.48 (d, *J* = 15.5 Hz, 1H, CH_2_COOCH_2_CH_3_), 2.40 (d, *J* = 15.5 Hz, 1H, CH_2_COOCH_2_CH_3_), 2.01 (m, 2H, =CHCH_2_), 1.64 (s, 3H, CH_3_), 1.57 (s, 3H, CH_3_), 1.50 (td, *J* = 7.3, 3.5 Hz, 2H, =CHCH_2_CH_2_), 1.24 (t, *J* = 7.1 Hz, 3H, OCH_2_CH_3_), 1.21 (s, 3H, COHCH_3_); ^13^C NMR (125 MHz, CDCl_3_) δ 173.1, 131.8, 124.2, 71.0, 60.7, 45.0, 41.9, 26.7, 25.7, 22.7, 17.6, 14.2; HRESIMS *m*/*z* 215.1640 [M + H]^+^ (calcd. for C_12_H_23_O_3_, 215.1642).

#### 3.2.3. Syntheses of Methyl (*E*)-(±)-3,8-Dihydroxy-3,7-dimethyloct-6-enoate (**3a**) and Methyl (*E*)-(±)-3-Hydroxy-3,7-dimethyl-8-oxooct-6-enoate (**4a**)

Compound **2a** (3.20 g, 15.9 mmol) was dissolved in CH_2_Cl_2_ (150 mL) and added dropwise to a solution of SeO_2_ (825 mg, 7.4 mmol) and *t*-BuOOH (70% in water, 3.2 mL, 22.3 mmol) in CH_2_Cl_2_ (50 mL) at room temperature. After stirring at room temperature for 19 h, the reaction mixture was quenched with aqueous 10% NaOH, H_2_O and brine; then, the organic layer was dried and concentrated. The resulting yellow oil was purified by flash column chromatography (EtOAc–petroleum ether, 1:7) on silica gel to yield **4a** as a colorless oil (624 mg, 25%); *Rf* 0.33 (EtOAc–petroleum ether, 1:3); ^1^H NMR (500 MHz, CDCl_3_) δ 9.38 (s, 1H, CHO), 6.48 (t, *J* = 6.9 Hz, 1H, CH_2_CH=), 3.72 (s, 3H, OCH_3_), 2.55 (d, *J* = 15.7 Hz, 1H, CH_2_COOCH_3_), 2.49 (d, *J* = 15.7 Hz, 1H, CH_2_COOCH_3_), 2.46 (m, 2H, CH_2_CH_2_CH=), 1.75 (s, 3H, CH_3_), 1.67 (m, 2H, CH_2_CH_2_CH=), 1.28 (s, 3H, COHCH_3_); ^13^C NMR (125 MHz, CDCl_3_) δ 195.3, 173.3, 154.3, 139.6, 70.7, 51.9, 44.9, 40.2, 26.7, 23.7, 9.3; HRESIMS *m*/*z* 237.1100 [M + Na]^+^ (calcd. for C_11_H_18_O_4_Na, 237.1097). Compound **3a** was obtained in an analogous fashion; colorless oil (732 mg, 29%); *Rf* 0.22 (EtOAc–petroleum ether, 1:3); ^1^H NMR (500 MHz, CDCl_3_) δ 5.39 (t, *J* = 7.1 Hz, 1H, CH_2_CH=), 3.98 (s, 2H, CH_2_OH), 3.71 (s, 3H, OCH_3_), 2.53 (d, *J* = 15.6 Hz, 1H, CH_2_COOCH_3_), 2.46 (d, *J* = 15.6 Hz, 1H, CH_2_COOCH_3_), 2.12 (m, 2H, CH_2_CH=), 1.66 (s, 3H, CH_3_C=), 1.56 (m, 2H, CH_2_CH_2_CH=), 1.25 (s, 3H, COHCH_3_); ^13^C NMR (125 MHz, CDCl_3_) δ 173.5, 135.3, 125.8, 71.0, 68.9, 51.8, 45.0, 41.6, 26.8, 22.4, 13.7; HRESIMS *m*/*z* 239.1252 [M + Na]^+^ (calcd. for C_11_H_20_O_4_Na, 239.1254). Additionally, 841 mg of material were recovered. The *E*-configuration of Compound **3a** was verified by a NOESY correlation from CH_2_CH= (δ 5.39) to CH_2_OH (δ 3.98).

#### 3.2.4. Reduction of Aldehyde **4a** to Alcohol **3a**

NaBH_4_ (117 mg, 3.1 mmol) was added to a stirred solution of aldehyde **4a** (840 mg, 3.1 mmol) in MeOH (50 mL). The mixture was stirred for 1 h at room temperature, then concentrated under reduced pressure. The residue thus obtained was treated with EtOAc (20 mL) and saturated NH_4_Cl solution (50 mL); then, the separated aqueous phase was extracted with EtOAc (3 × 30 mL). The combined organic phases were dried (Na_2_SO_4_), filtered and concentrated under reduced pressure. The residue was purified by flash column chromatography (EtOAc–petroleum ether, 1:5) on silica gel to yield a colorless oil, **3a** (460 mg, 69%).

#### 3.2.5. Syntheses of Ethyl (*E*)-(±)-3,8-Dihydroxy-3,7-dimethyloct-6-enoate (**3b**) and Ethyl (*E*)-(±)-3-Hydroxy-3,7-dimethyl-8-oxooct-6-enoate (**4b**)

These compounds were obtained from **2b** in a manner similar to that described for the preparation of **3a** and **4a**. Colorless oil **4b** (3.98 g, 16%); *Rf* 0.35 (EtOAc–petroleum ether, 1:3); ^1^H NMR (500 MHz, CDCl_3_) δ 9.39 (s, 1H, CHO), 6.49 (t, *J* = 7.1 Hz, 1H, CH_2_CH=), 4.20 (q, *J* = 7.1 Hz, 2H, OCH_2_CH_3_), 2.54 (d, *J* = 15.8 Hz, 1H, CH_2_COOCH_2_CH_3_), 2.48 (d, *J* = 15.5 Hz, 1H, CH_2_COOCH_2_CH_3_), 2.45 (m, 2H, CH_2_CH=), 1.76 (s, 3H, CH_3_), 1.67 (m, 2H, CH_2_CH_2_CH=), 1.29 (s, 3H, COHCH_3_), 1.29 (t, *J* = 7.0 Hz, 3H, OCH_2_CH_3_); ^13^C NMR (125 MHz, CDCl_3_) δ 195.3, 173.0, 154.3, 139.6, 70.7, 61.0, 45.1, 40.2, 26.7, 23.8, 14.3, 9.3; HRESIMS *m*/*z* 251.1253 [M + Na]^+^ (calcd. for C_12_H_20_O_4_Na, 251.1254). Colorless oil **3b** (20.91 g, 81%); *Rf* 0.25 (EtOAc–petroleum ether, 1:3); ^1^H NMR (500 MHz, CDCl_3_) δ 5.38 (t, *J* = 6.7 Hz, 1H, CH_2_CH=), 4.16 (q, *J* = 7.1 Hz, 2H, OCH_2_CH_3_), 3.97 (s, 2H, CH_2_OH), 2.50 (d, *J* = 15.5 Hz, 1H, CH_2_COOCH_2_CH_3_), 2.43 (d, *J* = 15.6 Hz, 1H, CH_2_COOCH_2_CH_3_), 2.11 (m, 2H, =CHCH_2_), 1.65 (s, 3H, CH_3_), 1.54 (m, 2H, =CHCH_2_CH_2_), 1.26 (t, *J* = 7.2 Hz, 3H, OCH_2_CH_3_), 1.24 (s, 3H, COHCH_3_); ^13^C NMR (125 MHz, CDCl_3_) δ 173.1, 135.2, 125.7, 71.0, 68.9, 60.8, 45.1, 41.6, 26.7, 22.3, 14.3, 13.7; HRESIMS *m*/*z* 253.1415 [M + Na]^+^ (calcd. for C_12_H_22_O_4_Na, 253.1410).

#### 3.2.6. Synthesis of (*E*)-(±)-3,8-Dihydroxy-3,7-dimethyloct-6-enoic Acid (**5**)

To a solution of **3a** (133 mg, 0.6 mmol) in MeOH (30 mL) and water (10 mL) was added KOH (76 mg, 1.4 mmol). The resulting reaction mixture was heated under reflux for 3 h, and the reaction was monitored by TLC. After completion of the reaction, the reaction mixture was acidified with 1 N HCl to pH 2~3, followed by extraction with CHCl_3_ (3 × 20 mL). The combined organic layers were dried (MgSO_4_) and concentrated *in vacuo*. The residue was chromatographed over silica gel (EtOAc–petroleum ether, 1:1) to afford the acid, **5** (105 mg, 85%), as a colorless oil; *Rf* 0.25 (EtOAc:CHCl_3_, 1:1); ^1^H NMR (500 MHz, CDCl_3_) δ 5.41 (t, *J* = 5.4 Hz, 1H, CH_2_CH=), 4.00 (s, 2H, CH_2_OH), 2.58 (d, *J* = 15.7 Hz, 1H, CH_2_COOH), 2.51 (d, *J* = 15.7 Hz, 1H, CH_2_COOH), 2.13 (m, 2H, CH_2_CH=), 1.67 (s, 3H, CH_3_C=), 1.63 (m, 2H, CH_2_CH_2_CH=), 1.31 (s, 3H, COHCH_3_); ^13^C NMR (125 MHz, CDCl_3_) δ 175.5, 135.4, 125.7, 71.5, 68.9, 44.9, 41.5, 26.8, 22.4, 13.8; HRESIMS *m*/*z* 225.1076 [M + Na]^+^ (calcd. for C_10_H_18_O_4_Na, 225.1103).

#### 3.2.7. Syntheses of Acetic (*E*)-(±)-8-Acetoxy-3-hydroxy-3,7-dimethyloct-6-enoic Anhydride (**10**) and Penicimonoterpene (±)-**1**

Compound **5** (250 mg, 1.2 mmol) was dissolved in CH_2_Cl_2_ (20 mL) at room temperature. To this solution were added DMAP (3 mg, 0.03 mmol), Et_3_N (225 μL, 1.6 mmol) and, finally, Ac_2_O (152 μL, 1.6 mmol). The reaction was stirred overnight. After completion of the reaction as detected by TLC, the solvents were evaporated under reduced pressure, and the crude product was purified by column chromatography (EtOAc–petroleum ether, 1:1) to afford **10** and (±)-**1**.

**10**, colorless oil (65 mg, 18%); *Rf* 0.75 (EtOAc–petroleum ether, 1:3); ^1^H NMR (500 MHz, CDCl_3_) δ 5.44 (t, *J* = 6.5 Hz, 1H, CH_2_CH=), 4.44 (s, 2H, CH_2_OAc), 3.05 (d, *J* = 14.5 Hz, 1H, CH_2_COOAc), 2.90 (d, *J* = 14.5 Hz, 1H, CH_2_COOAc), 2.10 (m, 2H, CH_2_CH=), 2.07 (s, 3H, COCH_3_), 2.03 (m, 1H, CH_2_CH_2_CH=), 2.00 (s, 3H, CH_3_COOCOCH_2_), 1.81 (m, 1H, CH_2_CH_2_CH=), 1.65 (s, 3H, CH_3_C=), 1.55 (s, 3H, COHCH_3_); ^13^C NMR (125 MHz, CDCl_3_) δ 175.4, 171.2, 170.7, 130.9, 128.4, 81.2, 70.1, 42.5, 38.3, 24.1, 22.3, 22.1, 21.1, 14.0; HRESIMS *m/z* 309.1307 [M + Na]^+^ (calcd. for C_14_H_22_O_6_Na, 309.1309).

Penicimonoterpene (±)-**1**, colorless oil (165 mg, 55%); *Rf* 0.62 (EtOAc–petroleum ether, 2:1); ^1^H NMR (500 MHz, CDCl_3_) δ 5.39 (t, *J* = 6.7 Hz, 1H, CH_2_CH=), 4.38 (s, 2H, CH_2_O), 2.51 (d, *J* = 15.7 Hz, 1H, CH_2_COOH), 2.45 (d, *J* = 15.7 Hz, 1H, CH_2_COOH), 2.08 (m, 2H, CH_2_CH=), 2.01 (s, 3H, COCH_3_), 1.60 (s, 3H, CH_3_C=), 1.55 (m, 2H, CH_2_CH_2_CH=), 1.24 (s, 3H, COHCH_3_); ^13^C NMR (125 MHz, CDCl_3_) δ 176.4, 171.5, 130.4, 129.0, 71.3, 70.3, 44.6, 41.0, 26.4, 22.3, 21.0, 13.8; HRESIMS *m/z* 267.1202 [M + Na]^+^ (calcd. for C_12_H_20_O_5_Na, 267.1203).

The spectral data (NMR, MS and HRMS) of (±)-**1** were in agreement with those of the reported natural penicimonoterpene [15].

#### 3.2.8. Synthesis of (±)-3-Hydroxy-3,7-dimethyloct-6-enoic Acid (**6**)

Compound **6** was obtained from **2a** by a method similar to that described for the preparation of **5**. Colorless oil **6** (443 mg, 94%); *Rf* 0.23 (EtOAc–CHCl_3_, 1:1); ^1^H NMR (500 MHz, CDCl_3_) δ 5.10 (t, *J* = 6.6 Hz, 1H, =CHCH_2_), 2.59 (d, *J* = 15.7 Hz, 1H, CH_2_COOH), 2.51 (d, *J* = 15.7 Hz, 1H, CH_2_COOH), 2.07 (m, 2H, =CHCH_2_), 1.68 (s, 3H, CH_3_), 1.61 (s, 3H, CH_3_), 1.58 (m, 2H, =CHCH_2_CH_2_), 1.30 (s, 3H, CH_3_COH); ^13^C NMR (125 MHz, CDCl_3_) δ 177.2, 132.3, 123.9, 71.5, 44.9, 41.8, 26.7, 25.8, 22.8, 17.8; HRESIMS *m*/*z* 187.1332 [M + H]^+^ (calcd. for C_10_H_19_O_3_, 187.1329).

#### 3.2.9. Synthesis of (*E*)-7-Hydroxy-6-methylhept-5-en-2-one (**7**)

Compound **7** was obtained from 2-methyl-2-hepten-6-one in a manner similar to that described for the preparation of **3a**. Yellow oil **7** (1.35 g, 38%); *Rf* 0.15 (EtOAc–petroleum ether, 1:5); ^1^H NMR (500 MHz, DMSO-*d*_6_) δ 5.26 (t, *J* = 7.2 Hz, 1H, =CHCH_2_), 4.64 (s, 1H, CH_2_OH), 3.75 (s, 2H, CH_2_OH), 2.46 (t, *J* = 7.4 Hz, 2H, CH_2_COCH_3_), 2.16 (q, *J* = 7.2 Hz, 2H, =CHCH_2_), 2.07 (s, 3H, COCH_3_), 1.54 (s, 3H, CH_3_); ^13^C NMR (125 MHz, DMSO-*d*_6_) δ 208.0, 135.9, 122.1, 66.2, 42.6, 29.6, 21.4, 13.4.

#### 3.2.10. Synthesis of Methyl (*E*)-(±)-8-Acetoxy-3-hydroxy-3,7-dimethyloct-6-enoate (**8a**)

Compound **3a** (130 mg, 0.6 mmol) was dissolved in CH_2_Cl_2_ (5 mL) at room temperature. To this solution were added DMAP (2 mg, 0.02 mmol), Et_3_N (125 μL, 0.9 mmol) and Ac_2_O (85 μL, 0.9 mmol). The reaction was stirred for 24 h. After completion of the reaction as detected by TLC, the solvents were evaporated under reduced pressure, and the crude products were purified by column chromatography (EtOAc–petroleum ether, 1:1) to afford **8a** (141 mg, 91%) as a colorless oil; *Rf* 0.72 (EtOAc–petroleum ether, 1:3); ^1^H NMR (500 MHz, CDCl_3_) δ 5.42 (t, *J* = 7.1 Hz, 1H, CH_2_CH=), 4.41 (s, 2H, CH_2_O), 3.69 (s, 3H, OCH_3_), 2.51 (d, *J* = 15.6 Hz, 1H, CH_2_COOCH_3_), 2.44 (d, *J* = 15.6 Hz, 1H, CH_2_COOCH_3_), 2.12 (m, 2H, CH_2_CH=), 2.04 (s, 3H, COCH_3_), 1.63 (s, 3H, CH_3_C=), 1.53 (m, 2H, CH_2_CH_2_CH=), 1.23 (s, 3H, COHCH_3_); ^13^C NMR (125 MHz, CDCl_3_) δ 172.7, 170.4, 129.8, 128.6, 70.2, 69.5, 51.1, 44.2, 40.7, 26.1, 21.7, 20.4, 13.3; HRESIMS *m*/*z* 281.1359 [M + Na]^+^ (calcd. for C_13_H_22_O_5_Na, 281.1359).

#### 3.2.11. Synthesis of Ethyl (*E*)-(±)-8-Acetoxy-3-hydroxy-3,7-dimethyloct-6-enoate (**8b**)

Compound **8b** was obtained from **3b** by a method similar to that described for the preparation of **8a**. Colorless oil **8b** (250 mg, 95%); *Rf* 0.75 (EtOAc–petroleum ether, 1:3); ^1^H NMR (500 MHz, CDCl_3_) δ 5.43 (t, *J* = 7.0 Hz, 1H, CH_2_CH=), 4.43 (s, 2H, CH_3_COOCH_2_), 4.16 (q, *J* = 7.1 Hz, 2H, OCH_2_CH_3_), 3.24 (br s, 1H, COHCH_3_), 2.50 (d, *J* = 15.6 Hz, 1H, CH_2_COOCH_2_CH_3_), 2.43 (d, *J* = 15.6 Hz, 1H, CH_2_COOCH_2_CH_3_), 2.13 (m, 2H, CH_2_CH=), 2.05 (s, 3H, CH_3_COOCH_2_), 1.64 (s, 3H, CH_3_C=), 1.54 (m, 2H, CH_2_CH_2_CH=), 1.26 (t, *J* = 7.1 Hz, 3H, OCH_2_CH_3_), 1.23 (s, 3H, COHCH_3_); ^13^C NMR (125 MHz, CDCl_3_) δ 173.0, 171.0, 130.5, 129.3, 70.9, 70.2, 60.8, 45.1, 41.4, 26.7, 22.4, 21.1, 14.3, 14.0; HRESIMS *m*/*z* 295.1518 [M + Na]^+^ (calcd. for C_14_H_24_O_5_Na, 295.1516).

#### 3.2.12. Synthesis of Ethyl (*E*)-(±)-8-((*R*)-2-Acetoxy-2-phenylacetoxy)-3-hydroxy-3,7-dimethyloct-6-enoate (**8c**)

DCC (563 mg, 2.7 mmol) was dissolved in CH_2_Cl_2_ (20 mL), and *R*-(−)-*O*-acyl mandelic acid (530 mg, 2.7 mmol) and DMAP (30 mg, 0.3 mmol) were added at room temperature. After stirring for 16 h at room temperature, the solvent volume was reduced *in vacuo*, and the remaining mixture was purified by column chromatography (EtOAc–petroleum ether, 1:8) to afford **8c** (819 mg, 81%) as a colorless oil; *Rf* 0.76 (EtOAc–petroleum ether, 1:2); ^1^H NMR (500 MHz, CDCl_3_) δ 7.47 (dd, *J* = 6.4, 2.8 Hz, 2H, ArH), 7.38 (m, 3H, ArH), 5.92 (s, 1H, CHArH), 5.33 (t, *J* = 6.9 Hz, 1H, CH_2_CH=), 4.50 (dd, *J* = 12.2 Hz, 2H, COOCH_2_CCH_3_=), 4.18 (q, *J* = 7.1 Hz, 2H, OCH_2_CH_3_), 2.49 (d, *J* = 15.6 Hz, 1H, CH_2_COOCH_2_CH_3_), 2.42 (d, *J* = 15.6 Hz, 1H, CH_2_COOCH_2_CH_3_), 2.19 (s, 3H, CH_3_COO), 2.06 (m, 2H, CH_2_CH=), 1.50 (s, 3H, CH_3_C=), 1.48 (m, 2H, CH_2_CH_2_CH=), 1.28 (t, *J* = 7.1 Hz, 3H, OCH_2_CH_3_), 1.22 (s, 3H, COHCH_3_); ^13^C NMR (125 MHz, CDCl_3_) δ 173.1, 170.4, 168.8, 134.1, 129.9, 129.7, 129.3, 128.9 (two), 127.8 (two), 74.7, 71.1, 70.8, 60.8, 45.1, 41.3, 26.7, 22.4, 20.8, 14.3, 13.7; HRESIMS *m*/*z* 407.2065 [M + H]^+^ (calcd. for C_22_H_31_O_7_, 407.2064).

#### 3.2.13. Synthesis of Methyl (*E*)-(±)-8-Chloro-3-hydroxy-3,7-dimethyloct-6-enoate (**9a**)

To a solution of **3a** (76 mg, 0.3 mmol) and PPh_3_ (138 mg, 0.5 mmol) in 10 mL of dry CH_2_Cl_2_ was added NCS (70 mg, 0.5 mmol) at 0 °C under nitrogen. The reaction mixture was stirred at 0 °C for 1 h, then allowed to warm to room temperature and stirred for 2 h. The reaction was monitored by TLC. After completion of the reaction, the solvent volume was reduced *in vacuo*, and the remaining mixture was purified by column chromatography (EtOAc–petroleum ether, 1:5) to afford **9a** (63 mg, 77%) as a colorless oil; *Rf* 0.63 (EtOAc–petroleum ether, 1:3); ^1^H NMR (500 MHz, CDCl_3_) δ 5.49 (t, *J* = 7.0 Hz, 1H, CH_2_CH=), 3.97 (s, 2H, CH_2_Cl), 3.69 (s, 3H, OCH_3_), 3.20 (br s, 1H, COHCH_3_), 2.50 (d, *J* = 15.6 Hz, 1H, CH_2_COOCH_3_), 2.43 (d, *J* = 15.6 Hz, 1H, CH_2_COOCH_3_), 2.11 (m, 2H, CH_2_CH=), 1.71 (s, 3H, CH_3_C=), 1.55 (m, 2H, CH_2_CH_2_CH=), 1.22 (s, 3H, COHCH_3_); ^13^C NMR (125 MHz, CDCl_3_) δ 173.3, 132.1, 130.4, 70.8, 52.3, 51.7, 44.9, 41.1, 26.7, 22.7, 14.1; HRESIMS *m*/*z* 257.0892 [M + Na]^+^ (calcd. for C_11_H_19_ClO_3_Na, 257.0920).

#### 3.2.14. Synthesis of Ethyl (*E*)-(±)-8-Chloro-3-hydroxy-3,7-dimethyloct-6-enoate (**9b**)

This compound was obtained from **3b** by a method similar to that used for **9a**. Colorless oil **9b** (157 mg, 69%); *Rf* 0.82 (EtOAc–petroleum ether, 1:2); ^1^H NMR (500 MHz, CDCl_3_) δ 5.51 (t, *J* = 7.0 Hz, 1H, CH_2_CH=), 4.17 (q, *J* = 7.1 Hz, 2H, OCH_2_CH_3_), 3.99 (s, 2H, CH_2_Cl), 3.17 (br s, 1H, COHCH_3_), 2.50 (d, *J* = 15.6 Hz, 1H, CH_2_COOCH_2_CH_3_), 2.43 (d, *J* = 15.6 Hz, 1H, CH_2_COOCH_2_CH_3_), 2.13 (m, 2H, CH_2_CH=), 1.73 (s, 3H, CH_3_C=), 1.55 (m, 2H, CH_2_CH_2_CH=), 1.27 (t, *J* = 7.1 Hz, 3H, OCH_2_CH_3_), 1.24 (s, 3H, COHCH_3_); ^13^C NMR (125 MHz, CDCl_3_) δ 173.0, 132.1, 130.5, 70.8, 60.8, 52.4, 45.1, 41.2, 26.8, 22.8, 14.3, 14.2; HRESIMS *m/z* 249.1253 [M + H]^+^ (calcd. for C_12_H_22_ClO_3_, 249.1252).

#### 3.2.15. Synthesis of (±)-3,7-Dimethyloct-6-ene-1,3-diol (**12**)

Compound **2a** (100 mg, 0.5 mmol) in THF (10 mL) was added dropwise to a stirred slurry of LiAlH_4_ (57 mg, 1.5 mmol) in THF (10 mL) in an ice bath. After stirring in an ice bath for 2 h, the solution was heated to 65 °C about 10 h. The reaction was monitored by TLC. After completion of the reaction, the reaction mixture was successively treated with water (10 mL), 20% aqueous NaOH (10 mL) and then again with water (10 mL). The resulting white granular suspension was filtered, and the filter cake was washed with CHCl_3_ (3 × 10 mL). The organic layers were combined, dried (Na_2_SO_4_), and then, the solvent was evaporated under reduced pressure and the residue purified by chromatography (EtOAc–petroleum ether, 1:4) to afford **12** (83 mg, 97%) as a colorless oil; *Rf* 0.34 (EtOAc–petroleum ether, 1:2); ^1^H NMR (500 MHz, CDCl_3_) δ 5.09 (t, 1H, CH_2_CH=), 3.84 (m, 2H, CH_2_OH), 3.30 (s, 2H, 2 × OH), 2.01 (m, 2H, CH_2_CH=), 1.76 (m, 1H, CH_2_CH_2_OH), 1.66 (s, 3H, CH_3_),1.63 (m, 1H, CH_2_CH_2_OH), 1.59 (s, 3H, CH_3_), 1.51 (m, 2H, CH_2_CH_2_CH=), 1.21 (s, 3H, COHCH_3_); ^13^C NMR (125 MHz, CDCl_3_) δ 131.9, 124.4, 73.9, 59.7, 42.5, 41.7, 26.7, 25.7, 22.8, 17.7; HRESIMS *m*/*z* 195.1335 [M + Na]^+^ (calcd. for C_10_H_20_O_2_Na, 195.1361).

#### 3.2.16. Synthesis of Acetic (±)-3-Hydroxy-3,7-dimethyloct-6-enoic Anhydride (**18**)

Compound **6** (345 mg, 1.8 mmol) was dissolved in CH_2_Cl_2_ (20 mL) at room temperature. To this solution were added DMAP (23 mg, 0.18 mmol), Et_3_N (517 μL, 3.7 mmol) and, finally, Ac_2_O (350 μL, 3.7 mmol). The reaction was stirred overnight. After completion of the reaction as detected by TLC, the solvents were evaporated under reduced pressure, and the crude product was purified by column chromatography (EtOAc–petroleum ether, 1:10) to afford **18** as a colorless oil (360 mg, 85%); *Rf* 0.75 (EtOAc–petroleum ether, 1:3); ^1^H NMR (500 MHz, CDCl_3_) δ 5.07 (t, *J* = 6.5 Hz, 1H, CH_2_CH=), 3.04 (d, *J* = 14.3 Hz, 1H, CH_2_COOCOCH_3_), 2.89 (d, *J* = 14.3 Hz, 1H, CH_2_COOCOCH_3_), 2.00 (s, 3H, COCH_3_), 1.99 (m, 2H, CH_2_CH=), 1.99 (m, 1H, CH_2_CH_2_CH=), 1.76 (m, 1H, CH_2_CH_2_CH=), 1.67 (s, 3H, CH_3_C=), 1.59 (s, 3H, CH_3_C=), 1.54 (s, 3H, COHCH_3_); ^13^C NMR (125 MHz, CDCl_3_)δ 176.1, 170.8, 132.2, 123.5, 81.4, 42.5, 38.9, 25.8, 24.1, 22.4, 22.3, 17.7; HRESIMS *m*/*z* 251.1260 [M + Na]^+^ (calcd. for C_12_H_20_O_4_Na, 251.1254).

#### 3.2.17. Syntheses of Compounds **11**, **13**–**17**, **19** and **20**

General procedure: A mixture of the starting material (*i.e.*, a double-bond-containing monoterpene derivative) with 10% Pd/C in EtOAc was stirred at room temperature for an appropriate time (12 to 36 h). After the catalyst was removed by filtration, the filtrate was concentrated *in vacuo* to give a residue, which was purified by column chromatography on silica gel.

(±)-3-Hydroxy-3,7-dimethyloctanoic acid (**11**): Colorless oil (47 mg, 92%); *Rf* 0.52 (EtOAc–petroleum ether, 1:1); ^1^H NMR (500 MHz, CDCl_3_) δ 2.57 (d, *J* = 15.7 Hz, 1H, CH_2_COOH), 2.49 (d, *J* = 15.7 Hz, 1H, CH_2_COOH), 1.55 (m, 1H, CH_2_CH(CH_3_)_2_), 1.51 (m, 2H, CH_2_COH), 1.34 (m, 2H, CH_2_CH_2_CH_2_), 1.28 (s, 3H, COHCH_3_), 1.16 (m, 2H, CH_2_CH_2_CH_2_), 0.87 (s, 3H, CH(CH_3_)_2_), 0.86 (s, 3H, CH(CH_3_)_2_); ^13^C NMR (125 MHz, CDCl_3_) δ 177.2, 71.7, 44.8, 42.4, 39.4, 28.0, 26.7, 22.7, 22.6, 21.8; HRESIMS *m*/*z* 187.1353 [M − H]^−^ (calcd. for C_10_H_19_O_3_, 187.1340).

Ethyl (±)-3-hydroxy-3,7-dimethyloctanoate (**13**): Colorless oil (61 mg, 96%); *Rf* 0.73 (EtOAc–petroleum ether, 1:5); ^1^H NMR (500 MHz, CDCl_3_) δ 4.17 (q, *J* = 7.1 Hz, 2H, OCH_2_CH_3_), 2.50 (d, *J* = 15.5 Hz, 1H, CH_2_COOCH_2_CH_3_), 2.42 (d, *J* = 15.5 Hz, 1H, CH_2_COOCH_2_CH_3_), 1.53 (m, 1H, CH(CH_3_)_2_), 1.47 (m, 2H, CH_2_CH_2_CH_2_COHCH_3_), 1.33 (m, 2H, CH_2_CH_2_CH_2_COHCH_3_), 1.27 (t, *J* = 7.1 Hz, 3H, OCH_2_CH_3_), 1.22 (s, 3H, COHCH_3_), 1.15 (m, 2H, CH_2_CH_2_CH_2_COHCH_3_), 0.87 (s, 3H, CH(CH_3_)_2_), 0.86 (s, 3H, CH(CH_3_)_2_); ^13^C NMR (125 MHz, CDCl_3_) δ 173.2, 71.2, 60.7, 45.0, 42.5, 39.5, 28.0, 26.8, 22.7, 22.6, 21.8, 14.3; HRESIMS *m*/*z* 239.1624 [M + Na]^+^ (calcd. for C_12_H_24_O_3_Na, 239.1618).

Acetic (±)-3-hydroxy-3,7-dimethyloctanoic anhydride (**14**): Colorless oil (45 mg, 93%); *Rf* 0.75 (EtOAc–petroleum ether, 1:1); ^1^H NMR (500 MHz, CDCl_3_) δ 3.02 (d, *J* = 14.4 Hz, 1H, CH_2_COOCOCH_3_), 2.88 (d, *J* = 14.4 Hz, 1H, CH_2_COOCOCH_3_), 1.99 (s, 3H, COCH_3_), 1.93 (m, 1H, CH_2_CH_2_CH_2_COHCH_3_), 1.73 (m, 1H, CH_2_CH_2_CH_2_COHCH_3_), 1.53 (m, 1H, CH(CH_3_)_2_), 1.52 (s, 3H, COHCH_3_), 1.31 (m, 2H, CH_2_CH_2_CH_2_COHCH_3_), 1.17 (m, 2H, CH_2_CH_2_CH_2_COHCH_3_), 0.87 (s, 3H, CH(CH_3_)_2_), 0.86 (s, 3H, CH(CH_3_)_2_); ^13^C NMR (125 MHz, CDCl_3_) δ 176.2, 170.8, 81.6, 42.6, 39.2, 39.1, 27.9, 24.2, 22.7, 22.4, 21.3; HRESIMS *m*/*z* 253.1409 [M + Na]^+^ (calcd. for C_12_H_22_O_4_Na, 253.1410).

Methyl (±)-3-hydroxy-3,7-dimethyloctanoate (**15**): Colorless oil (45 mg, 93%); *Rf* 0.72 (EtOAc–petroleum ether, 1:3); ^1^H NMR (500 MHz, CDCl_3_) δ 3.71 (s, 3H, OCH_3_), 3.41 (br s, 1H, COHCH_3_), 2.52 (d, *J* = 15.1 Hz, 1H, CH_2_COOCH_3_), 2.45 (d, *J* = 15.6 Hz, 1H, CH_2_COOCH_3_), 1.54 (m, 1H, CH(CH_3_)_2_), 1.46 (m, 2H, CH_2_CH_2_CH_2_COHCH_3_), 1.33 (m, 2H, CH_2_CH_2_CH_2_COHCH_3_), 1.23 (s, 3H, COHCH_3_), 1.16 (m, 2H, CH_2_CH_2_CH_2_COHCH_3_), 0.87 (s, 3H, CH(CH_3_)_2_), 0.86 (s, 3H, CH(CH_3_)_2_); ^13^C NMR (125 MHz, CDCl_3_) δ 173.6, 71.2, 51.7, 44.9, 42.5, 39.5, 28.1, 26.9, 22.7, 22.6, 21.8; HRESIMS *m/z* 225.1461 [M + Na]^+^ (calcd. for C_11_H_22_O_3_Na, 225.1461).

(±)-3,7-Dimethyloctane-1,3-diol (**16**): Colorless oil (26 mg, 89%); *Rf* 0.52 (EtOAc–petroleum ether, 1:3); ^1^H NMR (500 MHz, CDCl_3_) δ 3.88 (m, 2H, CH_2_OH), 2.49 (s, 2H, 2 × OH), 1.79 (m, 1H, CH_2_CH_2_OH), 1.66 (m, 1H, CH_2_CH_2_OH), 1.55 (m, 1H, CH(CH_3_)_2_), 1.50 (m, 2H, CH_2_CH_2_CH_2_COHCH_3_), 1.31 (m, 2H, CH_2_CH_2_CH_2_COHCH_3_), 1.24 (s, 3H, COHCH_3_), 1.18 (m, 2H, CH_2_CH_2_CH_2_COHCH_3_), 0.88 (s, 3H, CH(CH_3_)_2_), 0.87 (s, 3H, CH(CH_3_)_2_); ^13^C NMR (125 MHz, CDCl_3_) δ 74.2, 60.1, 43.2, 41.7, 39.6, 28.1, 26.9, 22.7, 22.7, 21.9; HRESIMS *m/z* 197.1518 [M + Na]^+^ (calcd. for C_10_H_22_O_2_Na, 197.1512).

(±)-3-Hydroxy-3,7-dimethyl-8-oxooctanoic acid (**17**): Colorless oil (90 mg, 45%); *Rf* 0.33 (EtOAc–petroleum ether, 2:1); ^1^H NMR (500 MHz, CDCl_3_) δ 9.60 (s, 1H, CHO), 2.55 (d, *J* = 15.7 Hz, 1H, CH_2_COOH), 2.48 (d, *J* = 15.7 Hz, 1H, CH_2_COOH), 2.35 (m, 1H, CH_3_CHCHO), 1.70 (m, 1H, CH_2_CH_2_CH_2_COHCH_3_), 1.54 (m, 2H, CH_2_CH_2_CH_2_COHCH_3_), 1.41 (m, 2H, CH_2_CH_2_CH_2_COHCH_3_), 1.38 (m, 1H, CH_2_CH_2_CH_2_COHCH_3_), 1.26 (s, 3H, COHCH_3_), 1.09 (d, *J* = 7.0 Hz, 3H, CH_3_CHCHO); ^13^C NMR (125 MHz, CDCl_3_) δ 205.5, 177.0, 71.3, 46.3, 44.8, 41.9, 30.8, 26.7, 21.4, 13.5; HRESIMS *m*/*z* 225.1101 [M + Na]^+^ (calcd. for C_10_H_18_O_4_Na, 225.1097).

Methyl (±)-3-hydroxy-3,7-dimethyl-8-oxooctanoate (**19**): Colorless oil (94 mg, 27%); *Rf* 0.53 (EtOAc–petroleum ether, 1:1); ^1^H NMR (500 MHz, CDCl_3_) δ 9.61 (s, 1H, CHO), 3.71 (s, 3H, OCH_3_), 2.50 (d, *J* = 15.6 Hz, 1H, CH_2_COOCH_3_), 2.43 (d, *J* = 15.6 Hz, 1H, CH_2_COOCH_3_), 2.34 (m, 1H, CHCH_3_), 1.70 (m, 1H, CHCH_2_CH_2_CH_2_), 1.50 (m, 2H, CHCH_2_CH_2_CH_2_), 1.39 (m, 1H, CHCH_2_CH_2_CH_2_), 1.38 (m, 2H, CHCH_2_CH_2_CH_2_), 1.22 (s, 3H, COHCH_3_ ), 1.09 (d, *J* = 7.0 Hz, 3H, CHCH_3_); ^13^C NMR (125 MHz, CDCl_3_) δ 205.1, 173.5, 70.9, 51.8, 46.4, 44.8, 42.0, 31.0, 26.8, 21.4, 13.5; HRESIMS *m*/*z* 239.1260 [M + Na]^+^ (calcd. for C_11_H_20_O_4_Na, 239.1254).

Ethyl (±)-3-hydroxy-3,7-dimethyl-8-oxooctanoate (**20**): Colorless oil (15 mg, 25%); *Rf* 0.52 (EtOAc–petroleum ether, 1:2); ^1^H NMR (500 MHz, CDCl_3_) δ 9.56 (s, 1H, CHO), 4.12 (q, *J* = 7.1 Hz, 2H, OCH_2_CH_3_), 2.44 (d, *J* = 15.5 Hz, 1H, CH_2_COOCH_2_CH_3_), 2.37 (d, *J* = 15.5 Hz, 1H, CH_2_COOCH_2_CH_3_), 2.30 (m, 1H, CHCH_3_), 1.64 (m, 1H, CHCH_2_CH_2_CH_2_), 1.46 (m, 2H, CHCH_2_CH_2_CH_2_), 1.35 (m, 1H, CHCH_2_CH_2_CH_2_), 1.34 (m, 2H, CHCH_2_CH_2_CH_2_), 1.22 (t, *J* = 7.1 Hz, 3H, OCH_2_CH_3_), 1.17 (s, 3H, COHCH_3_), 1.05 (d, *J* = 7.0 Hz, 3H, CHCH_3_); ^13^C NMR (125 MHz, CDCl_3_) δ 205.0, 172.9, 70.9, 60.7, 46.3, 45.0, 41.9, 30.9, 26.7, 21.4, 14.2, 13.4; HRESIMS *m*/*z* 231.1595 [M + H]^+^ (calcd. for C_12_H_23_O_4_, 231.1591).

### 3.3. Antibacterial Assay

Antibacterial assays against *V. anguillarum*, *A. hydrophila*, *V. parahaemolyticus*, *V. harveyi*, *S. aureus*, *M. luteus* and *E. coli* were carried out using the well diffusion method [25]. Chloramphenicol was used as a positive control.

### 3.4. Antifungal Assay

Antifungal assays against *A. brassicae*, *C. gloeosporioides* and *F. graminearum* were carried out using the well diffusion method [26]. Amphotericin B was used as a positive control.

## 4. Conclusions

We describe herein the first racemic synthesis of penicimonoterpene (±)-**1** and 24 related derivatives. The structures of all of these products were confirmed on the basis of NMR and HRESIMS analysis. Compounds **3a**–**b**, **4a**–**b**, **8a**–**c**, **9a**–**b**, **10**, **14** and **17**–**20** were new compounds. Bioassay results revealed that Compounds **6** and **11** exhibited strong antibacterial activity against *S. aureus*, while Compound **3b** showed extremely high selectively against plant-pathogenic fungus *F. graminearum* (MIC = 0.25 μg/mL). Compound **10** presented extremely surprising selectivity against pathogenic bacteria *E. coli* (MIC = 1 μg/mL). Compound **13** showed high selectivity against *V. anguillarum* and *S. aureus*, which is much better than chloramphenicol, a well-known commercial antibiotic. Compound **14** also exhibited higher antibacterial activity than chloramphenicol against *V. anguillarum*, *E. coli* and *S. aureus*. Compounds **15**, **16** and **17** displayed higher antibacterial activity than chloramphenicol against *V. anguillarum*, *A. hydrophila*, *V. parahaemolyticus*, *V. harveyi*, *E. coli* and *S. aureus*. Taken together, Compounds **15**, **16** and **17** were demonstrated to possess the most potent inhibition of the bacteria tested in the present study, especially for marine *Vibrio* spp. (*V. anguillarum*, *V. harveyi* and *V. parahaemolyticus*), and might have potential as antibiotics or lead compounds.

## Supplementary Files

Supplementary File 1 Supplementary Information (PDF, 5145 KB)
