# Supplementary material for: First Total Syntheses and Antimicrobial Evaluation of Penicimonoterpene, a Marine-Derived Monoterpenoid, and Its Various Derivatives
Source: Mar Drugs. 2014 Jun 3;12(6):3352–70. doi: 10.3390/md12063352 (PMC4071580; doi:10.3390/md12063352)
Supplement: Supplementary File 1 — Supplementary Information (PDF, 5145 KB) [file marinedrugs-12-03352-s001.pdf]

Supplementary Information

Figure S1. ^{1}H NMR spectrum of compound **1**.
Figure S2. ^{13}C NMR spectrum of compound **1**.
Figure S3. HRMS spectrum of compound **1**.
Figure S4. ^{1}H NMR spectrum of compound **2a**.
Figure S5. ^{13}C NMR spectrum of compound **2a**.
Figure S6. HRMS spectrum of compound **2a**.
Figure S7. ^{1}H NMR spectrum of compound **2b**.
Figure S8. ^{13}C NMR spectrum of compound **2b**.
Figure S9. HRMS spectrum of compound **2b**.
Figure S10. ^{1}H NMR spectrum of compound **3a**.
Figure S11. ^{13}C NMR spectrum of compound **3a**.
Figure S12. NOESY spectrum of compound **3a**.
Figure S13. An enlarged area of the NOESY spectrum of compound **3a**.
Figure S14. HRMS spectrum of compound **3a**.
Figure S15. ^{1}H NMR spectrum of compound **3b**.
Figure S16. ^{13}C NMR spectrum of compound **3b**.
Figure S17. HRMS spectrum of compound **3b**.
Figure S18. ^{1}H NMR spectrum of compound **4a**.
Figure S19. ^{13}C NMR spectrum of compound **4a**.
Figure S20. HRMS spectrum of compound **4a**.
Figure S21. ^{1}H NMR spectrum of compound **4b**.
Figure S22. ^{13}C NMR spectrum of compound **4b**.
Figure S23. HRMS spectrum of compound **4b**.
Figure S24. ^{1}H NMR spectrum of compound **5**.
Figure S25. ^{13}C NMR spectrum of compound **5**.
Figure S26. HRMS spectrum of compound **5**.
Figure S27. ^{1}H NMR spectrum of compound **6**.
Figure S28. ^{13}C NMR spectrum of compound **6**.
Figure S29. HRMS spectrum of compound **6**.
Figure S30. ^{1}H NMR spectrum of compound **7**.
Figure S31. ^{13}C NMR spectrum of compound **7**.
Figure S32. ^{1}H NMR spectrum of compound **8a**.
Figure S33. ^{13}C NMR spectrum of compound **8a**.
Figure S34. HRMS spectrum of compound **8a**.
Figure S35. ^{1}H NMR spectrum of compound **8b**.
Figure S36. ^{13}C NMR spectrum of compound **8b**.
Figure S37. HRMS spectrum of compound **8b**.
Figure S38. ^{1}H NMR spectrum of compound **8c**.
Figure S39. ^{13}C NMR spectrum of compound **8c**.
Figure S40. HRMS spectrum of compound **8c**.
Figure S41. ^{1}H NMR spectrum of compound **9a**.

Figure S42. ^{13}C NMR spectrum of compound **9a**.
Figure S43. HRMS spectrum of compound **9a**.
Figure S44. ^{1}H NMR spectrum of compound **9b**.
Figure S45. ^{13}C NMR spectrum of compound **9b**.
Figure S46. HRMS spectrum of compound **9b**.
Figure S47. ^{1}H NMR spectrum of compound **10**.
Figure S48. ^{13}C NMR spectrum of compound **10**.
Figure S49. HRMS spectrum of compound **10**.
Figure S50. ^{1}H NMR spectrum of compound **11**.
Figure S51. ^{13}C NMR spectrum of compound **11**.
Figure S52. HRMS spectrum of compound **11**.
Figure S53. ^{1}H NMR spectrum of compound **12**.
Figure S54. ^{13}C NMR spectrum of compound **12**.
Figure S55. HRMS spectrum of compound **12**.
Figure S56. ^{1}H NMR spectrum of compound **13**.
Figure S57. ^{13}C NMR spectrum of compound **13**.
Figure S58. HRMS spectrum of compound **13**.
Figure S59. ^{1}H NMR spectrum of compound **14**.
Figure S60. ^{13}C NMR spectrum of compound **14**.
Figure S61. HRMS spectrum of compound **14**.
Figure S62. ^{1}H NMR spectrum of compound **15**.
Figure S63. ^{13}C NMR spectrum of compound **15**.
Figure S64. HRMS spectrum of compound **15**.
Figure S65. ^{1}H NMR spectrum of compound **16**.
Figure S66. ^{13}C NMR spectrum of compound **16**.
Figure S67. HRMS spectrum of compound **16**.
Figure S68. ^{1}H NMR spectrum of compound **17**.
Figure S69. ^{13}C NMR spectrum of compound **17**.
Figure S70. HRMS spectrum of compound **17**.
Figure S71. ^{1}H NMR spectrum of compound **18**.
Figure S72. ^{13}C NMR spectrum of compound **18**.
Figure S73. HRMS spectrum of compound **18**.
Figure S74. ^{1}H NMR spectrum of compound **19**.
Figure S75. ^{13}C NMR spectrum of compound **19**.
Figure S76. HRMS spectrum of compound **19**.
Figure S77. ^{1}H NMR spectrum of compound **20**.
Figure S78. ^{13}C NMR spectrum of compound **20**.
Figure S79. HRMS spectrum of compound **20**.

Figure S1. ^{1}H NMR spectrum of compound **1**.

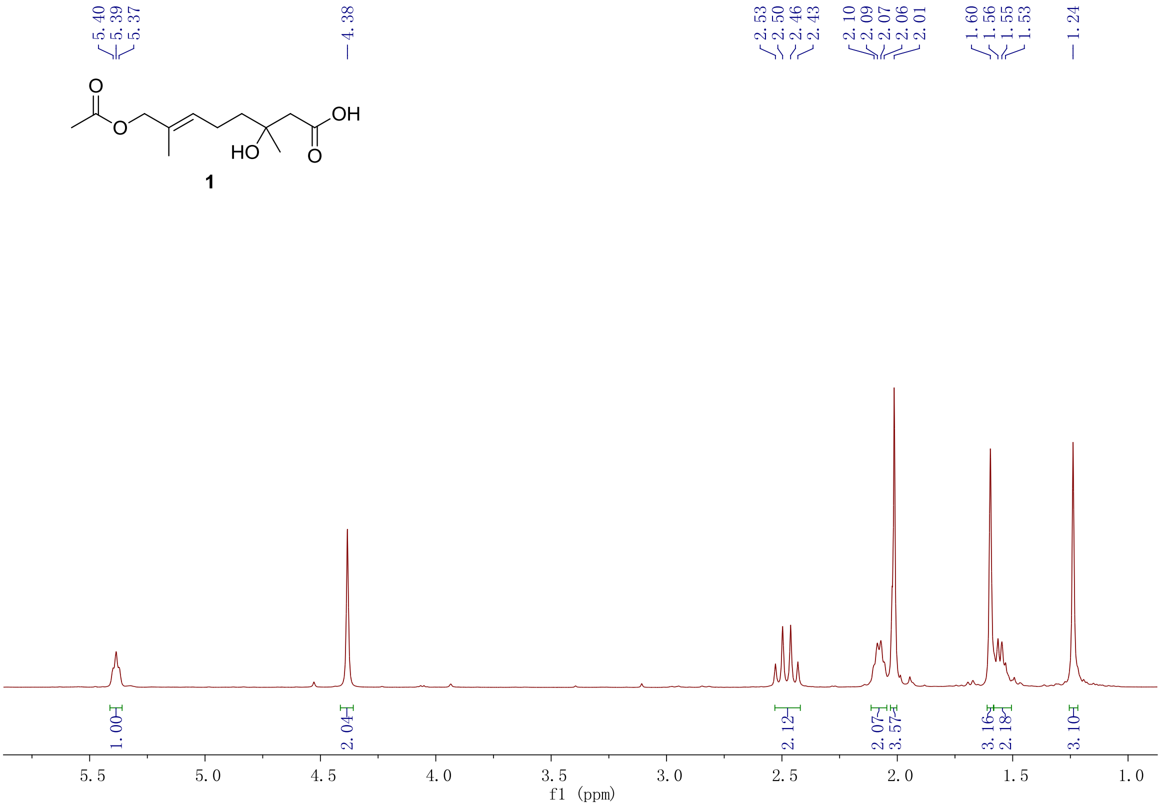

Figure S2. ^{13}C NMR spectrum of compound **1**.

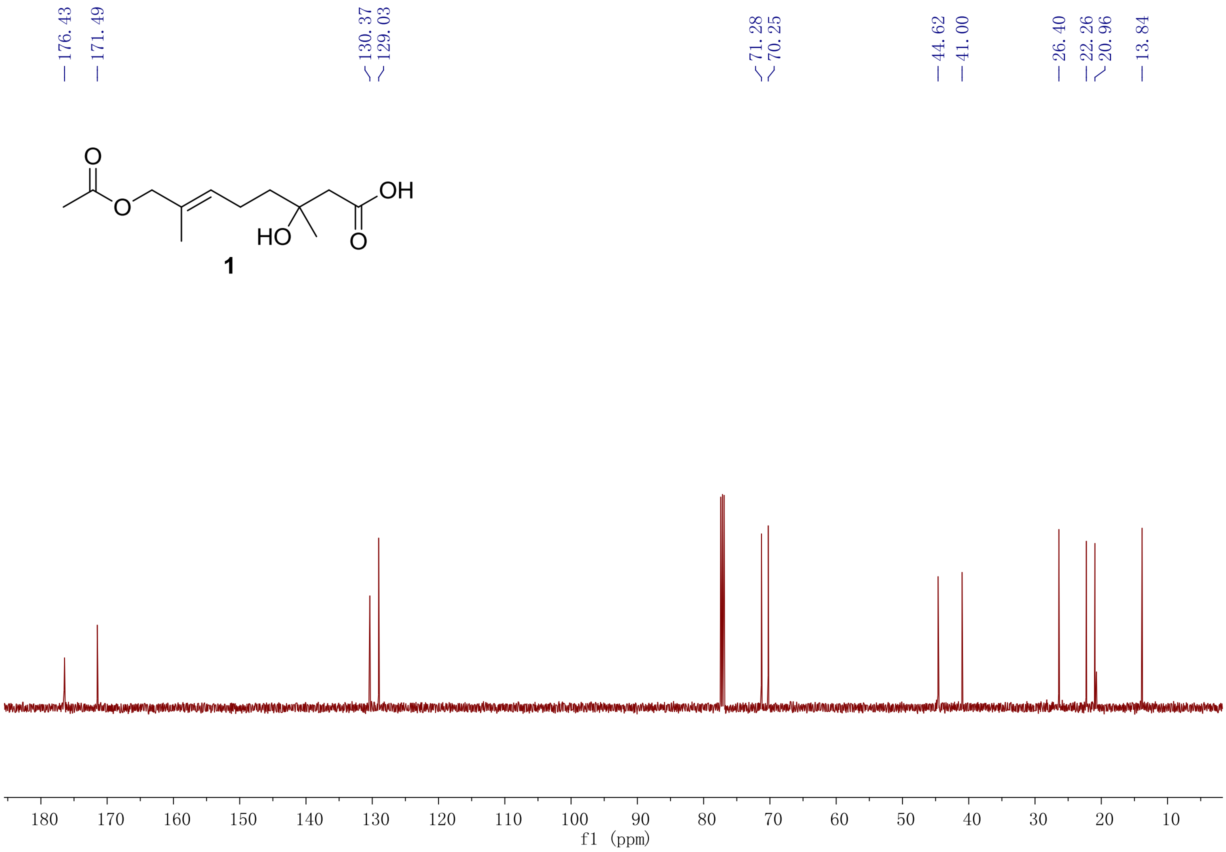

Figure S3. HRMS of compound **1**.

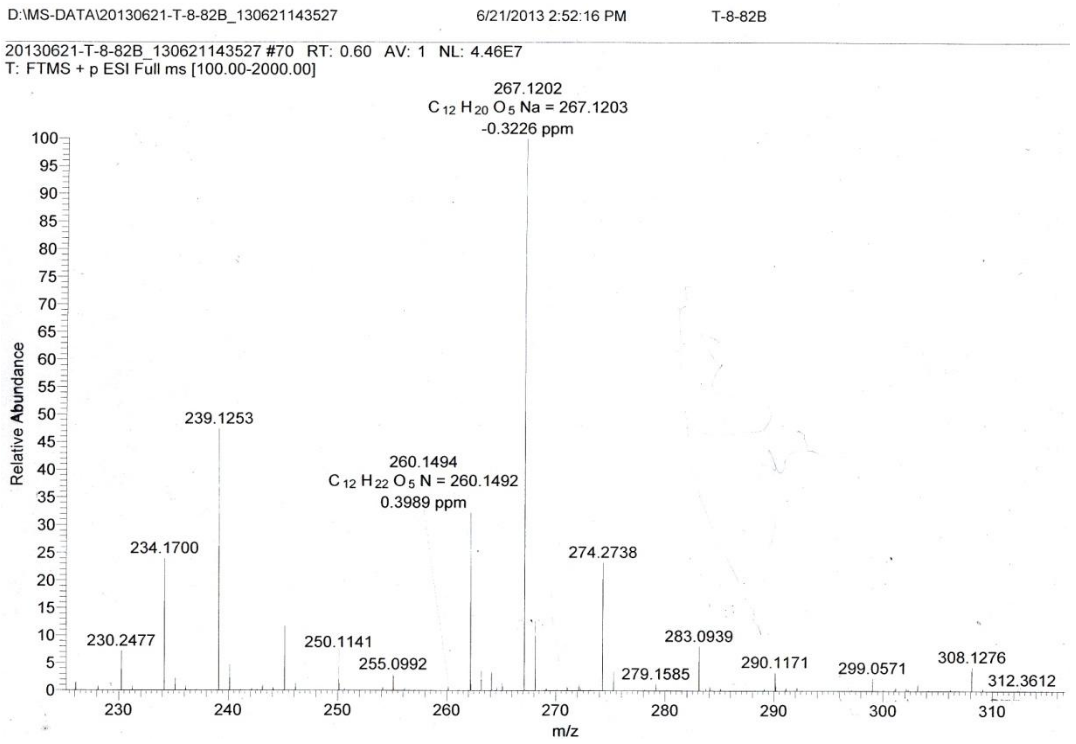

Figure S4. ^{1}H NMR spectrum of compound **2a**.

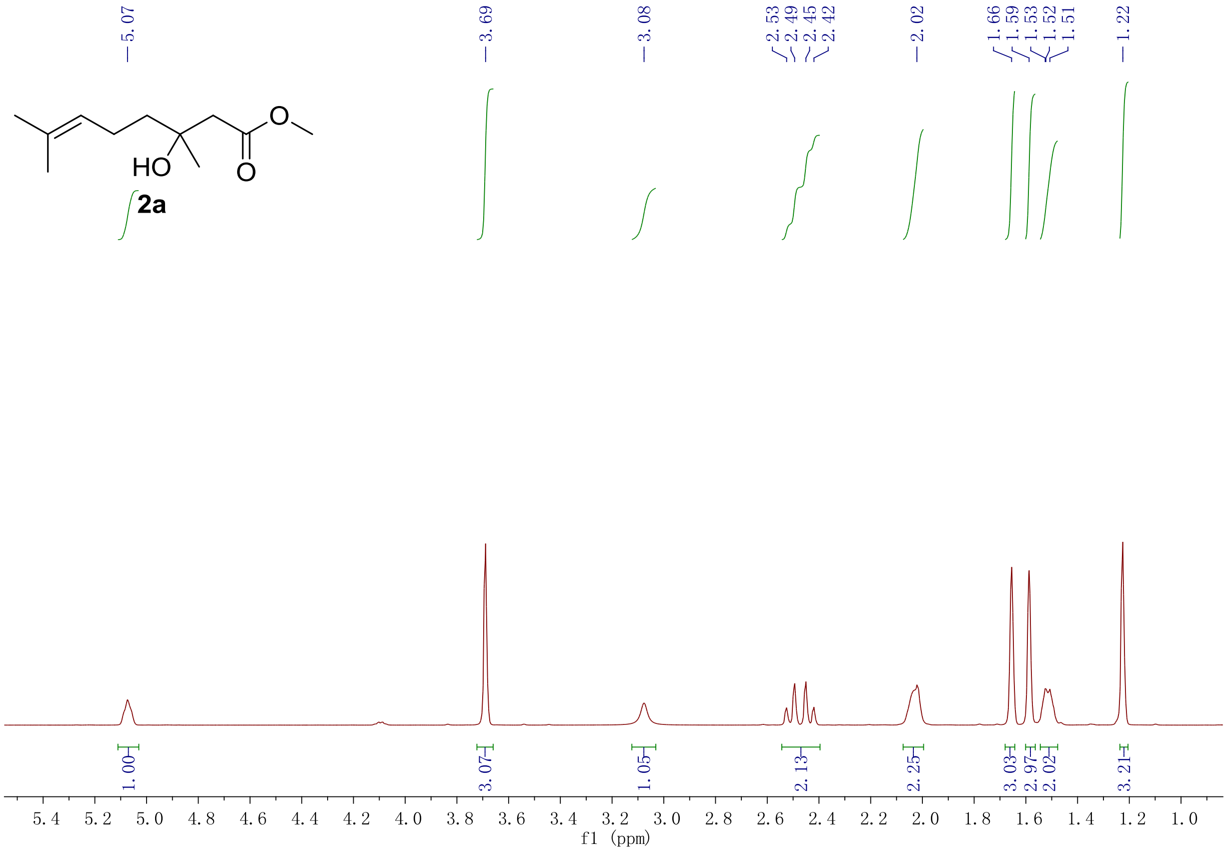

Figure S5. ^{13}C NMR spectrum of compound **2a**.

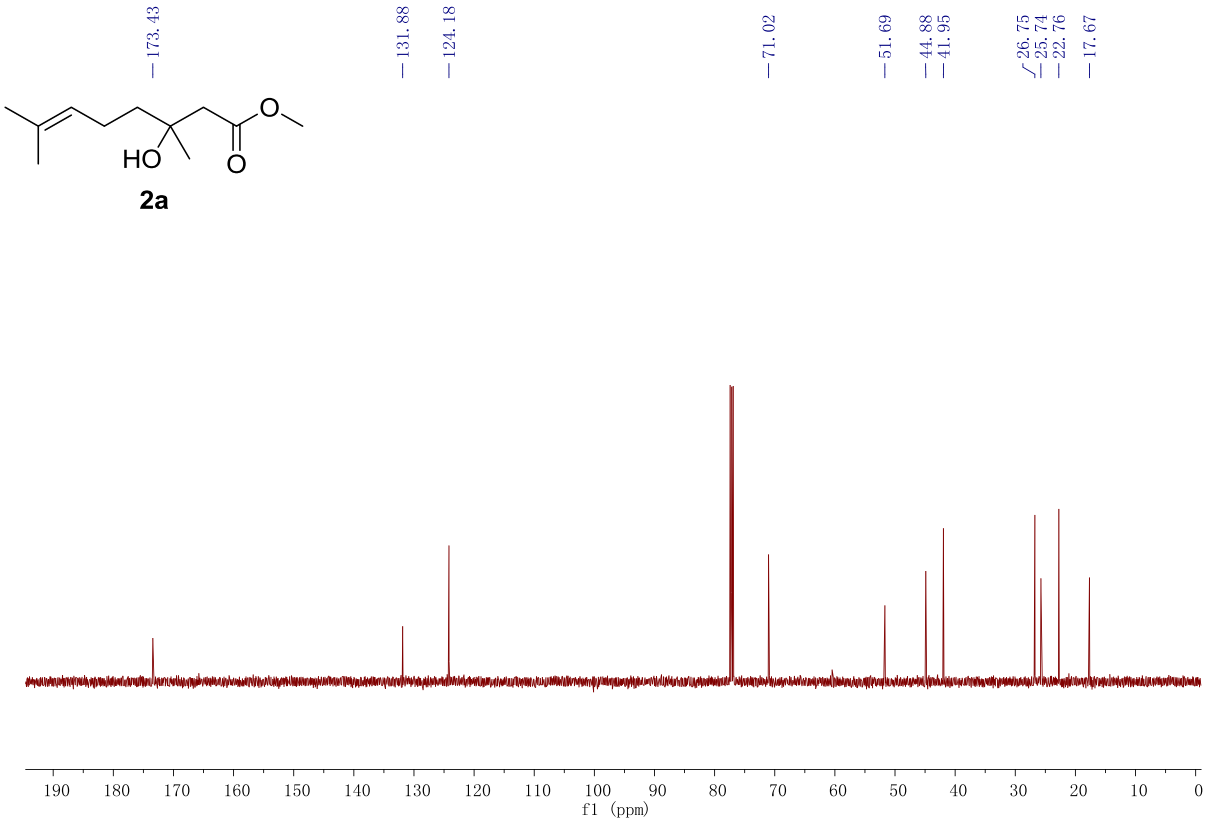

Figure S6. HRMS of compound **2a**.

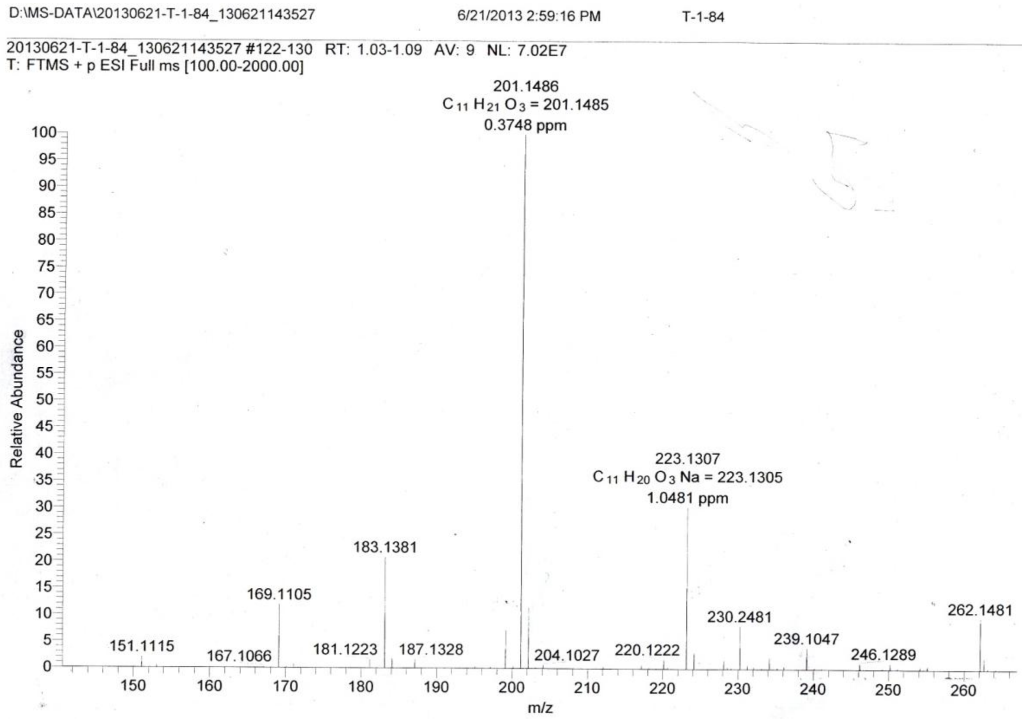

Figure S7. ^{1}H NMR spectrum of compound 2b.

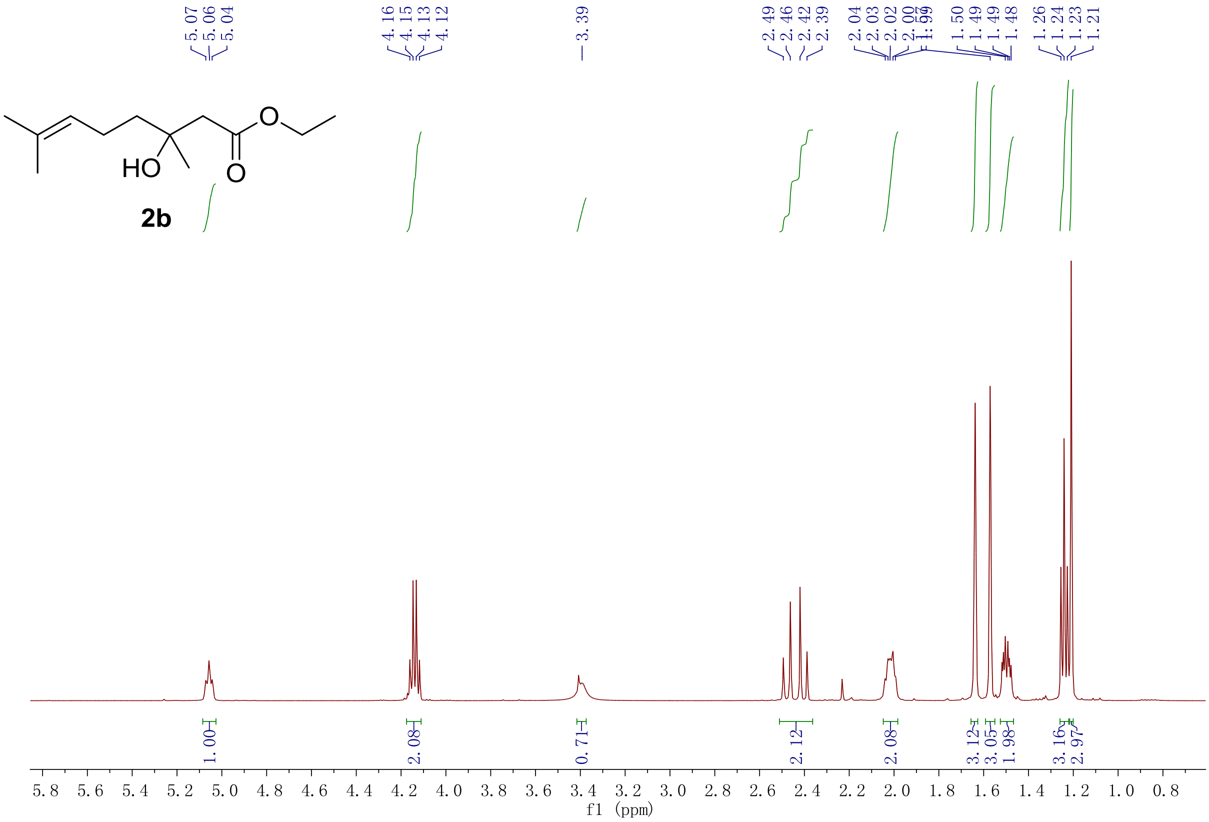

Figure S8. ^{13}C NMR spectrum of compound **2b**.

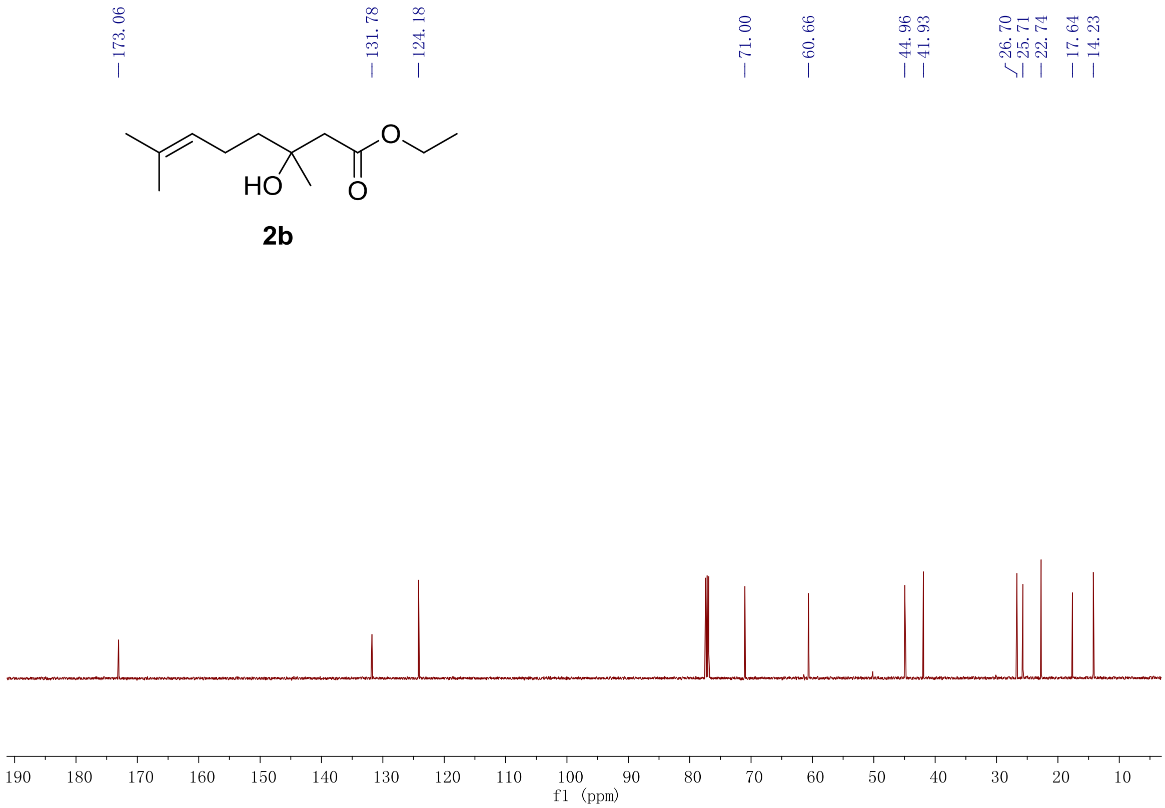

Figure S9. HRMS of compound **2b**.

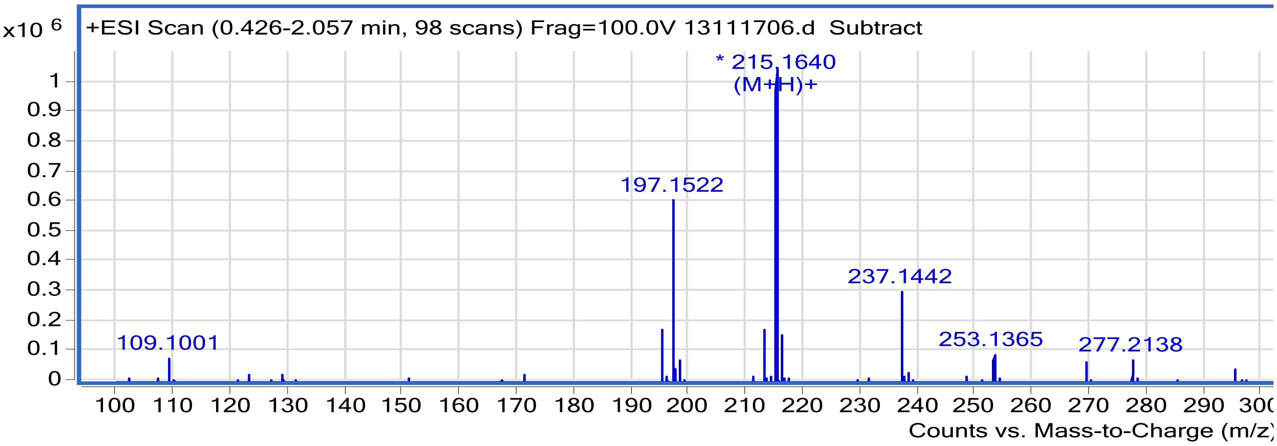

Figure S10. ^{1}H NMR spectrum of compound **3a**.

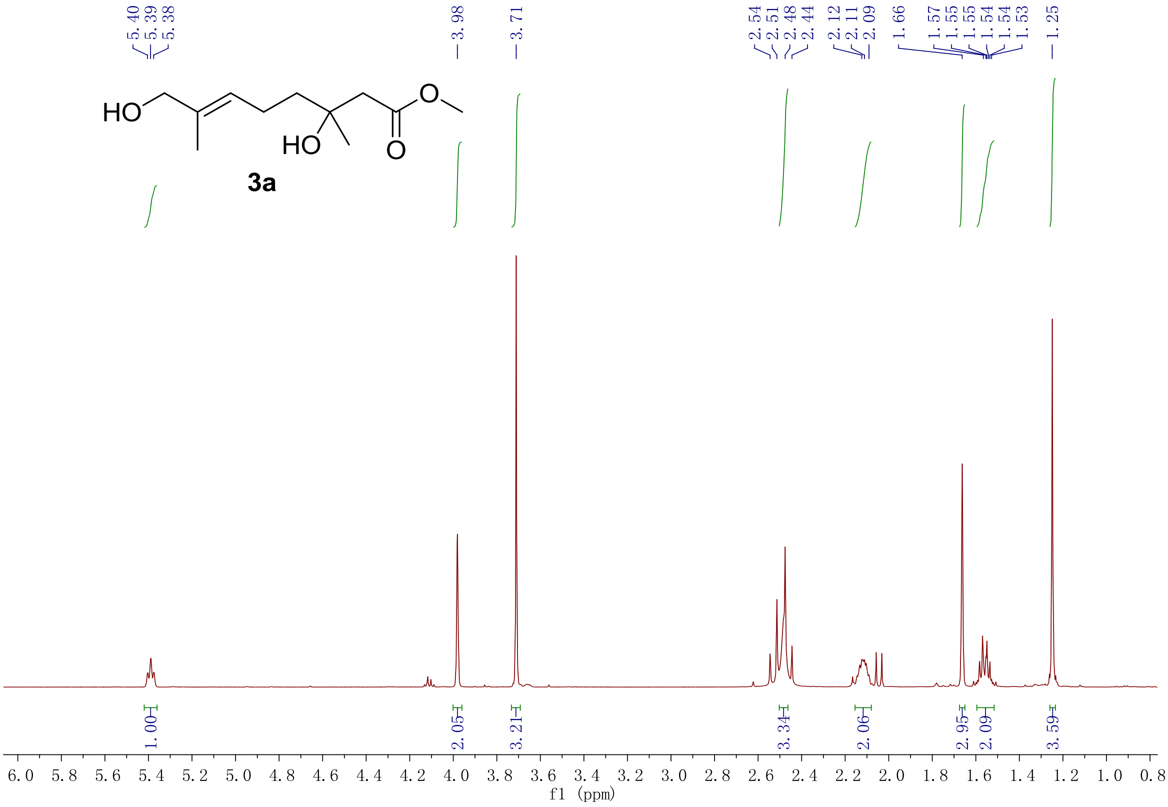

Figure S11. ^{13}C NMR spectrum of compound 3a.

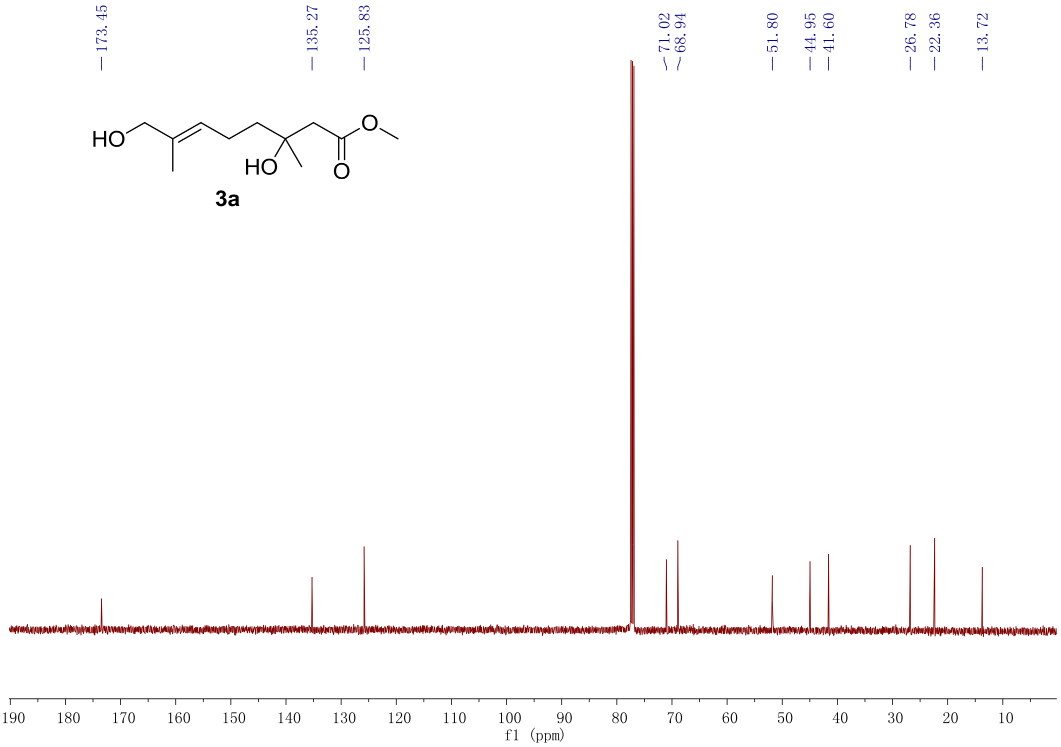

Figure S12. NOESY spectrum of compound **3a**.

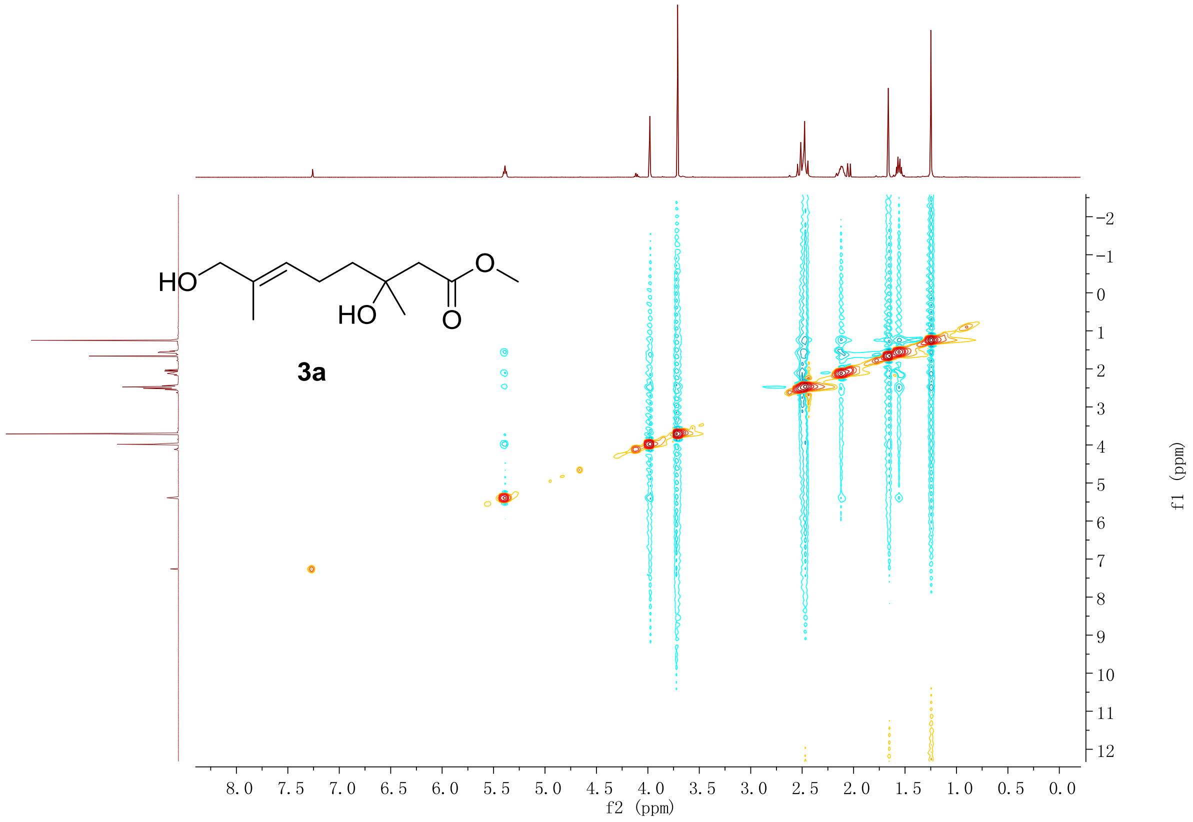

Figure S13. An enlarged area of the NOESY spectrum of compound **3a**.

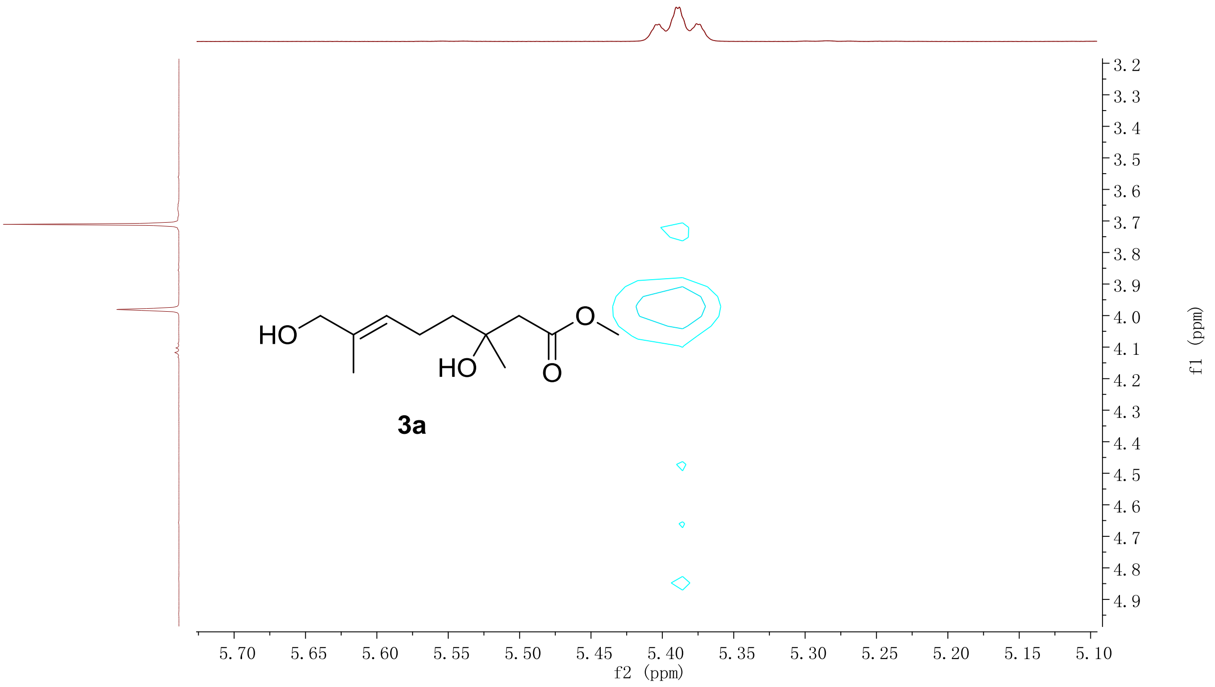

Figure S14. HRMS of compound **3a**.

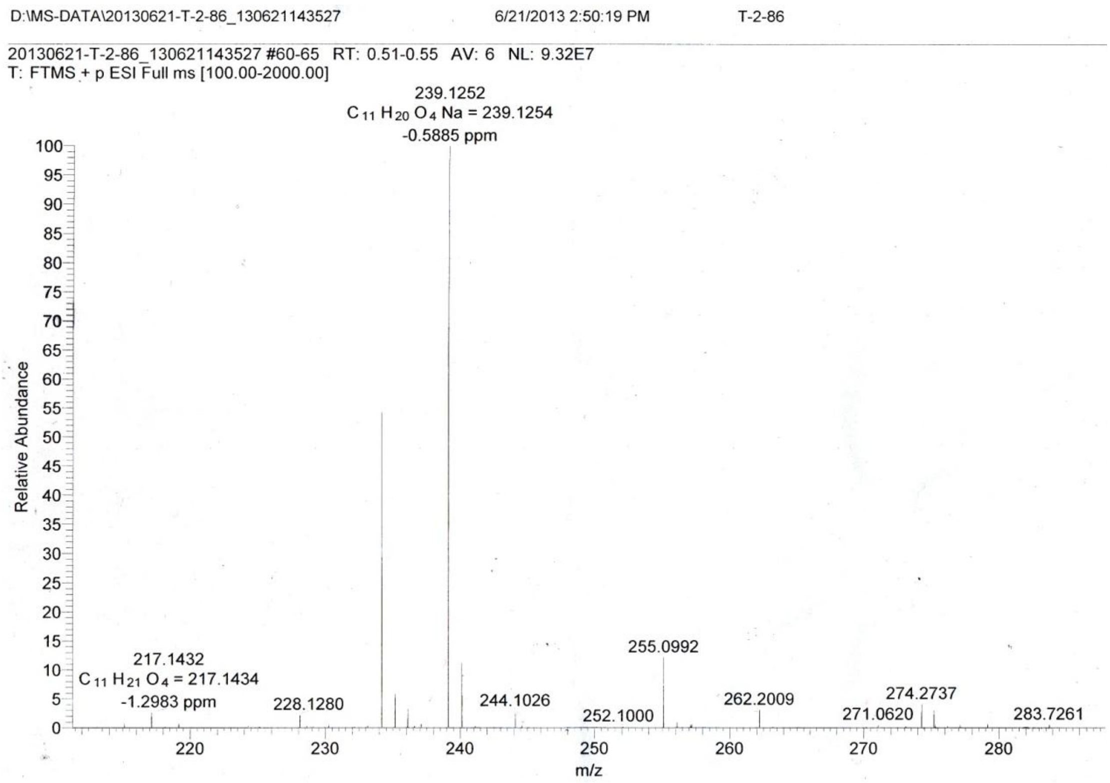

Figure S15. ^{1}H NMR spectrum of compound **3b**.

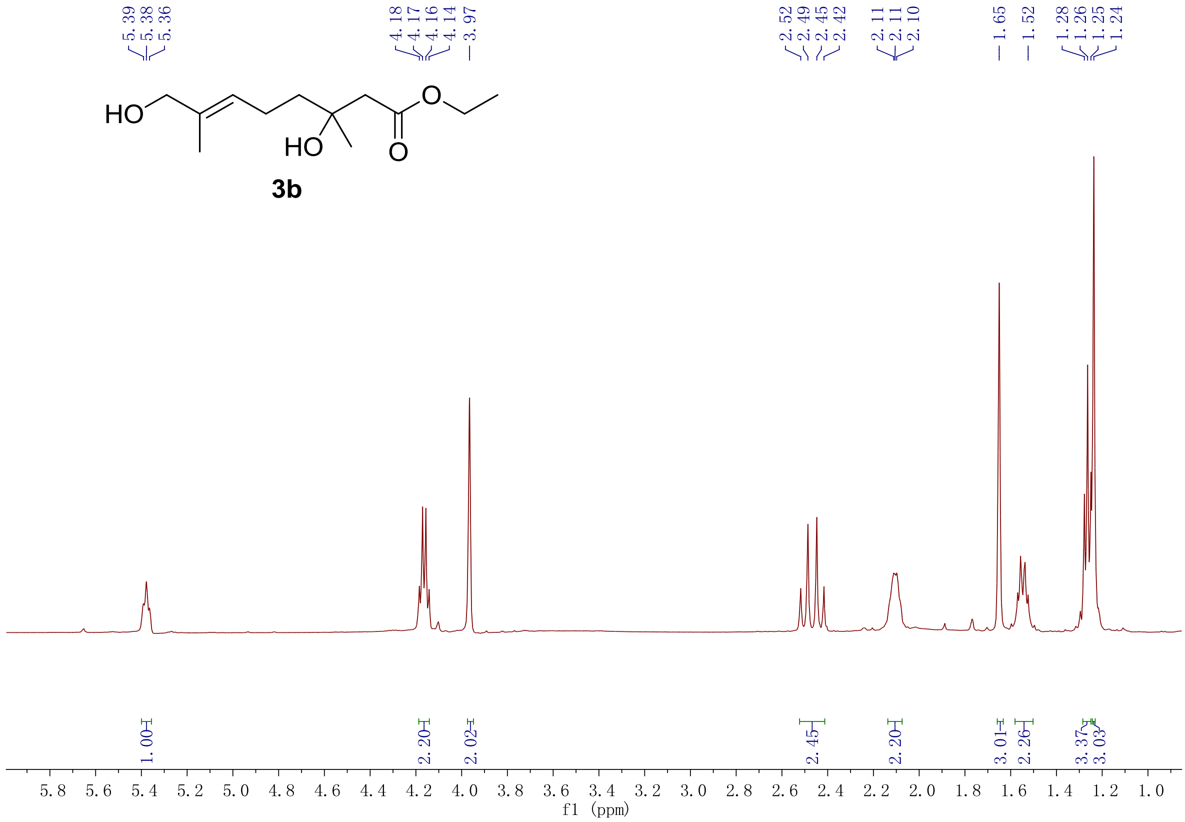

Figure S16. ^{13}C NMR spectrum of compound **3b**.

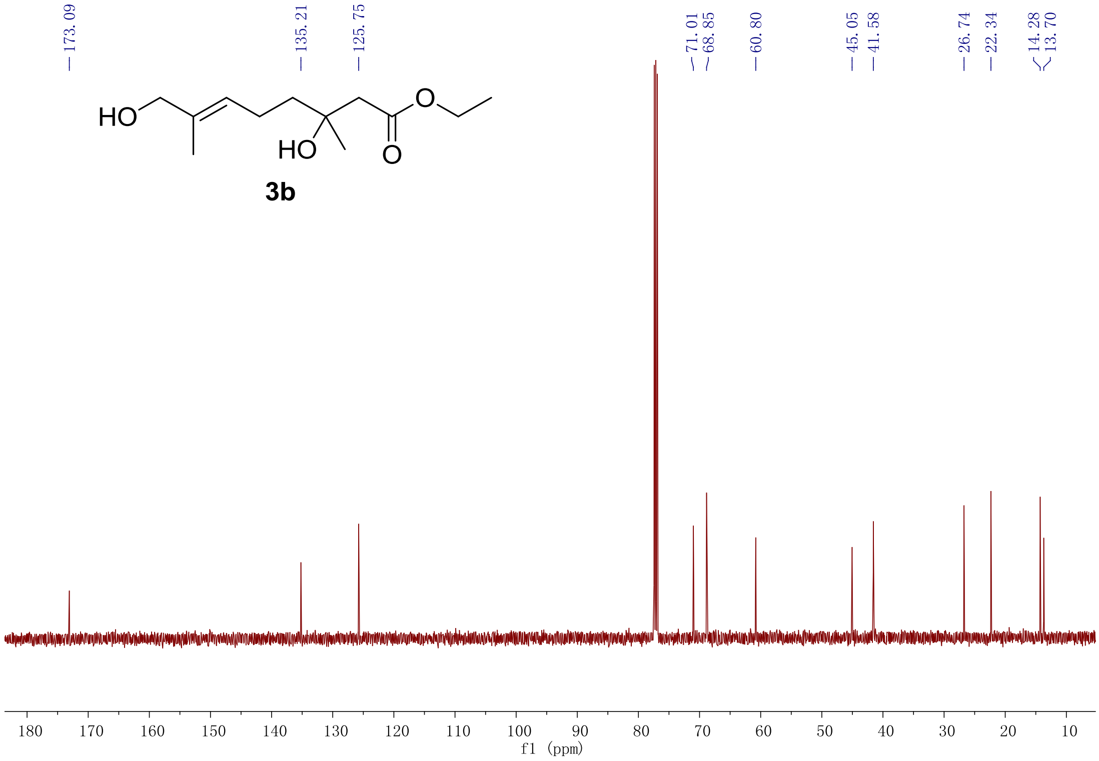

Figure S17. HRMS of compound **3b**.

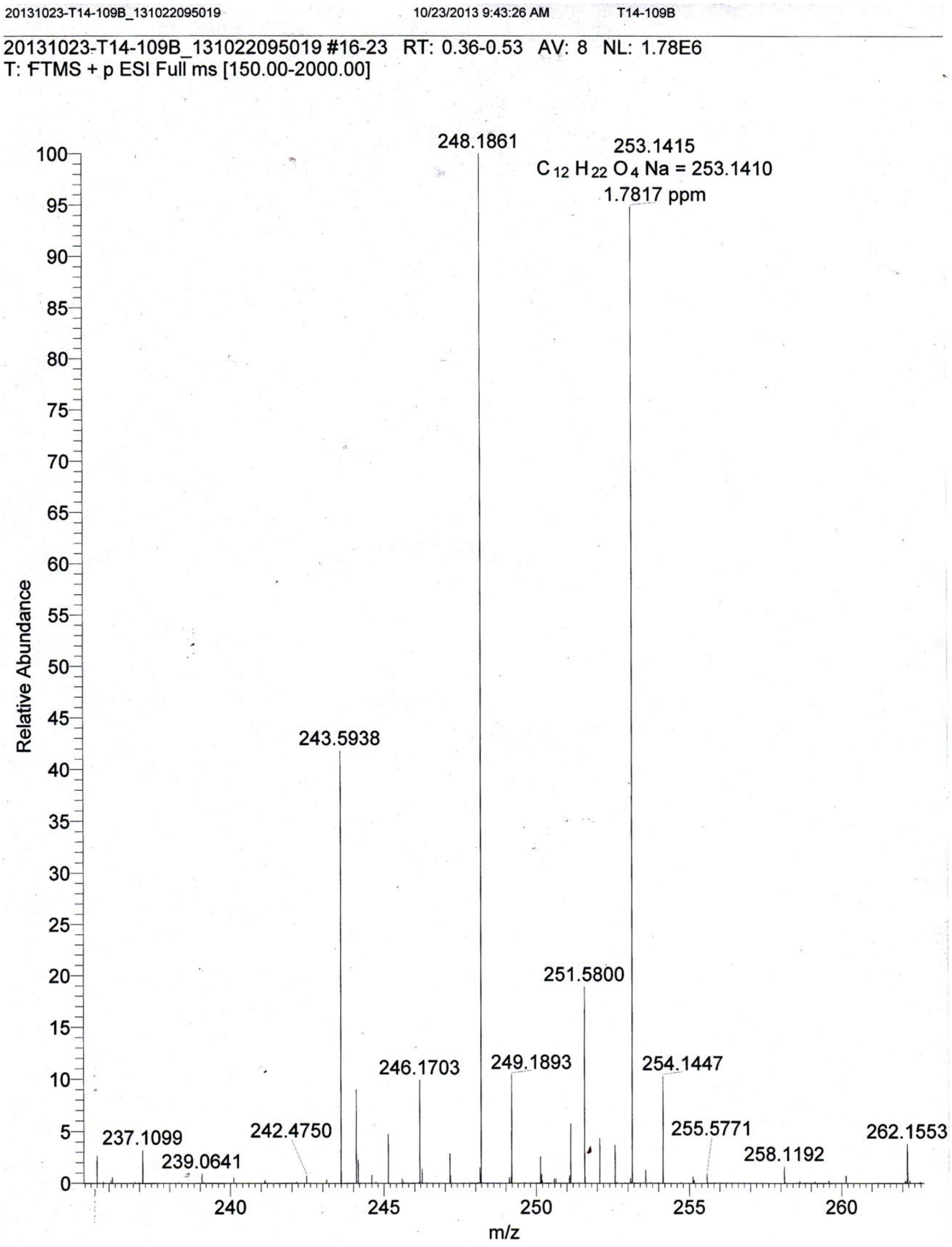

Figure S18. ^{1}H NMR spectrum of compound **4a**.

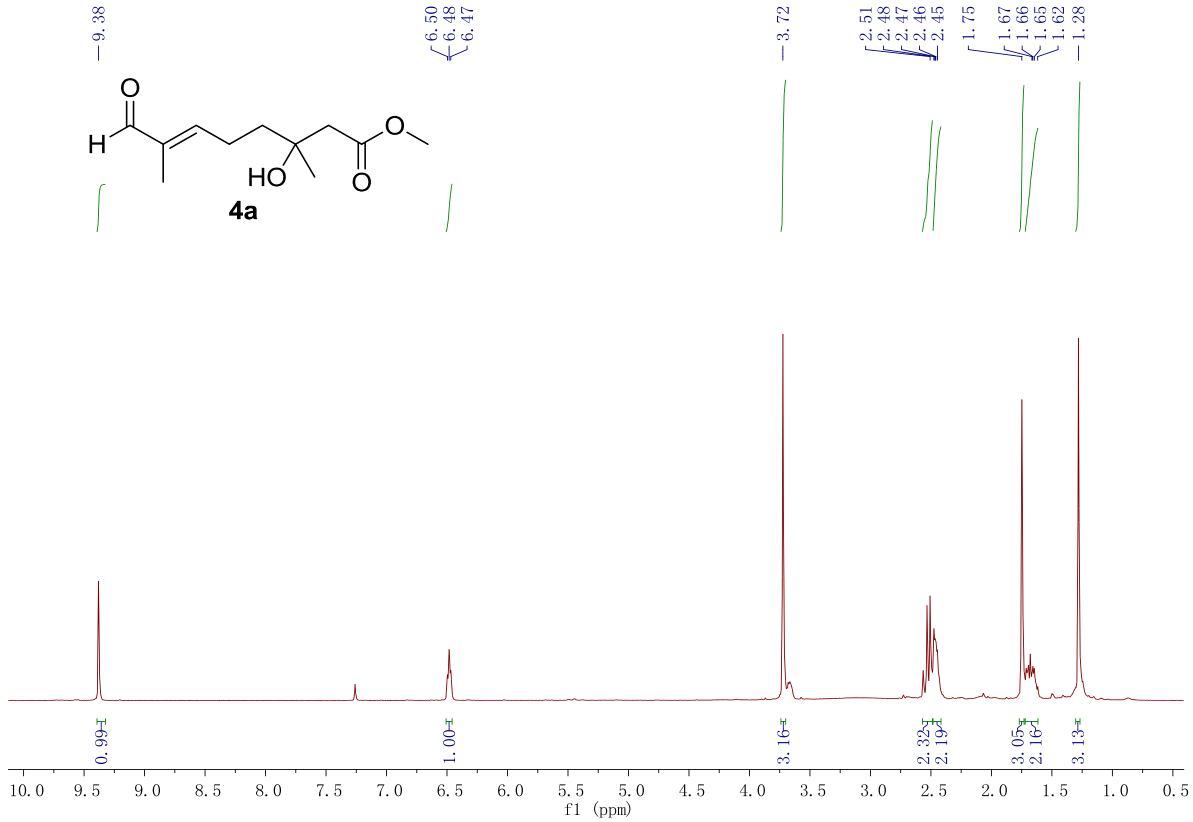

Figure S19. ^{13}C NMR spectrum of compound 4a.

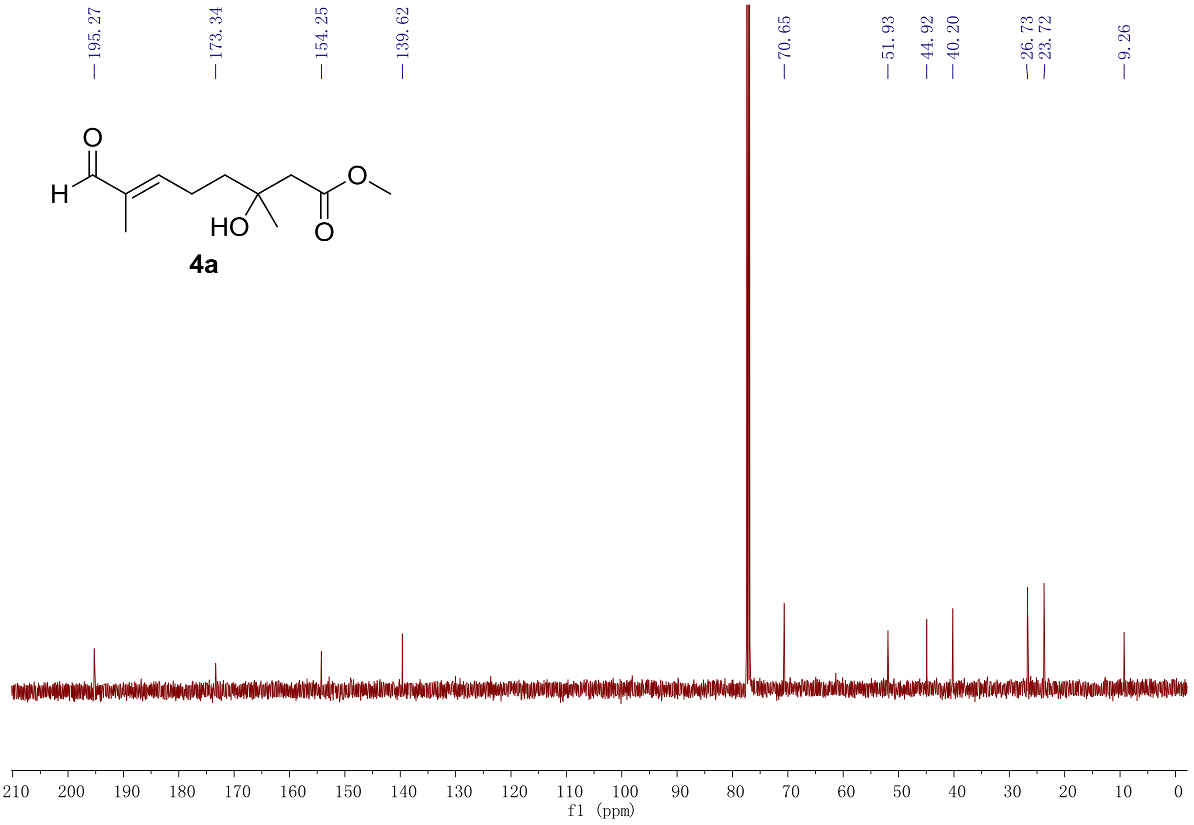

Figure S20. HRMS of compound **4a**.

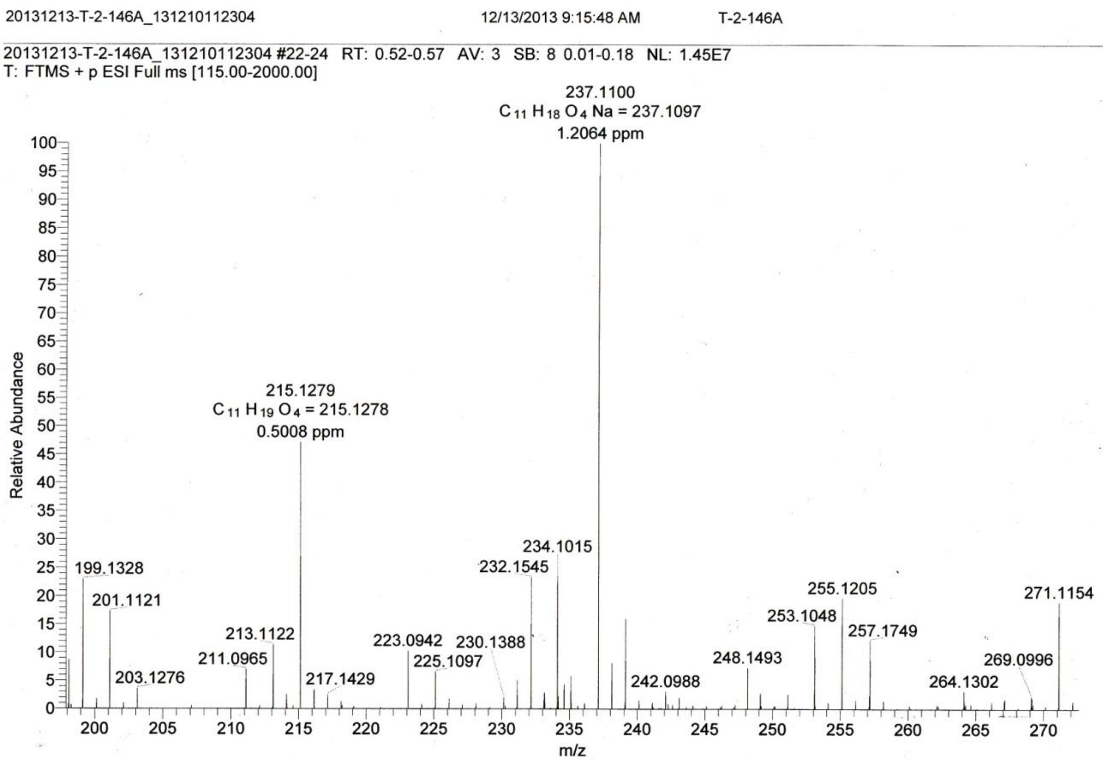

Figure S21. ^{1}H NMR spectrum of compound **4b**.

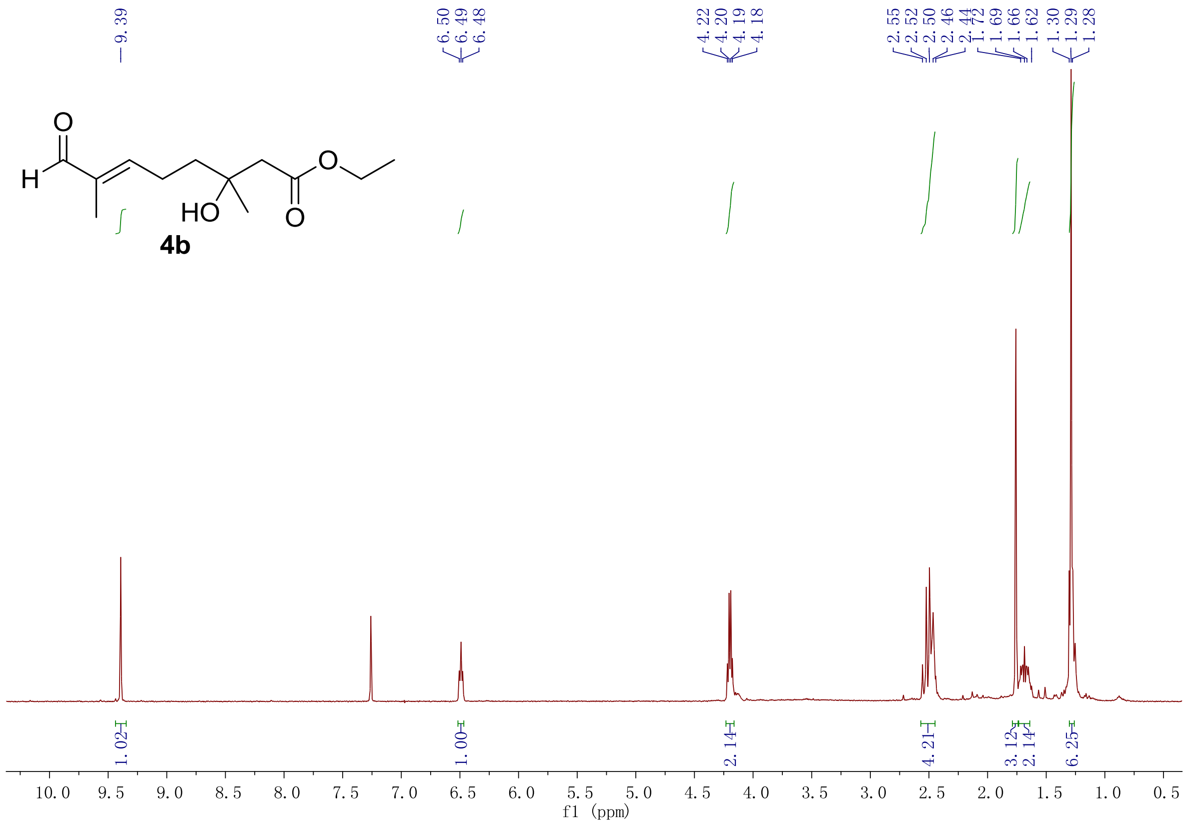

Figure S22. ^{13}C NMR spectrum of compound **4b**.

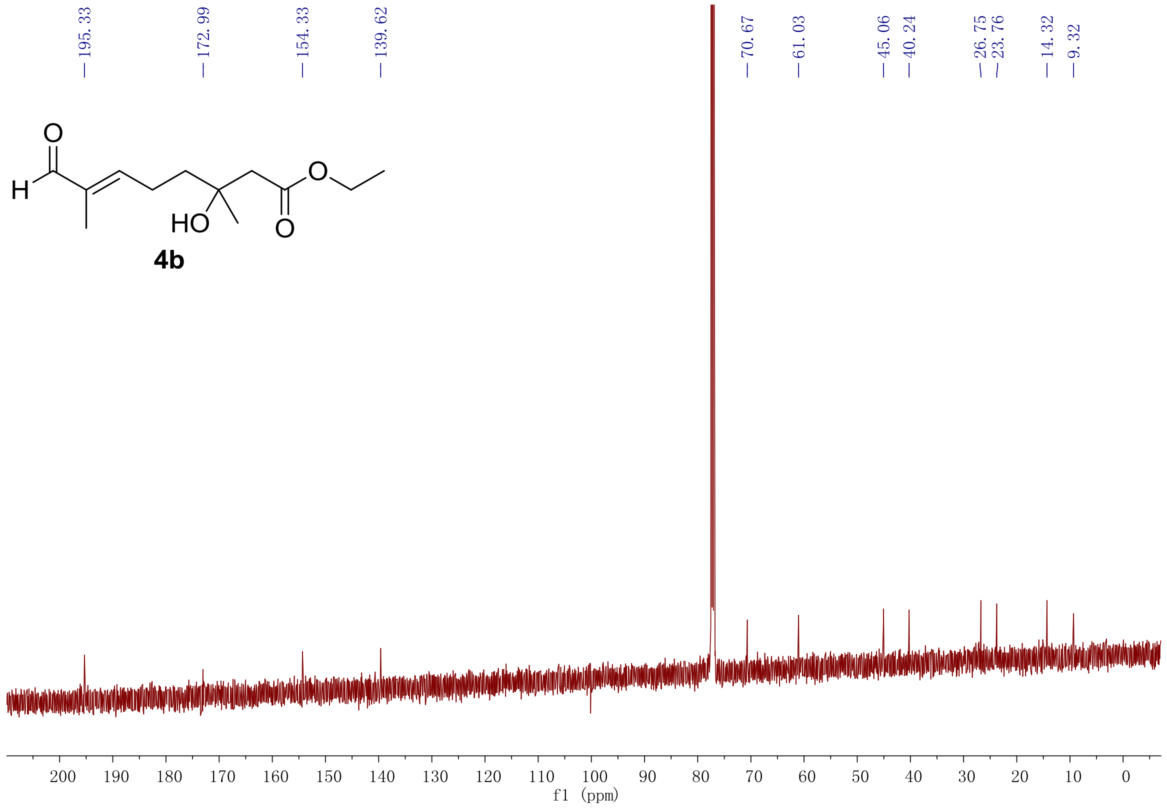

Figure S23. HRMS of compound **4b**.

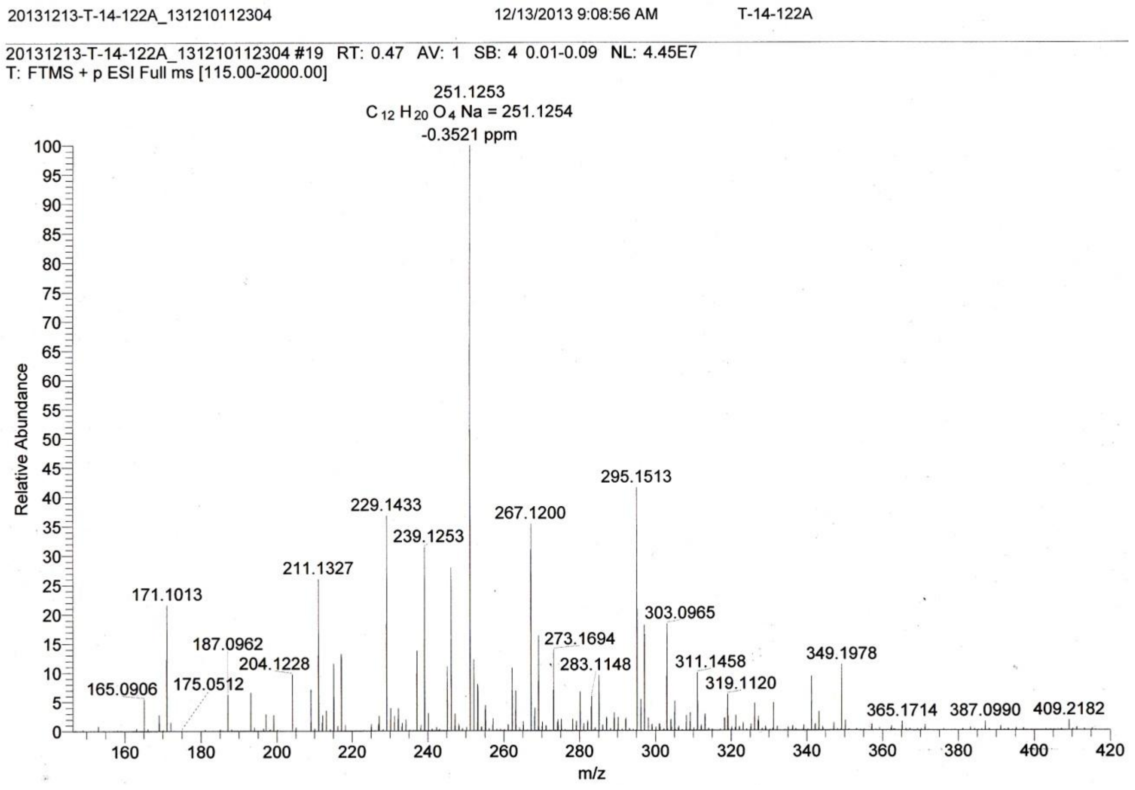

Figure S24. ^{1}H NMR spectrum of compound **5**.

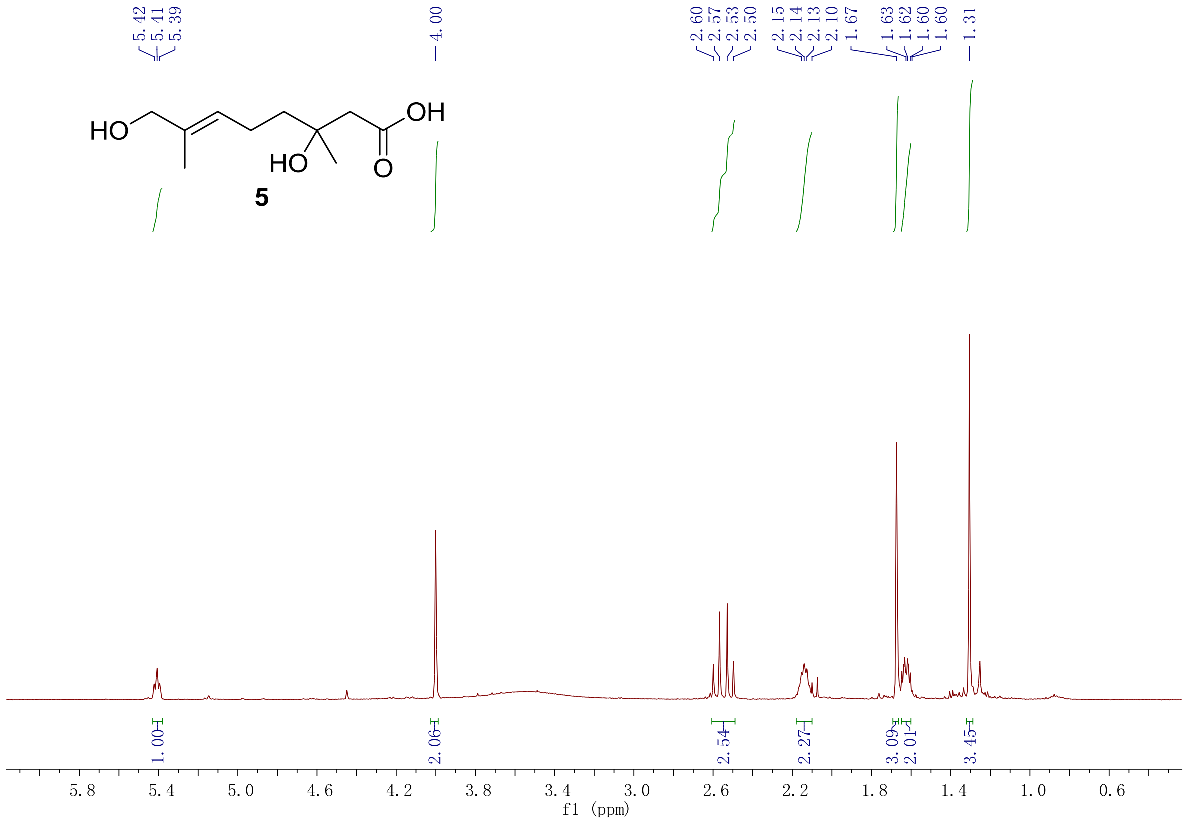

Figure S25. ^{13}C NMR spectrum of compound **5**.

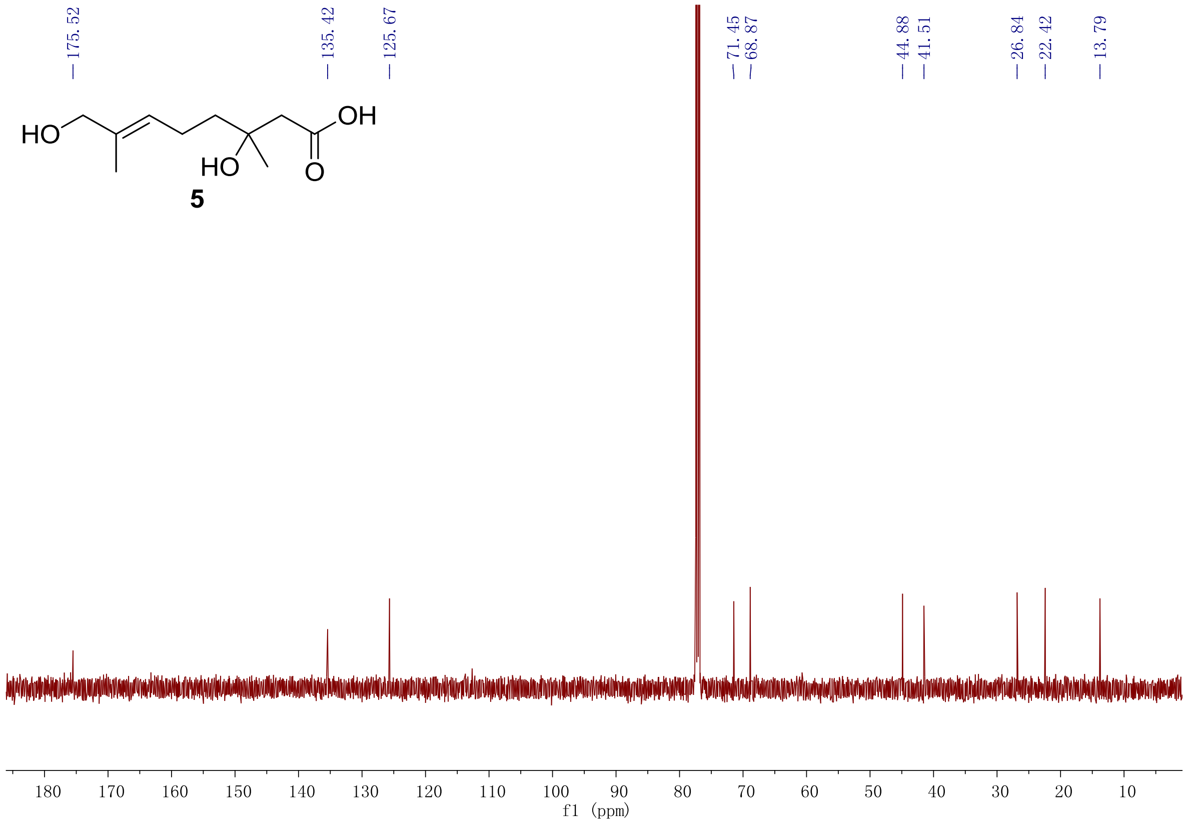

Figure S26. HRMS of compound **5**.

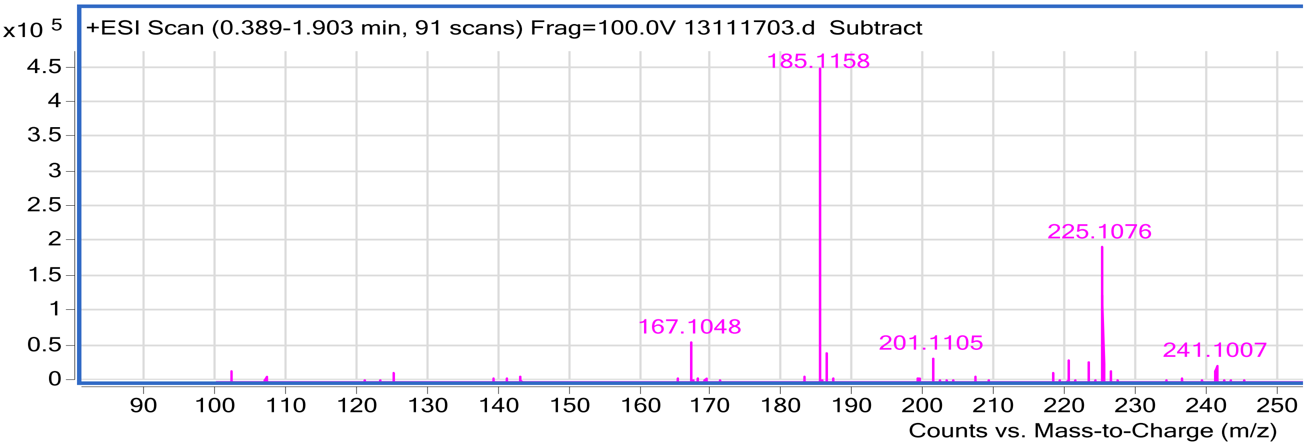

Figure S27. ^{1}H NMR spectrum of compound **6**.

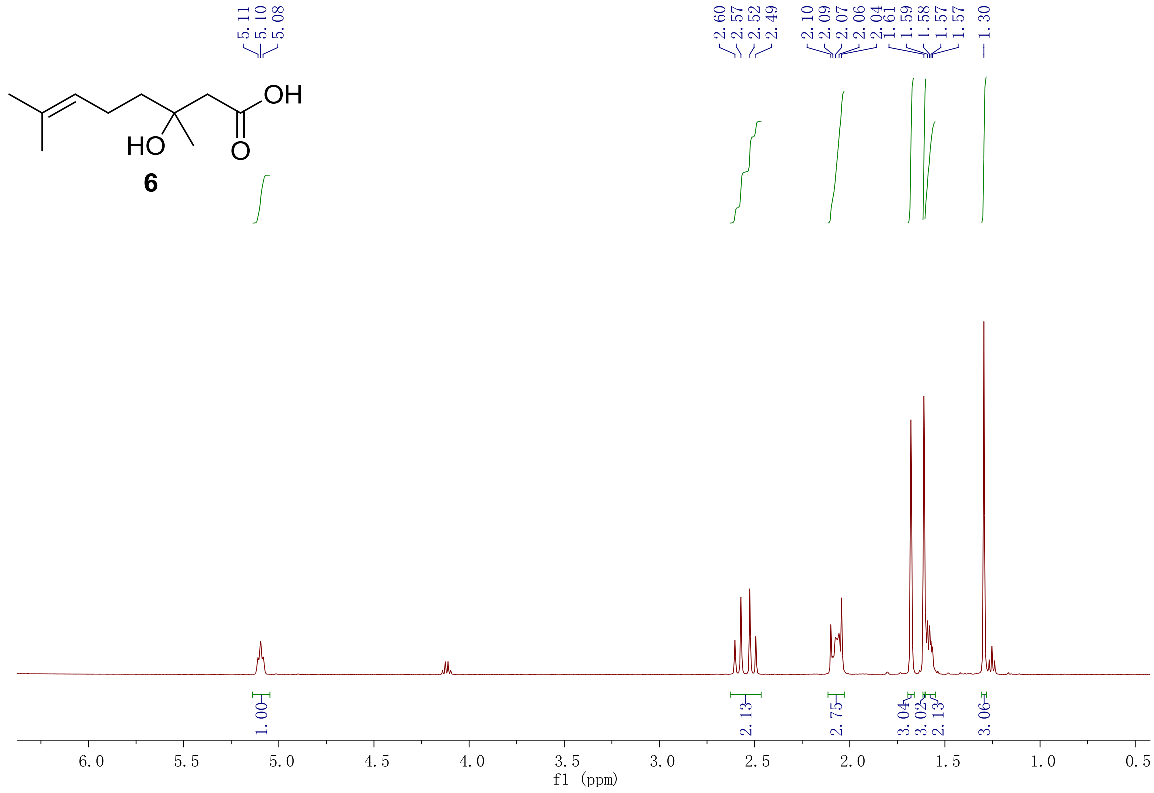

Figure S28. ^{13}C NMR spectrum of compound **6**.

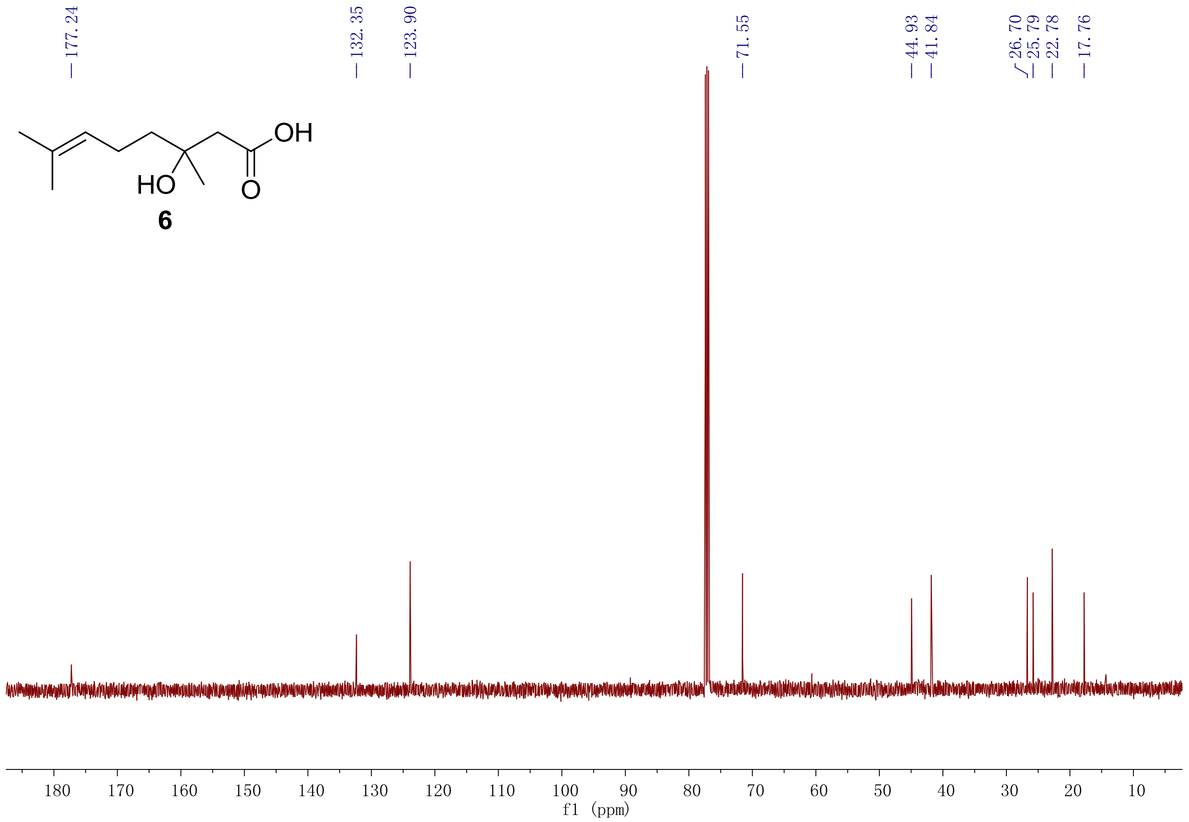

Figure S29. HRMS of compound **6**.

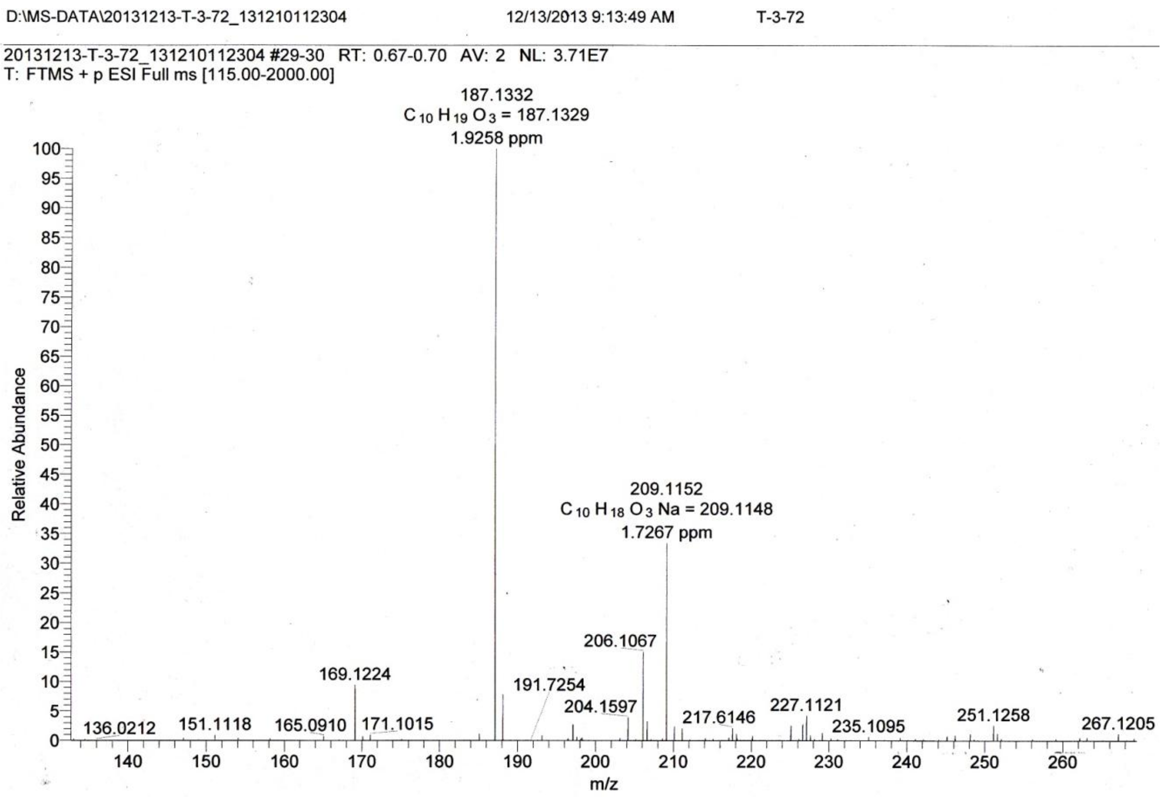

Figure S30. ^{1}H NMR spectrum of compound **7**.

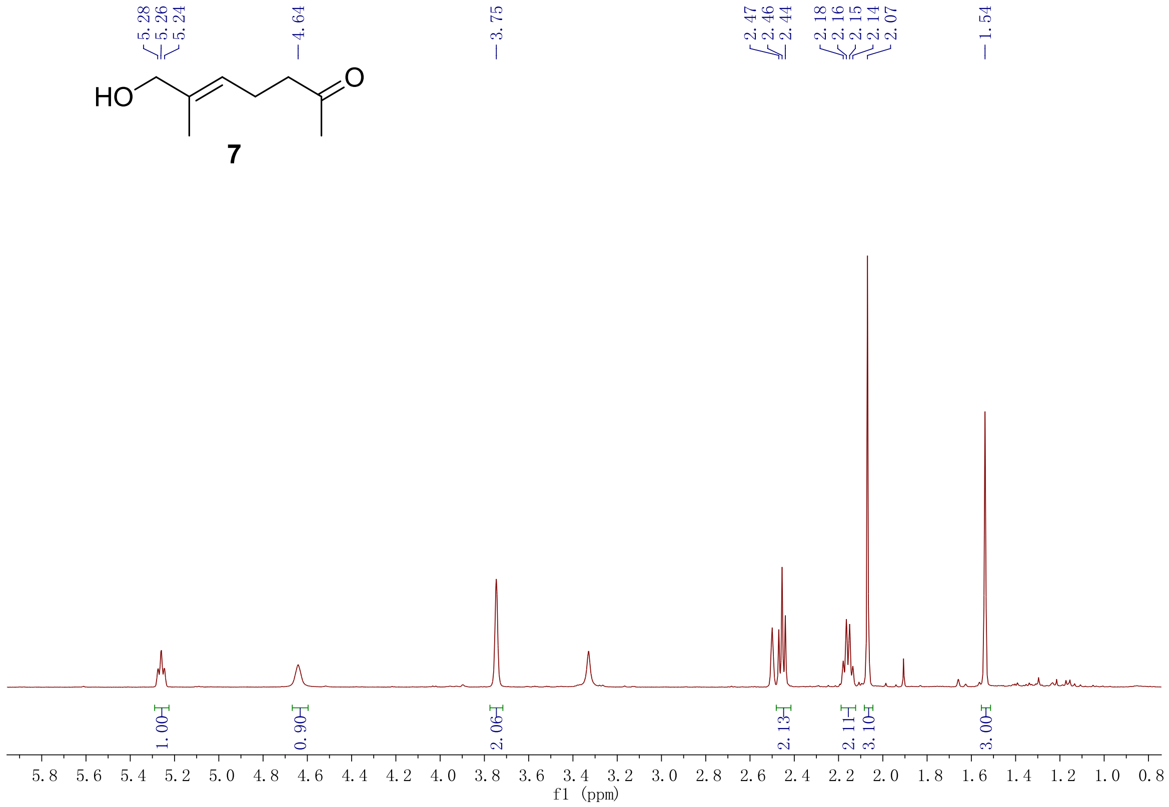

Figure S31. ^{13}C NMR spectrum of compound 7.

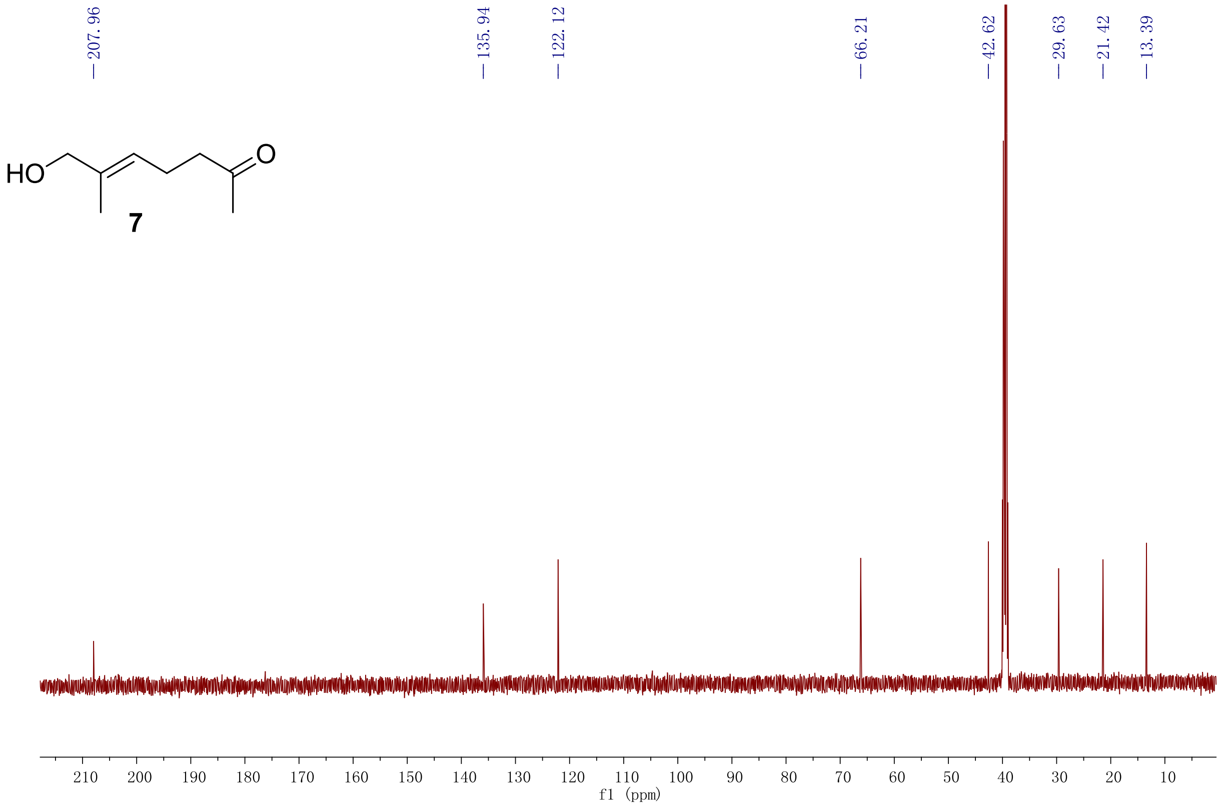

Figure S32. ^{1}H NMR spectrum of compound **8a**.

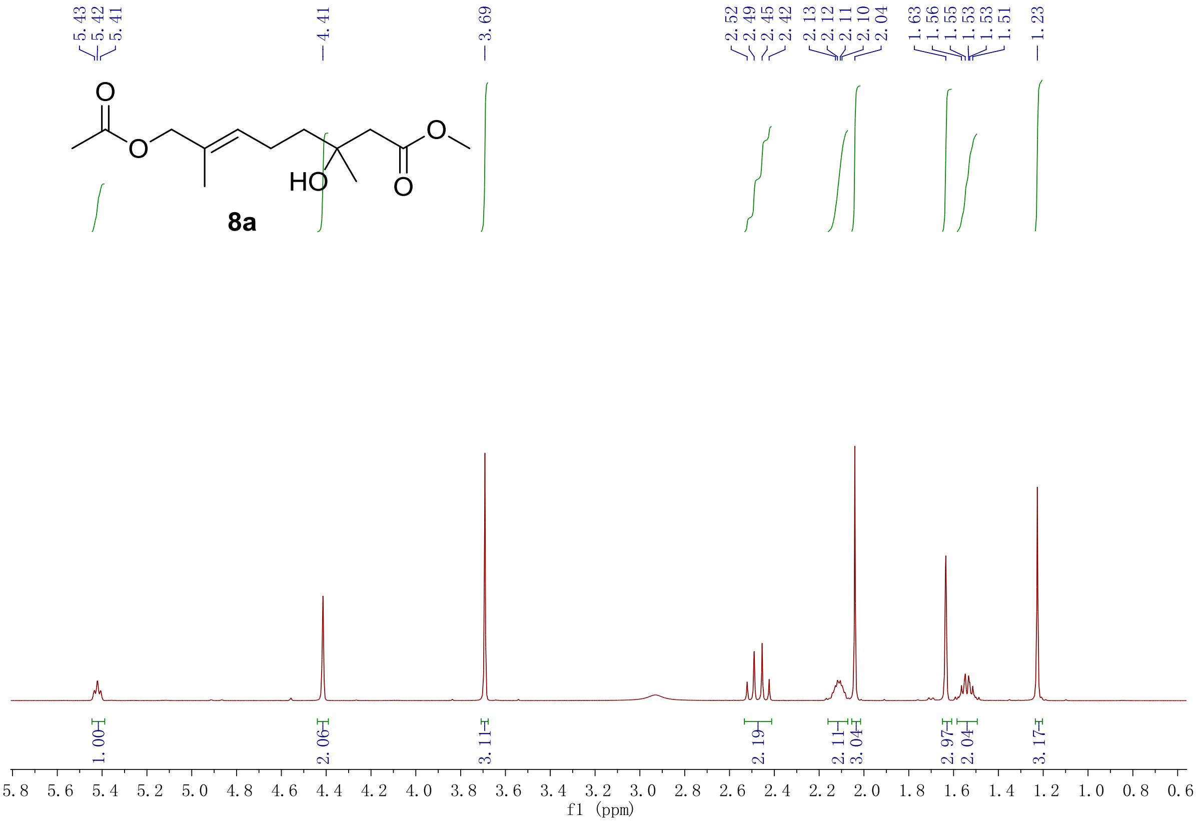

Figure S33. ^{13}C NMR spectrum of compound **8a**.

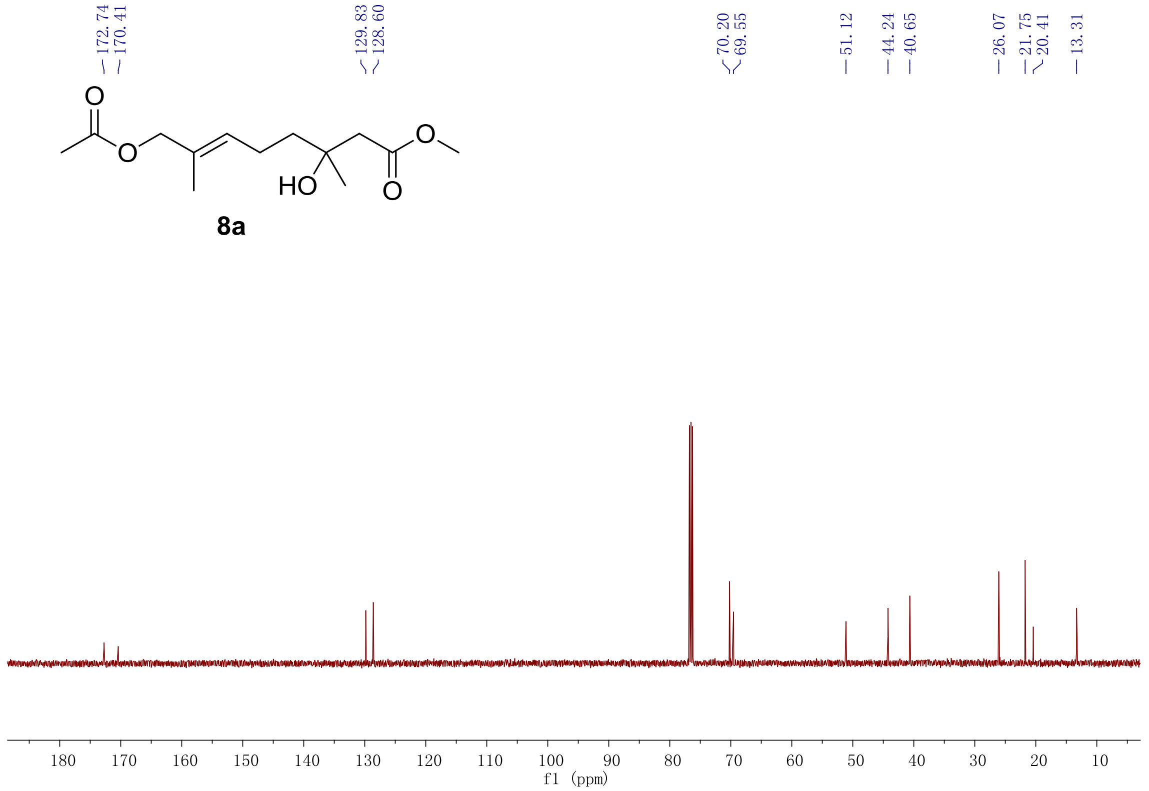

Figure S34. HRMS of compound **8a**.

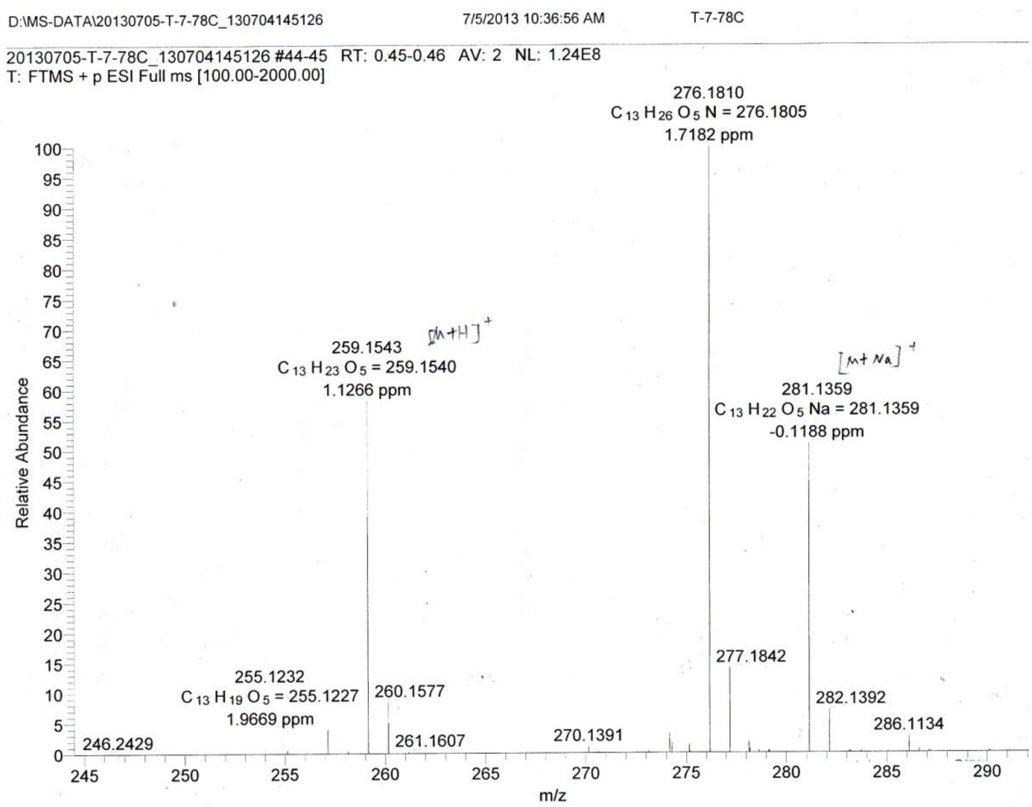

Figure S35. ^{1}H NMR spectrum of compound 8b.

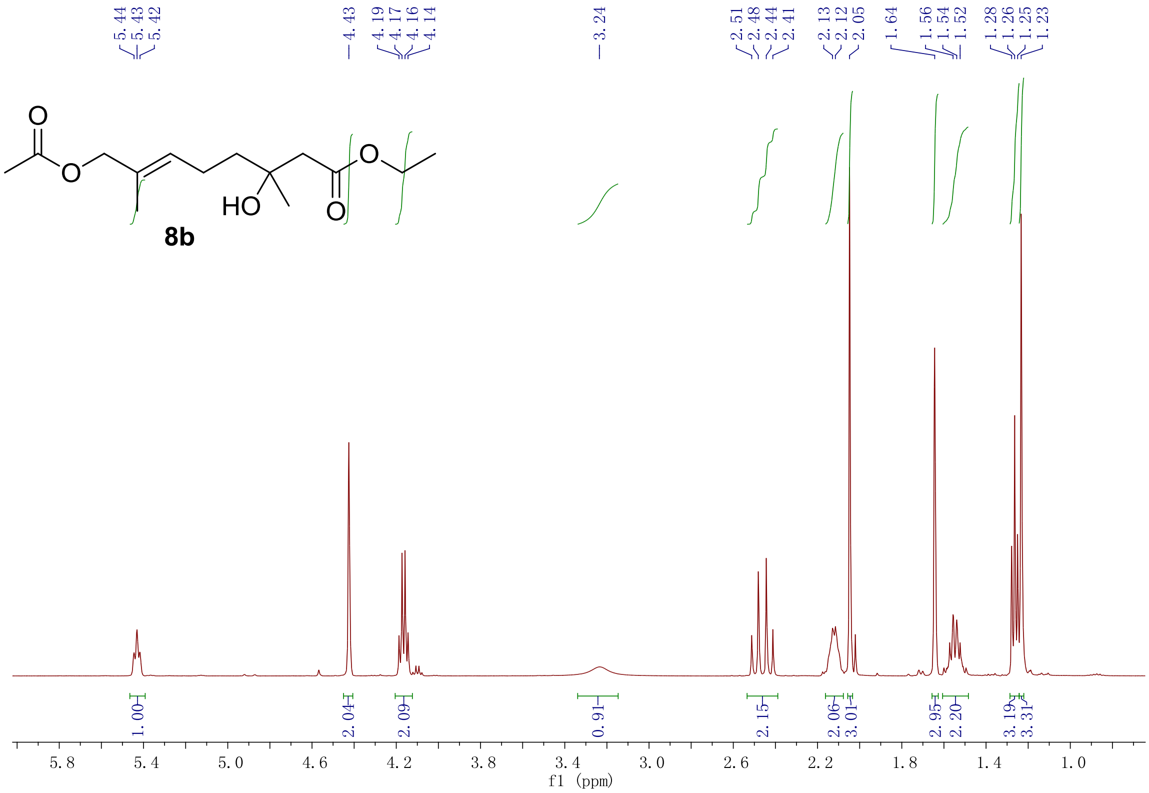

Figure S36. ^{13}C NMR spectrum of compound **8b**.

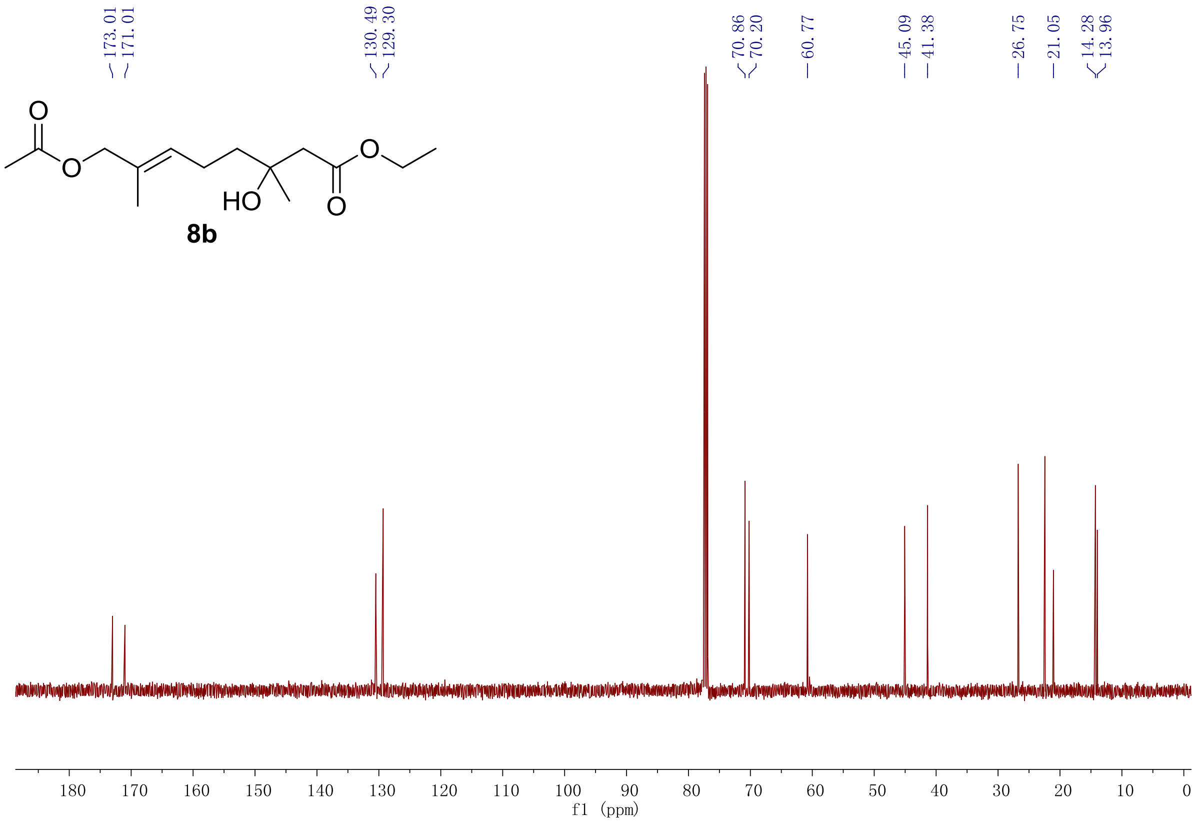

Figure S37. HRMS of compound 8b.

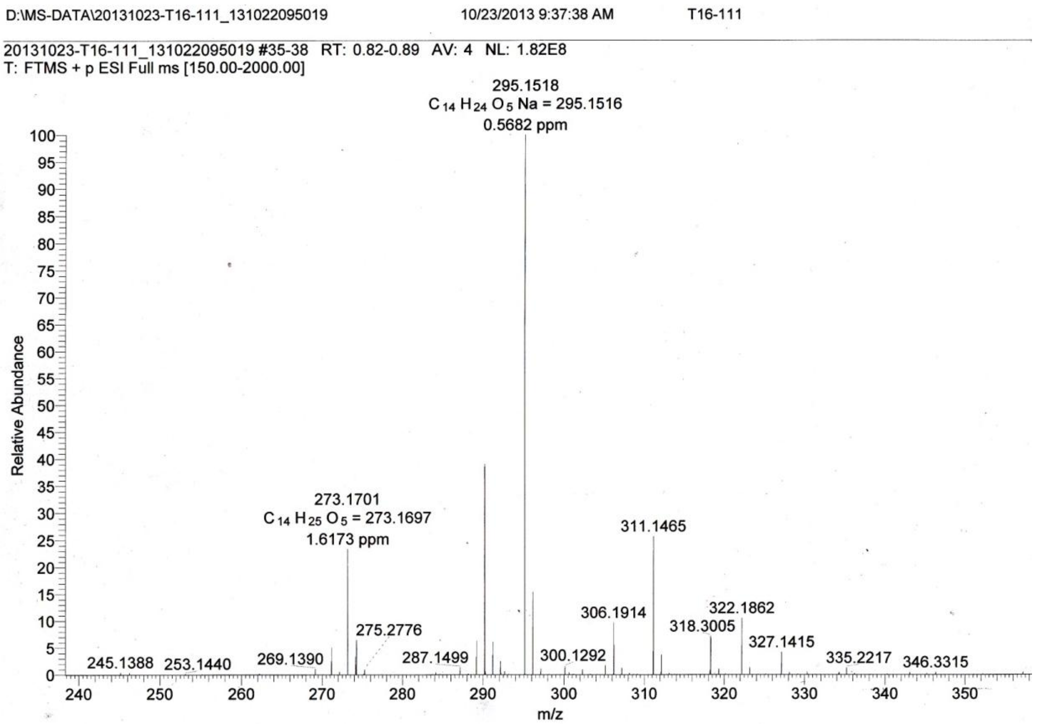

Figure S38. ^{1}H NMR spectrum of compound **8c**.

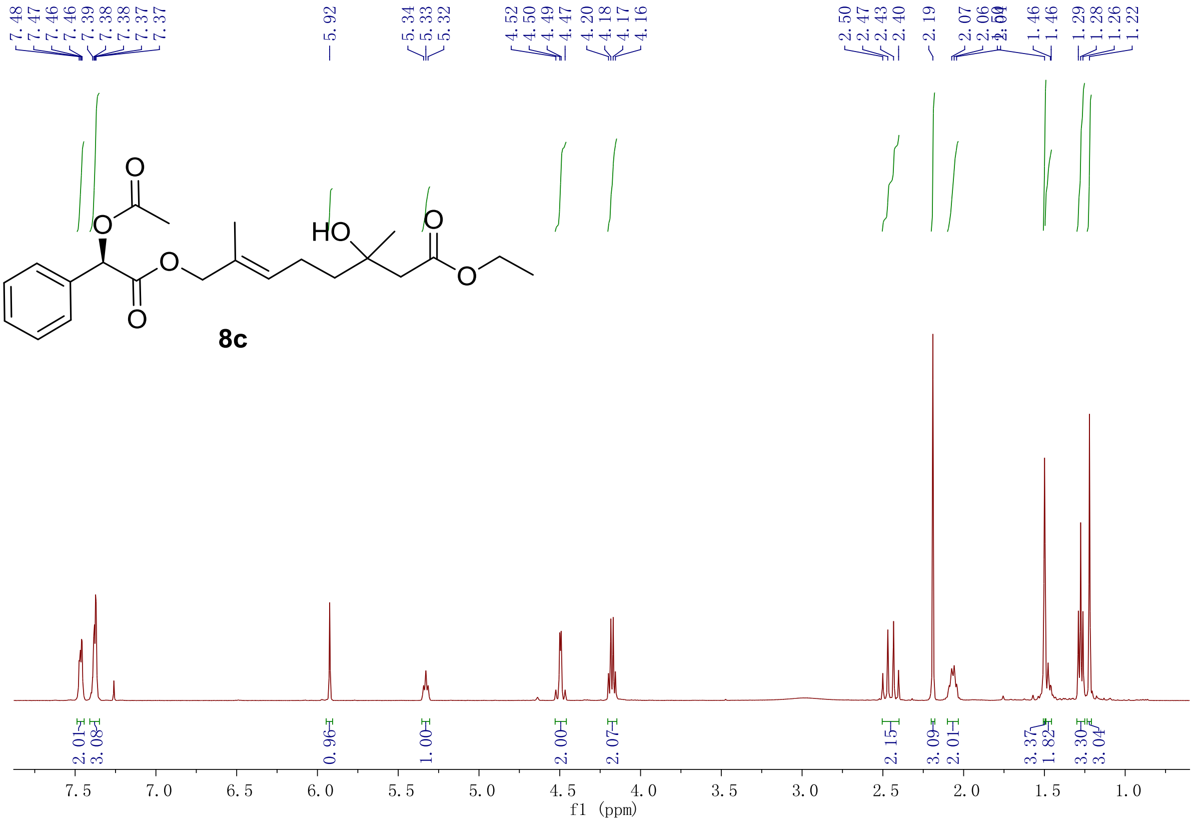

Figure S39. ^{13}C NMR spectrum of compound **8c**.

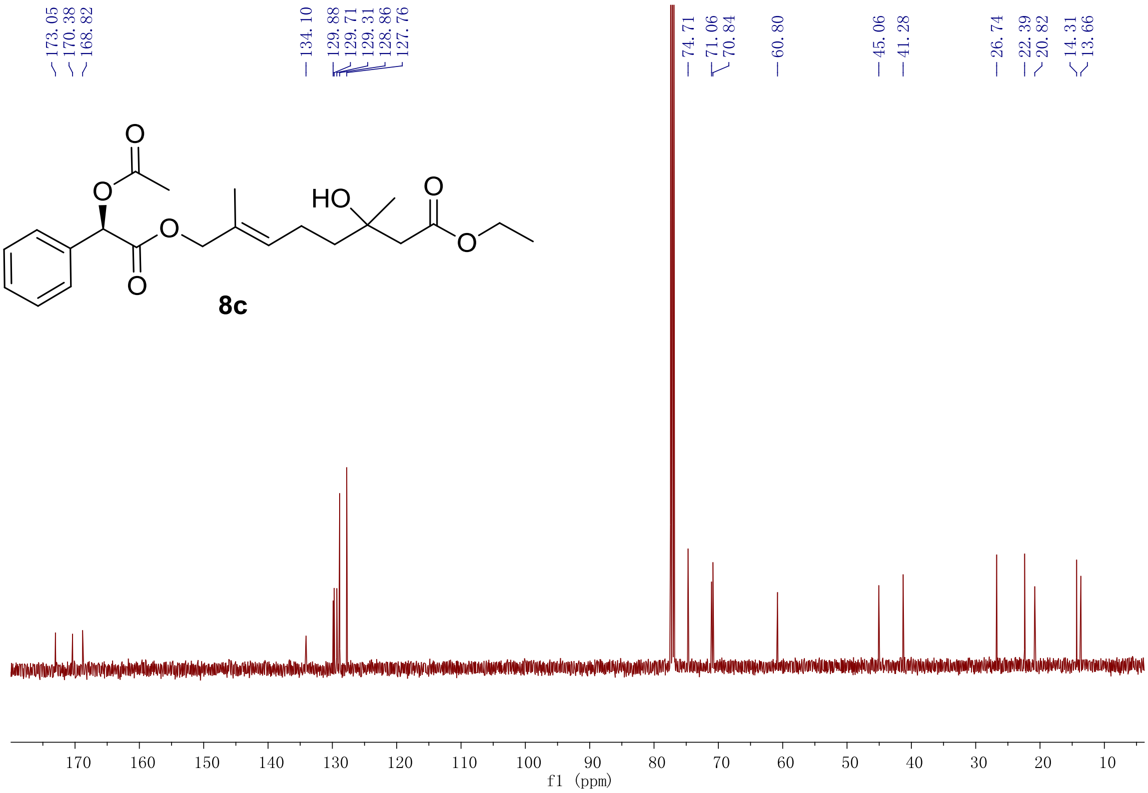

Figure S40. HRMS of compound **8c**.

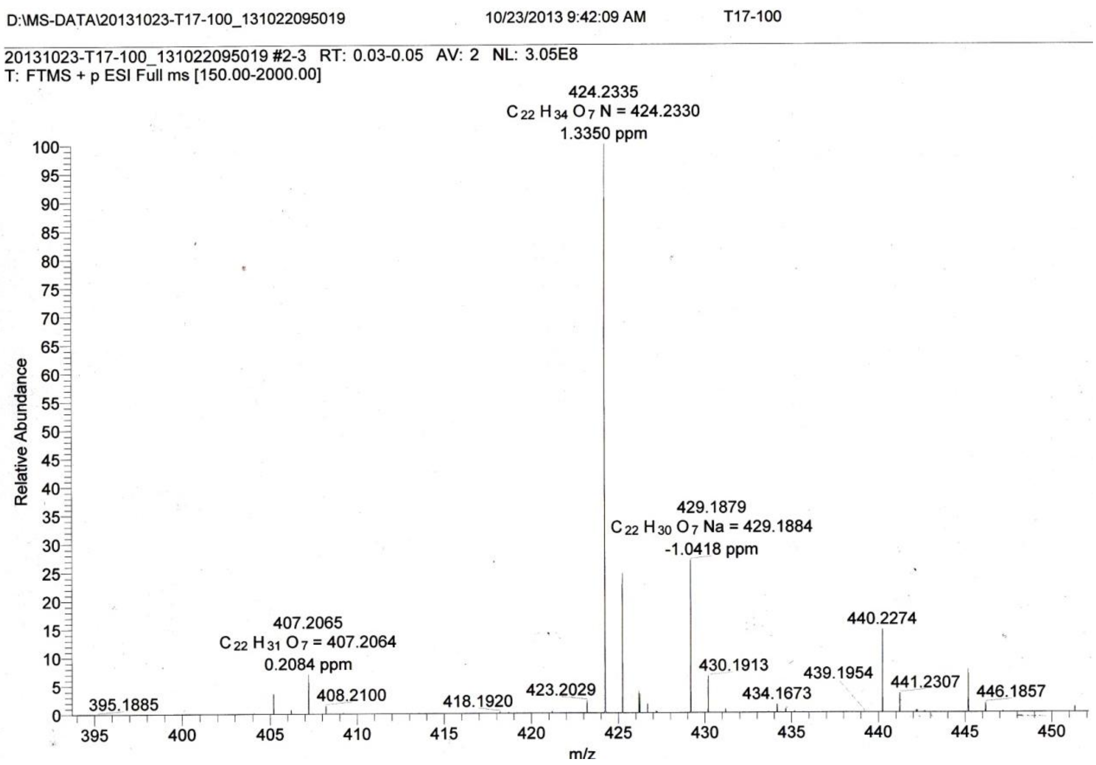

Figure S41. ^{1}H NMR spectrum of compound 9a.

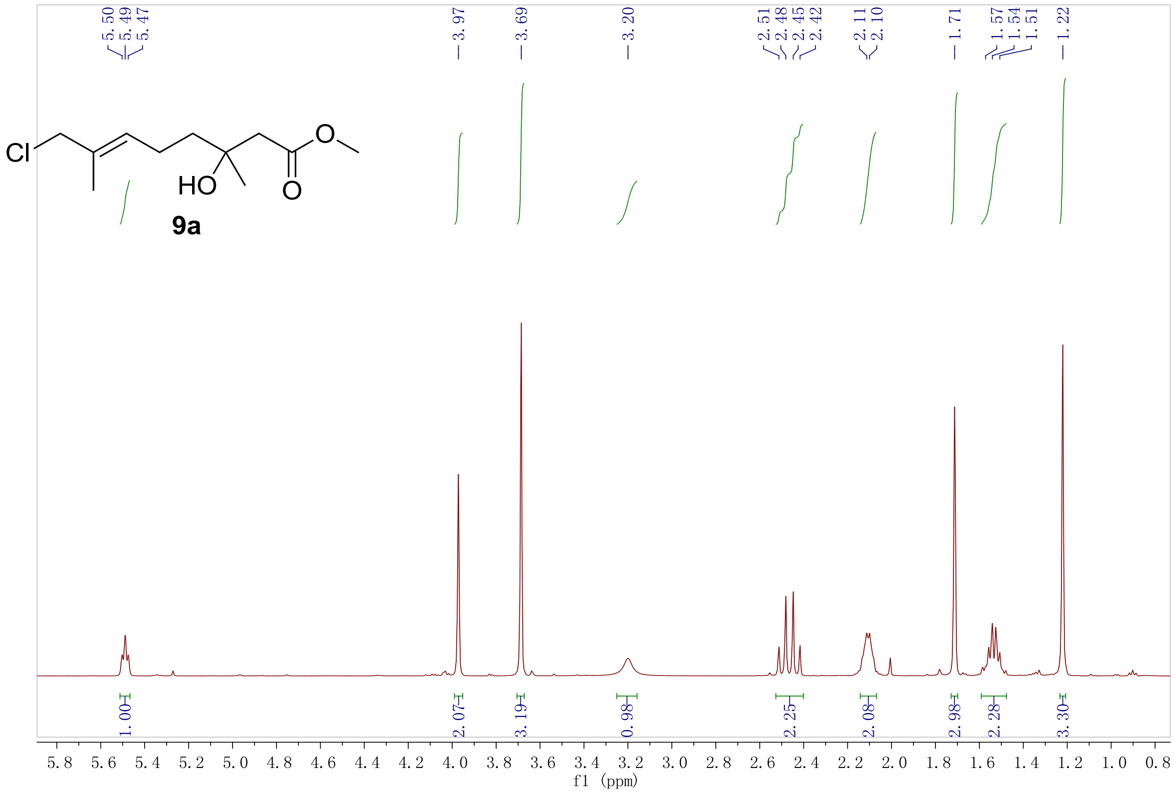

Figure S42. ^{13}C NMR spectrum of compound **9a**.

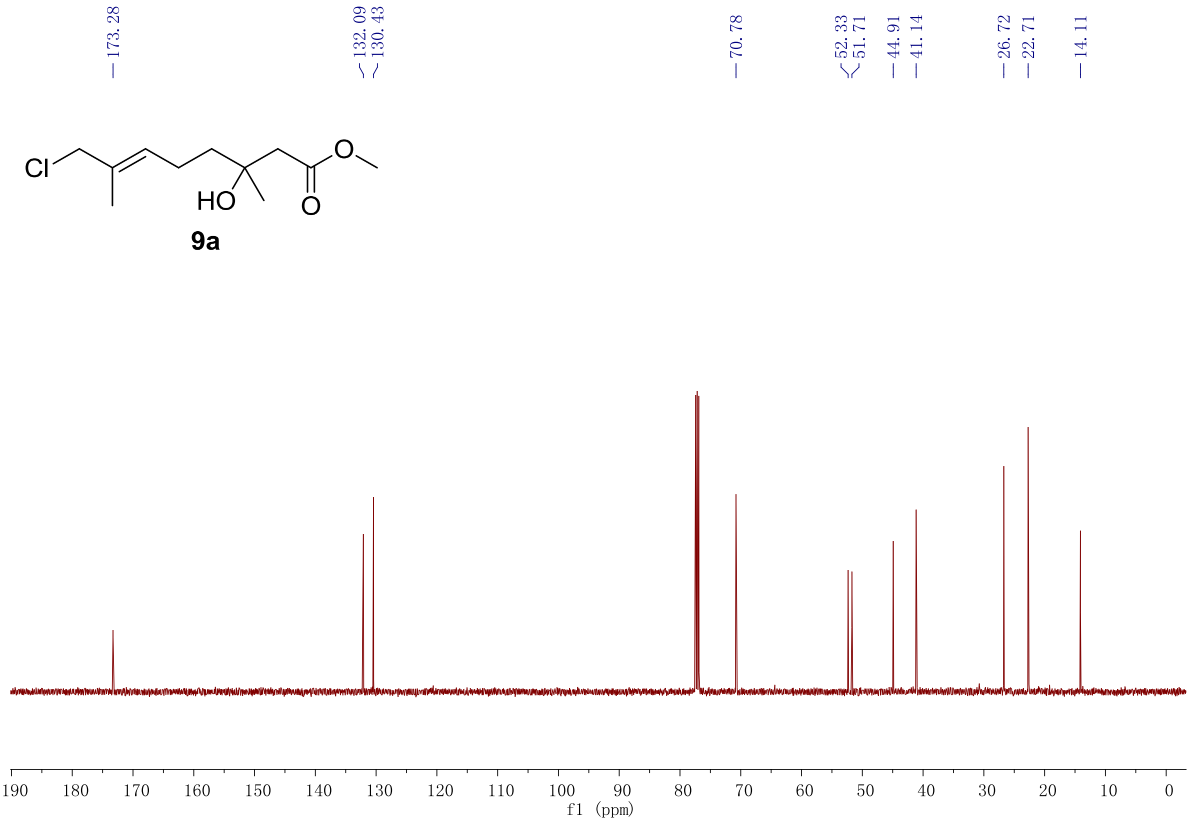

Figure S43. HRMS of compound 9a.

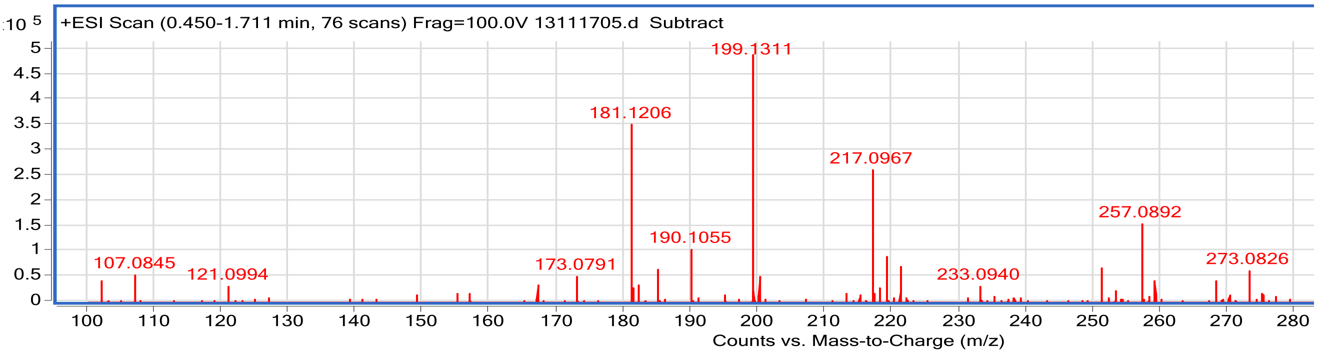

Figure S44. ^{1}H NMR spectrum of compound **9b**.

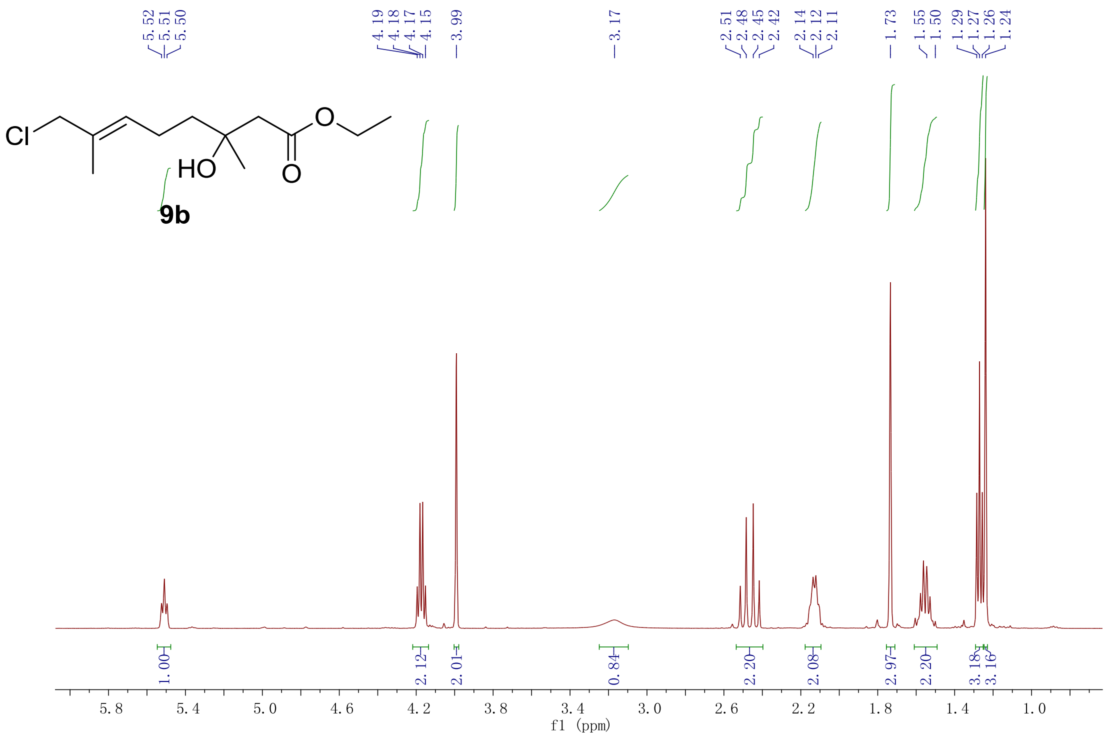

Figure S45. ^{13}C NMR spectrum of compound **9b**.

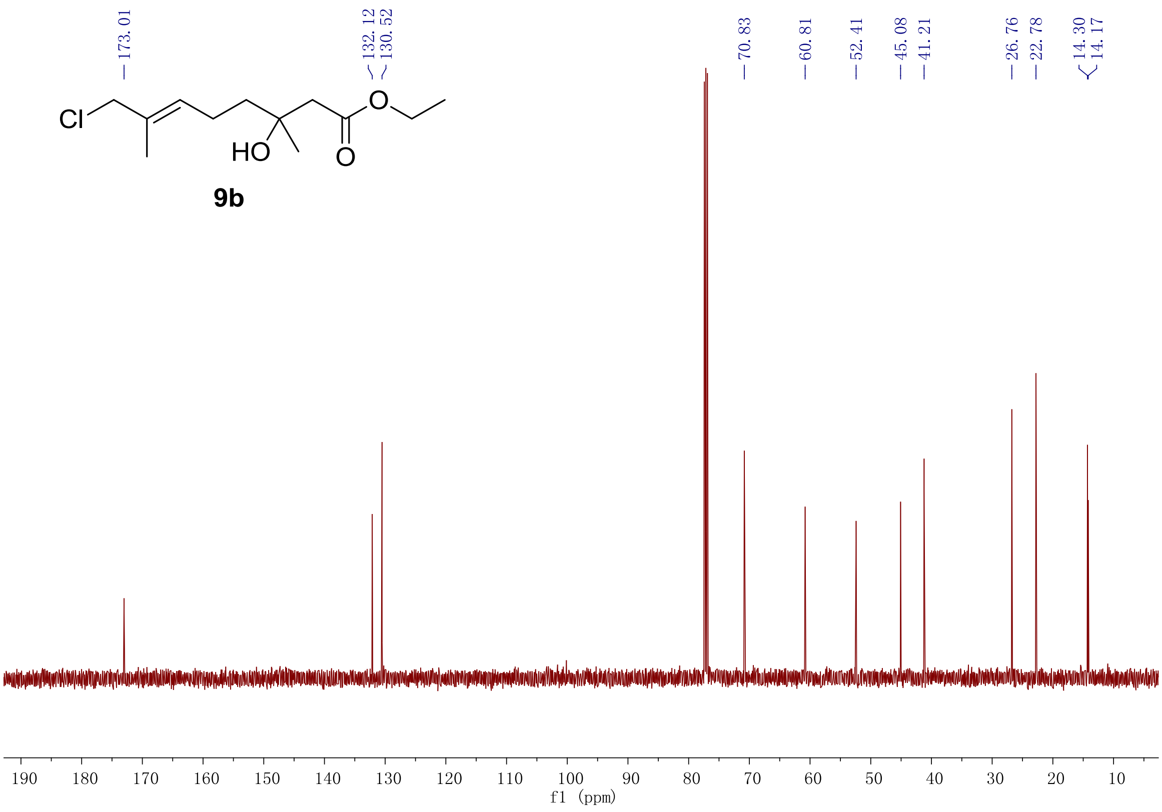

Figure S46. HRMS of compound **9b**.

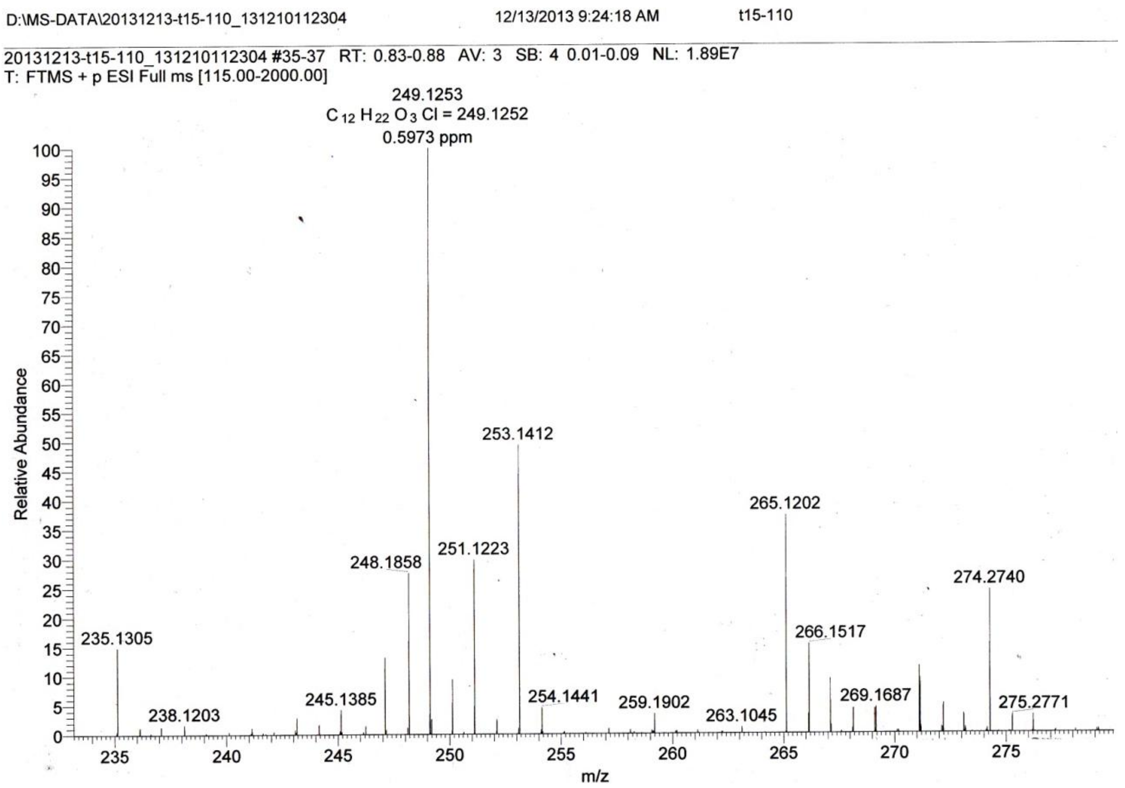

Figure S47. ^{1}H NMR spectrum of compound **10**.

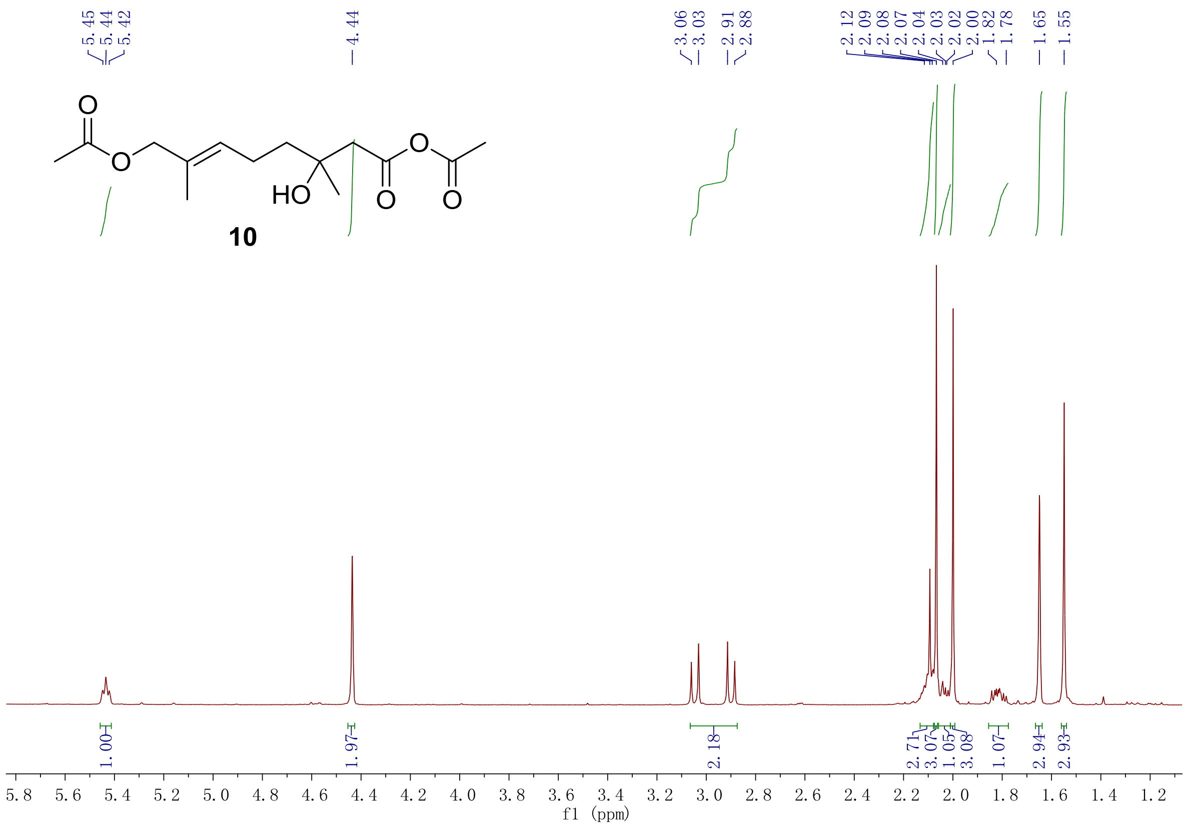

Figure S48. ^{13}C NMR spectrum of compound **10**.

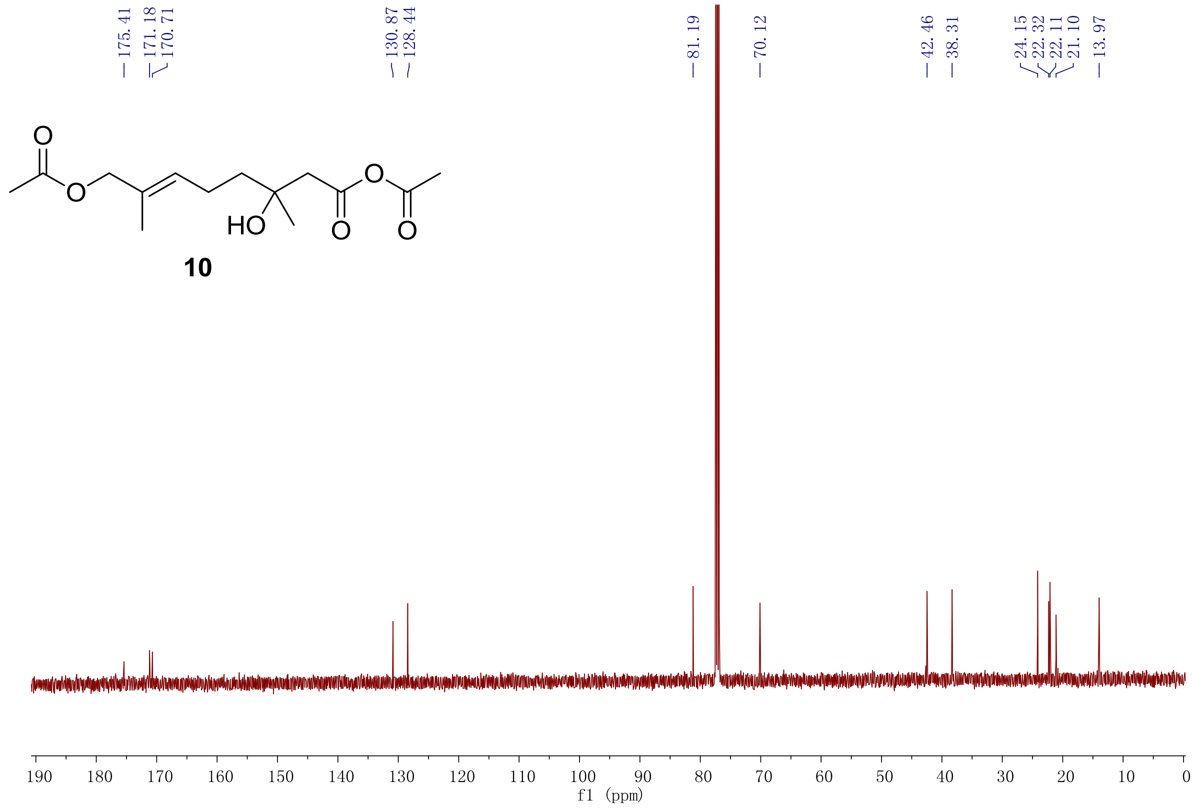

Figure S49. HRMS of compound 10.

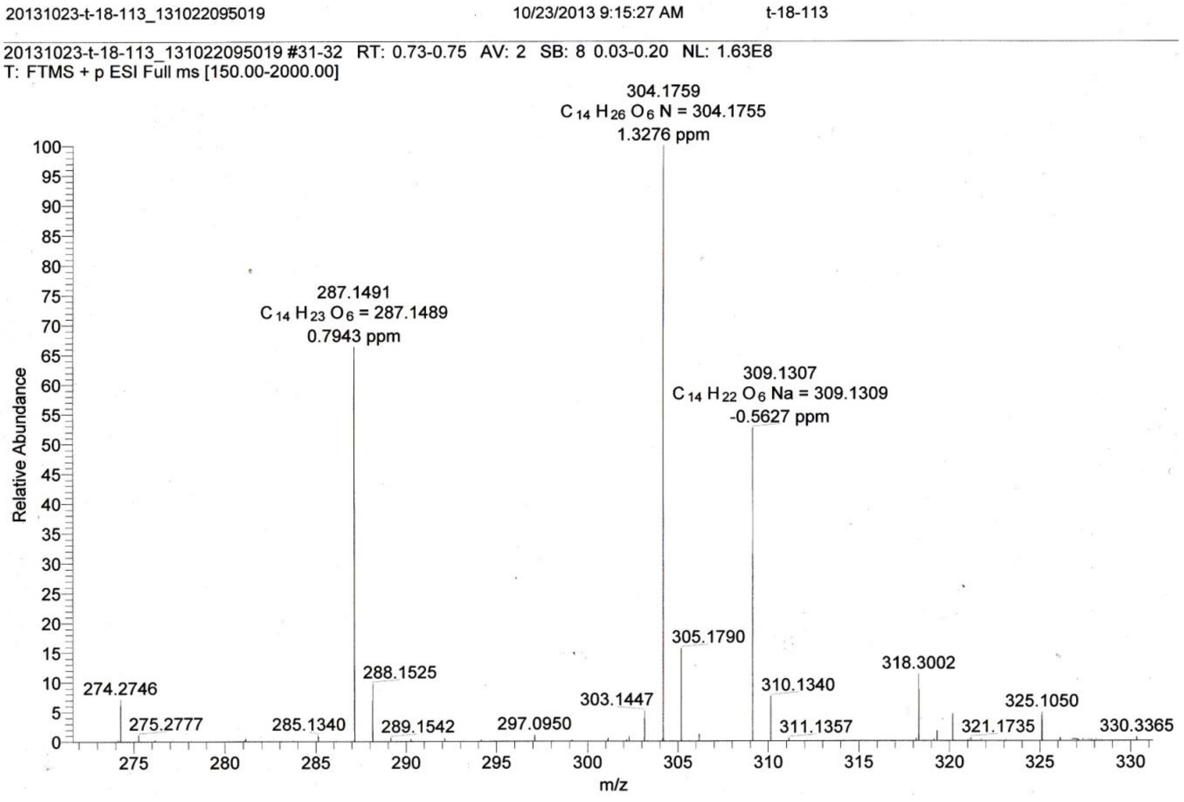

Figure S50. ^{1}H NMR spectrum of compound **11**.

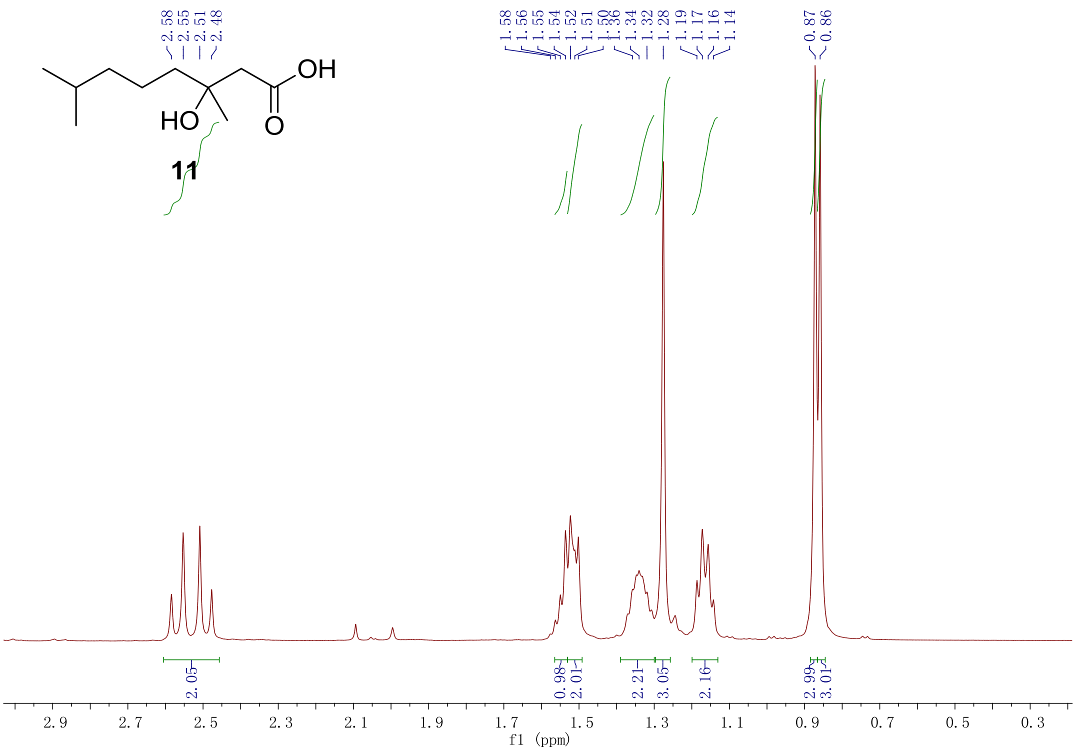

Figure S51. ^{13}C NMR spectrum of compound **11**.

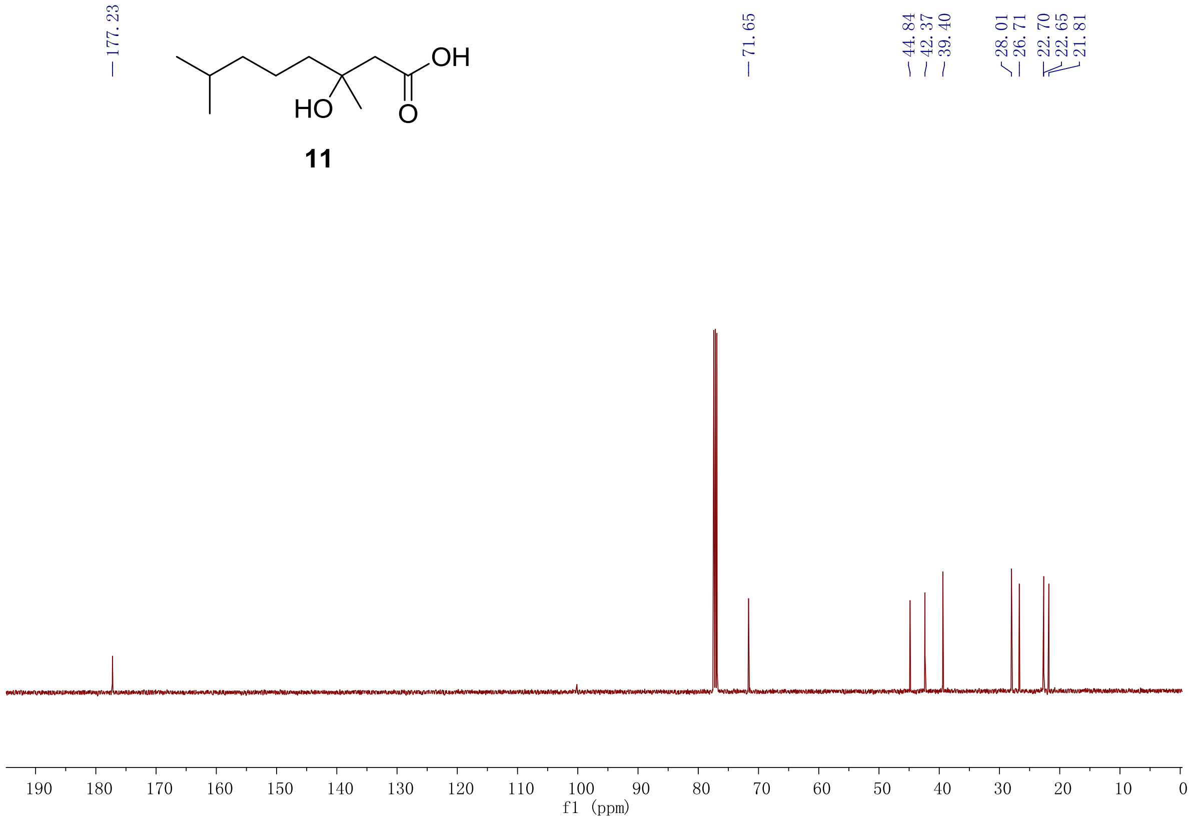

Figure S52. HRMS of compound **11**.

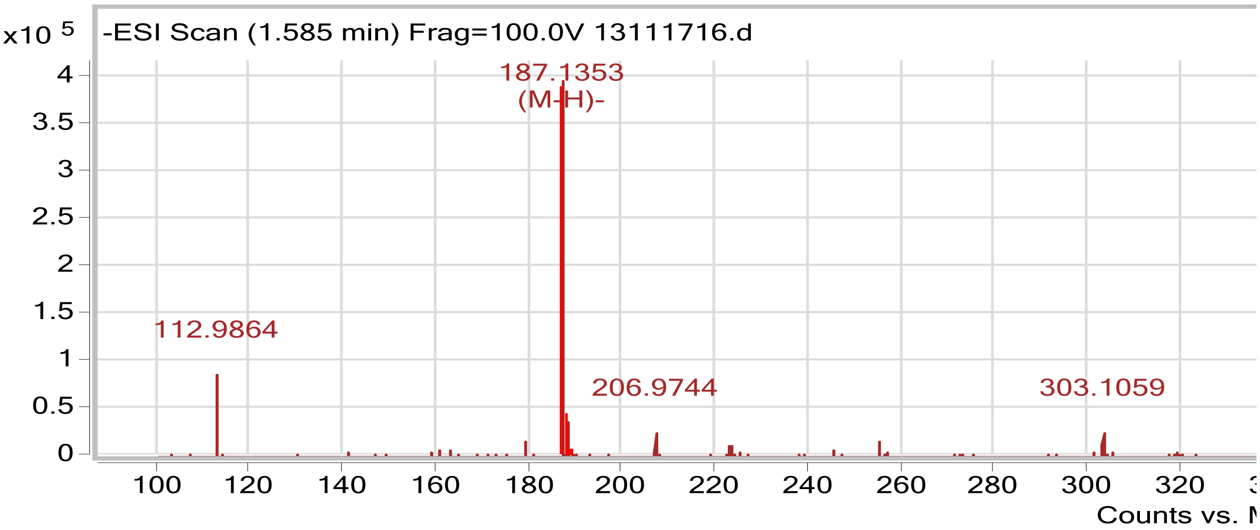

Figure S53. ^{1}H NMR spectrum of compound **12**.

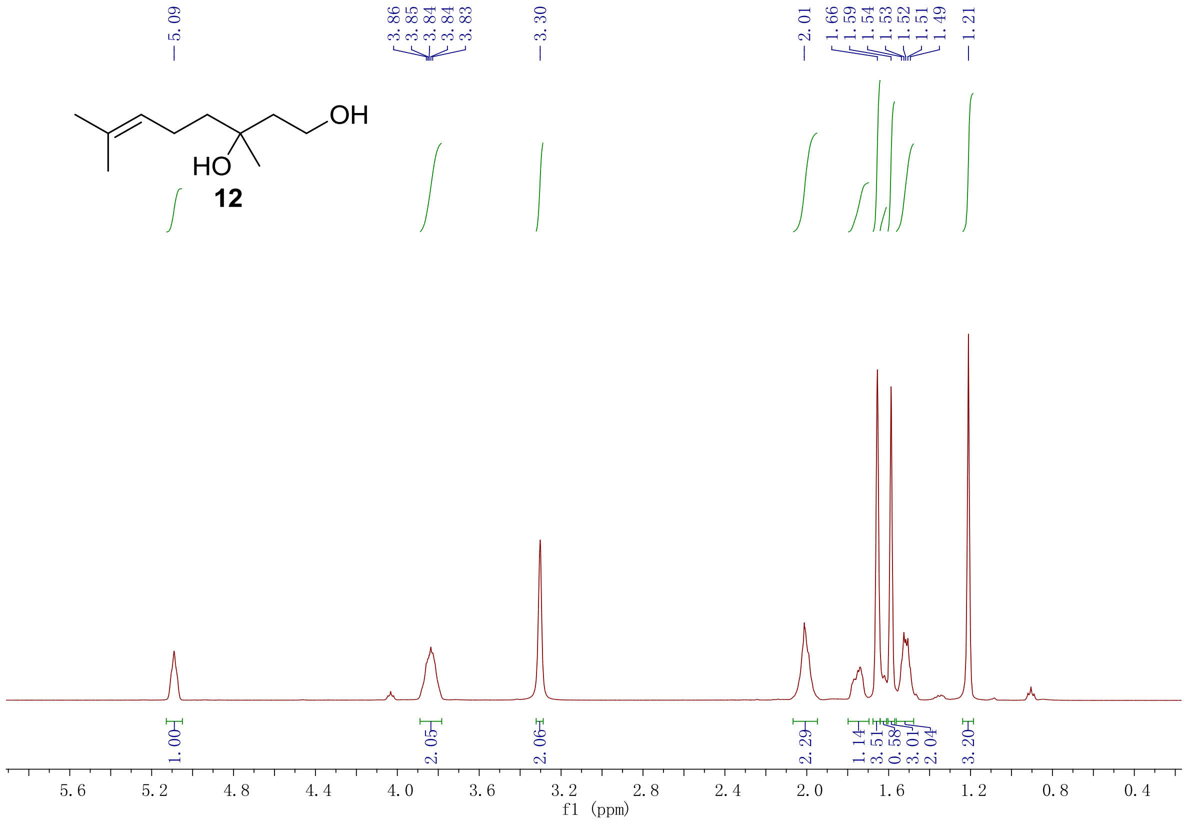

Figure S54. ^{13}C NMR spectrum of compound **12**.

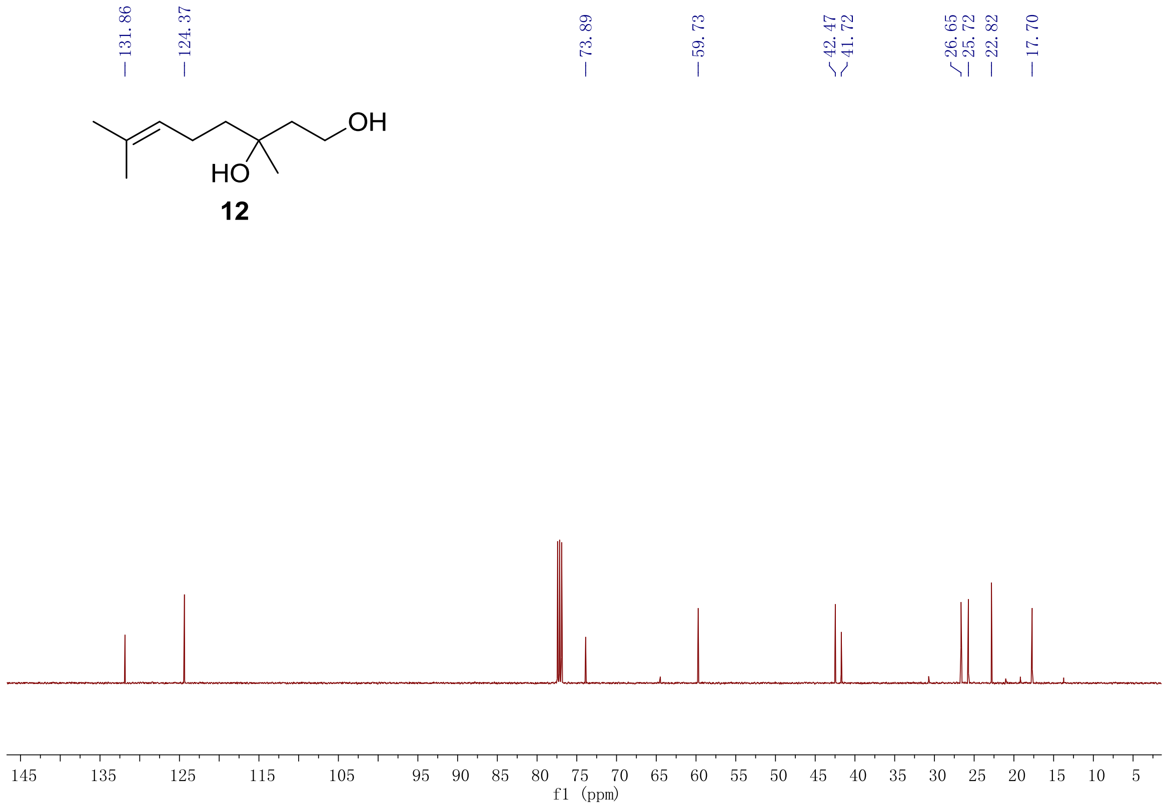

Figure S55. HRMS of compound **12**.

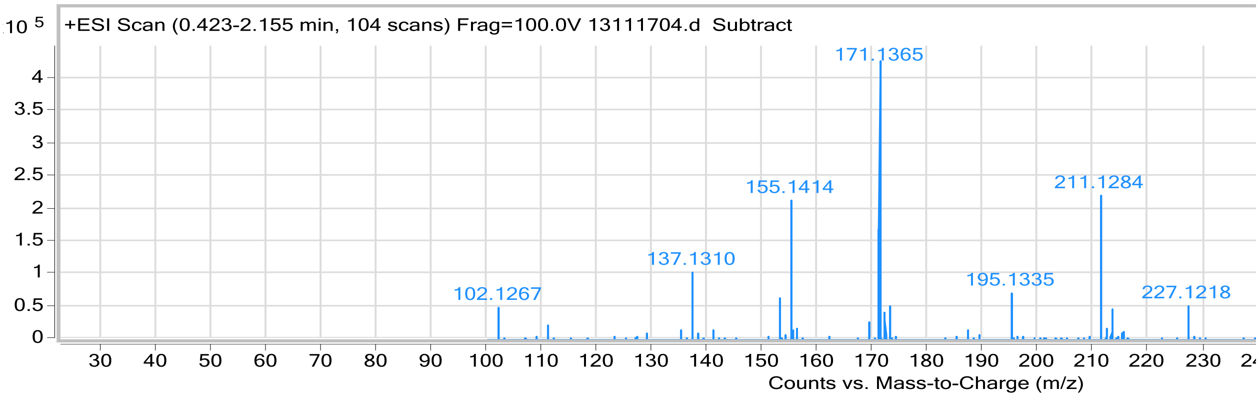

Figure S56. ^{1}H NMR spectrum of compound **13**.

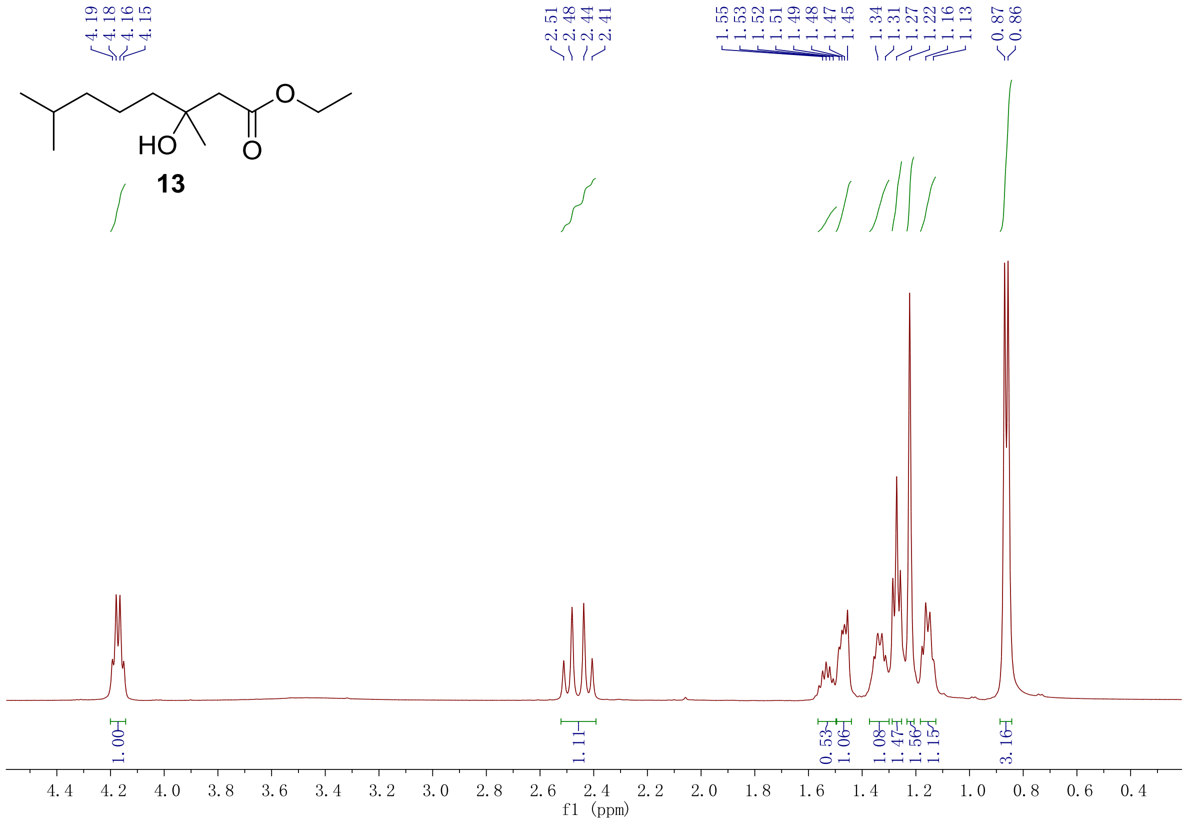

Figure S57. ^{13}C NMR spectrum of compound **13**.

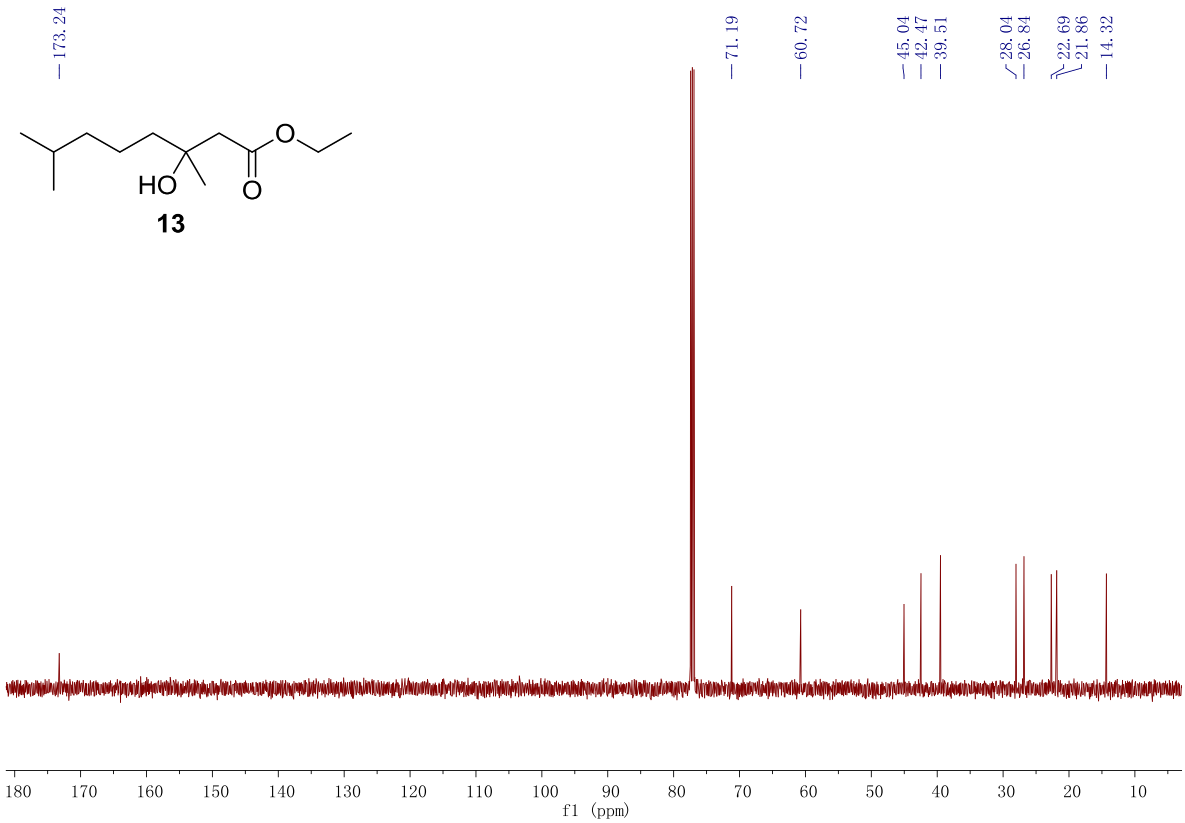

Figure S58. HRMS of compound **13**.

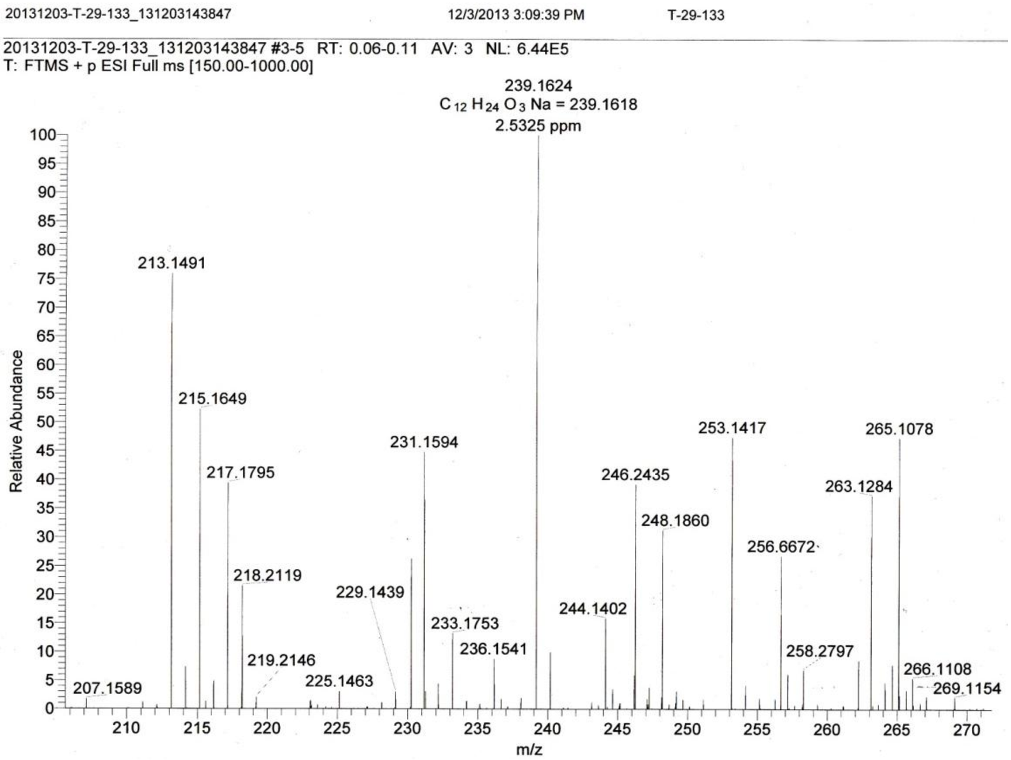

Figure S59. ^{1}H NMR spectrum of compound **14**.

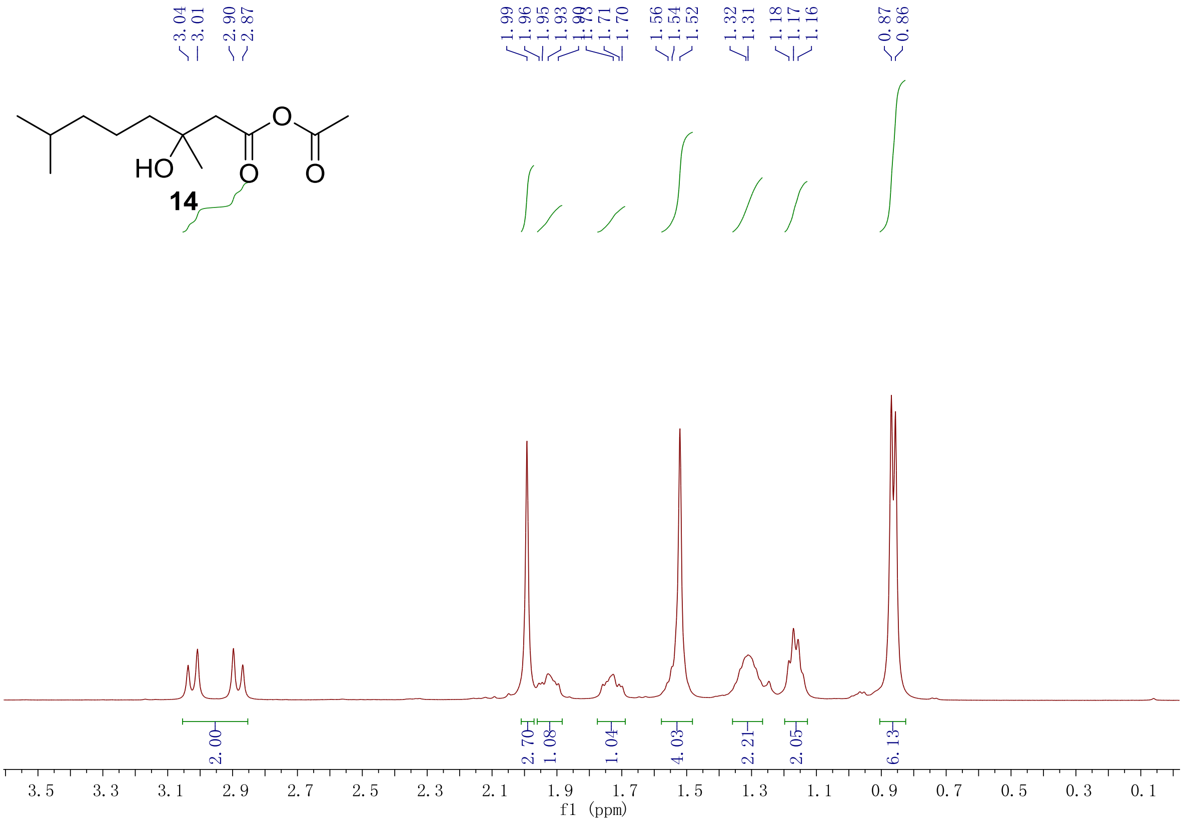

Figure S60. ^{13}C NMR spectrum of compound **14**.

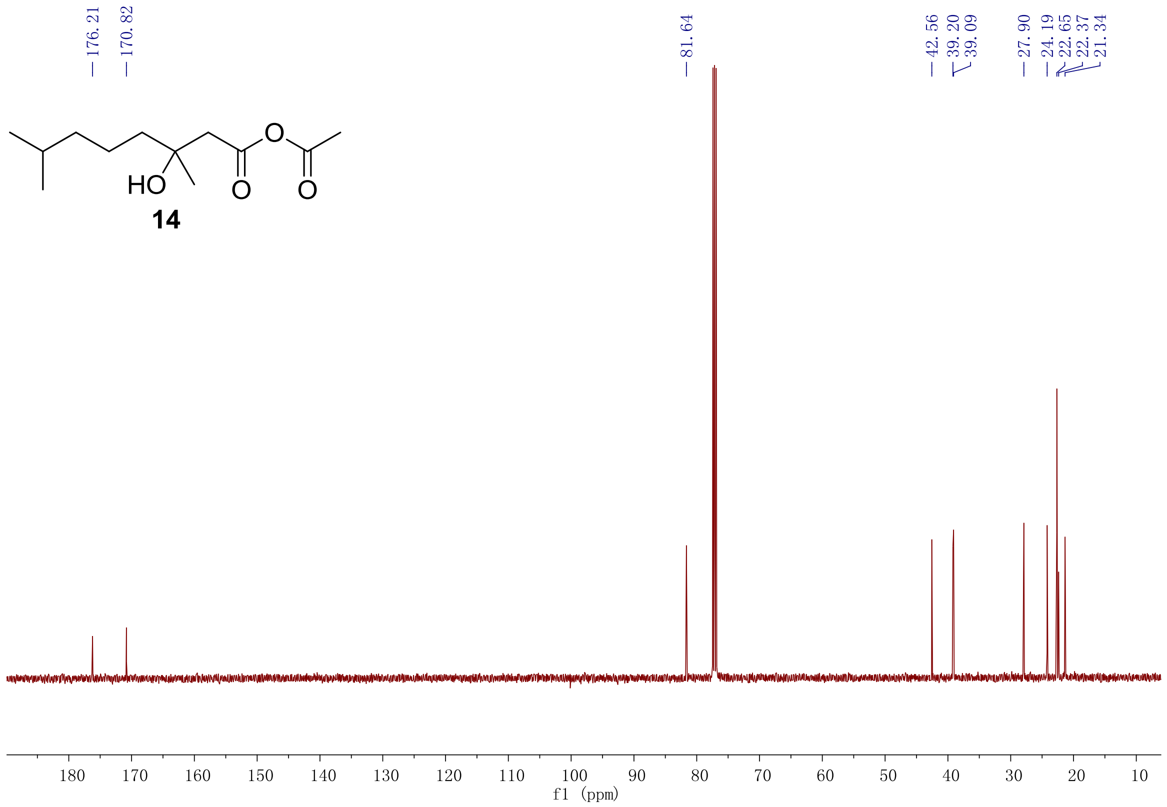

Figure S61. HRMS of compound 14.

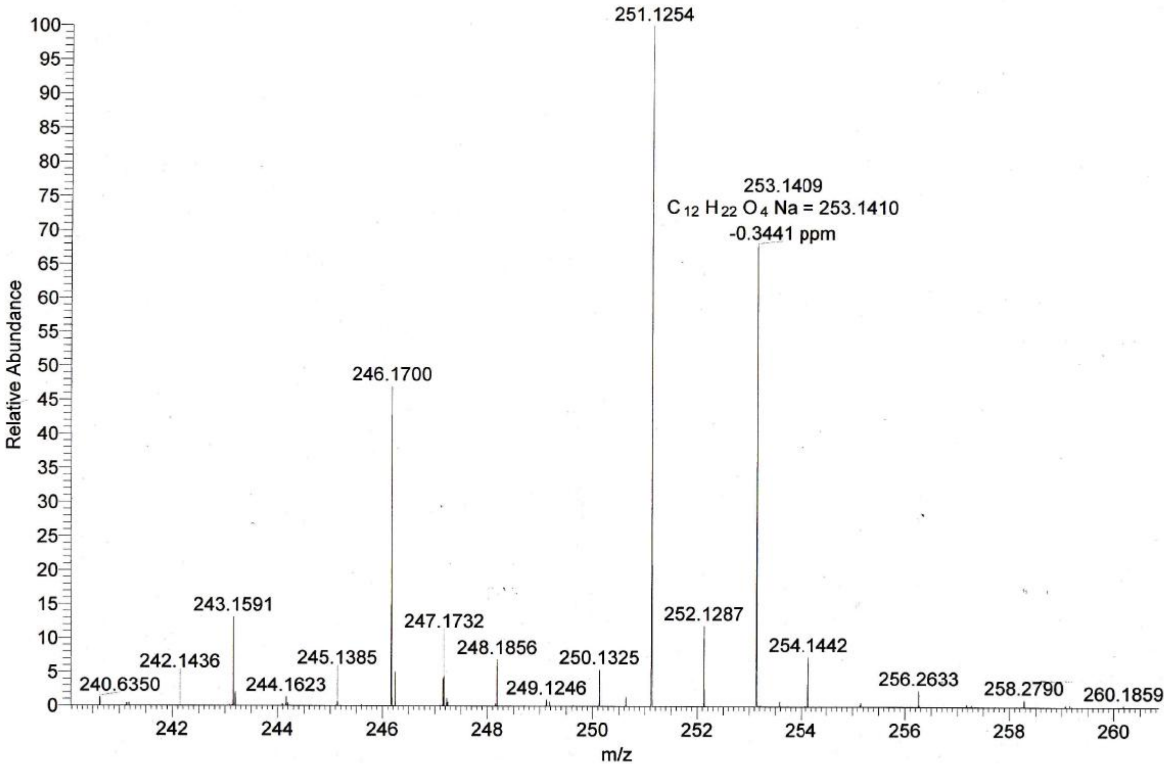

Figure S62. ^{1}H NMR spectrum of compound **15**.

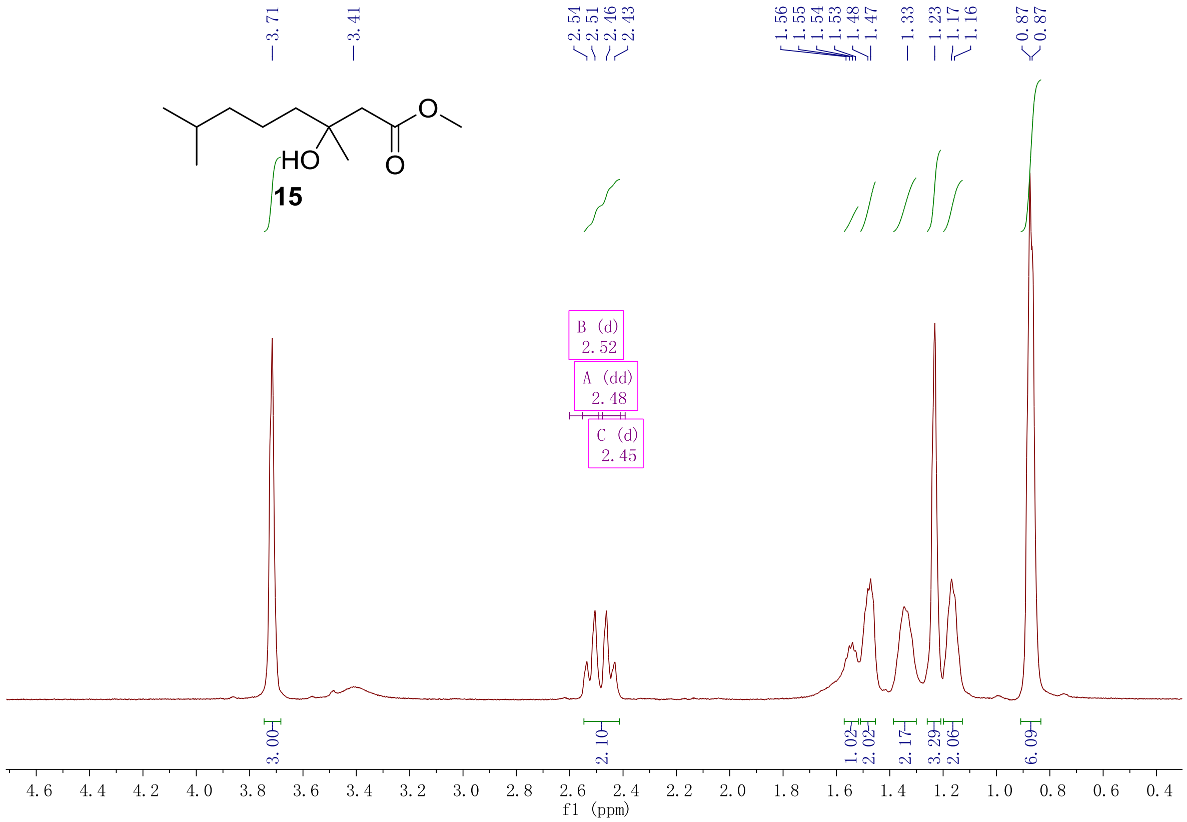

Figure S63. ^{13}C NMR spectrum of compound **15**.

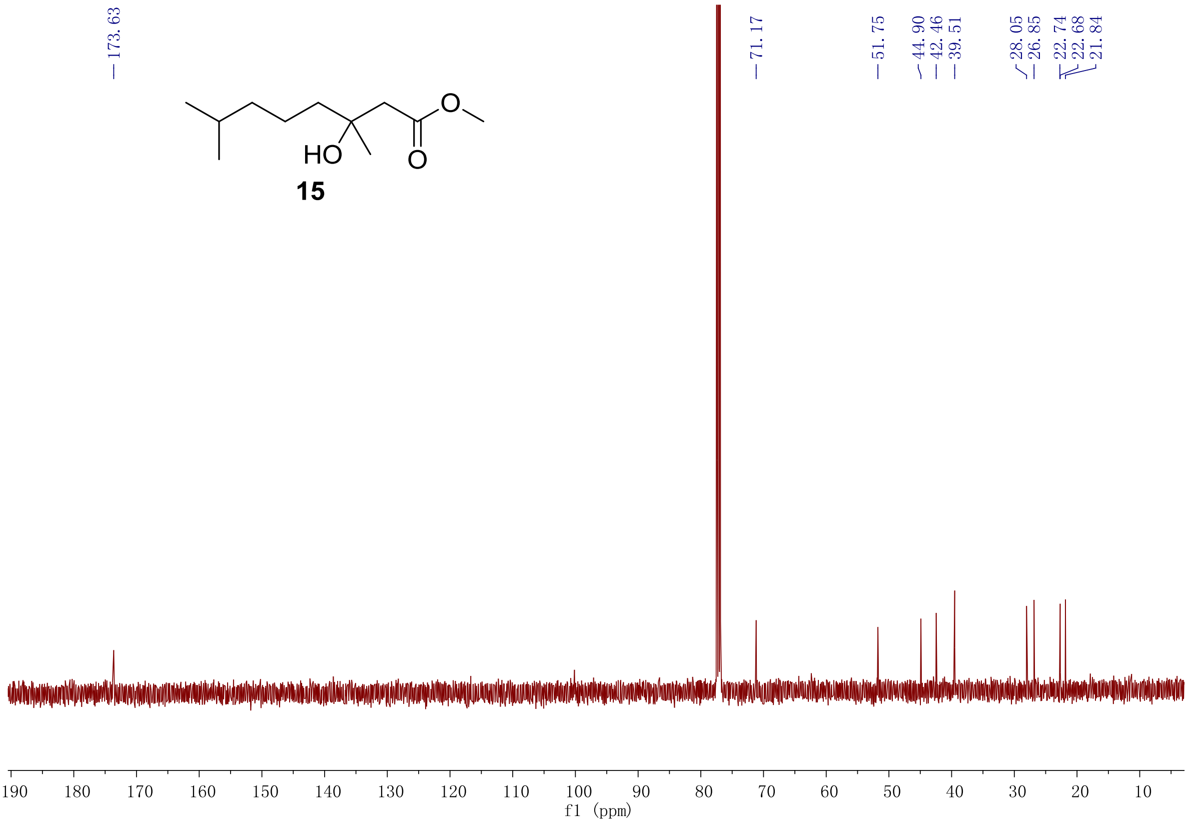

Figure S64. HRMS of compound **15**.

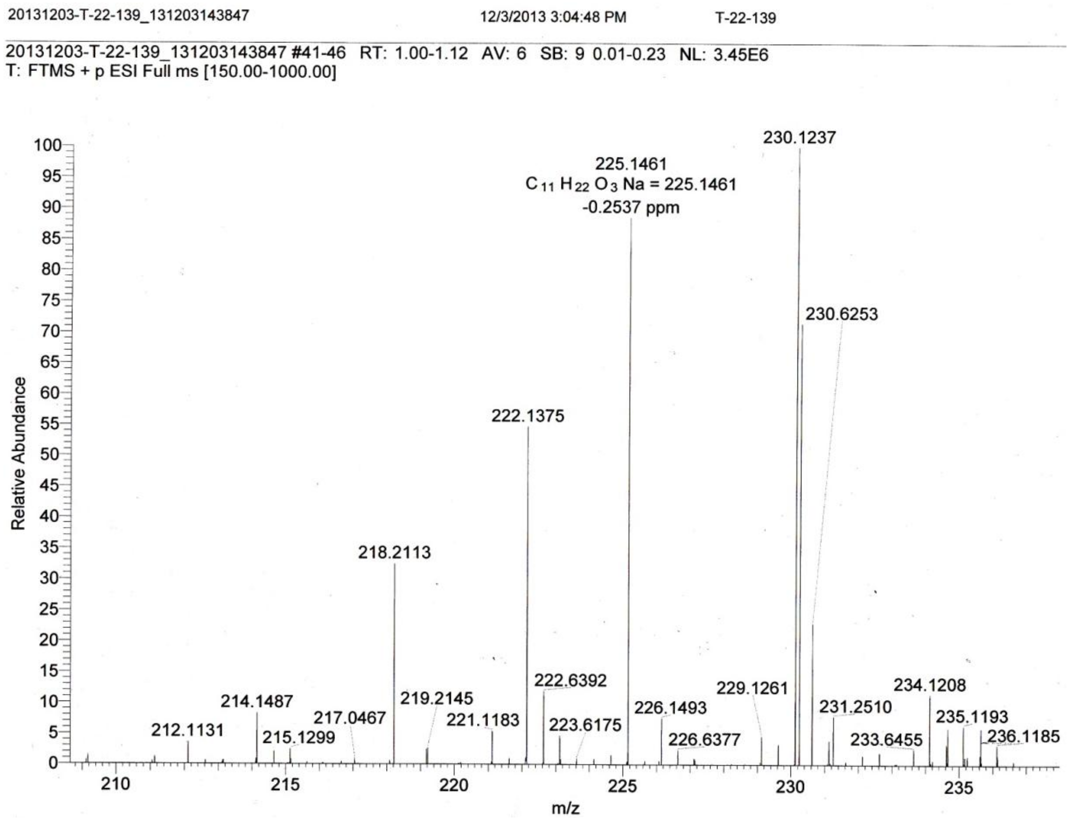

Figure S65. ^{1}H NMR spectrum of compound **16**.

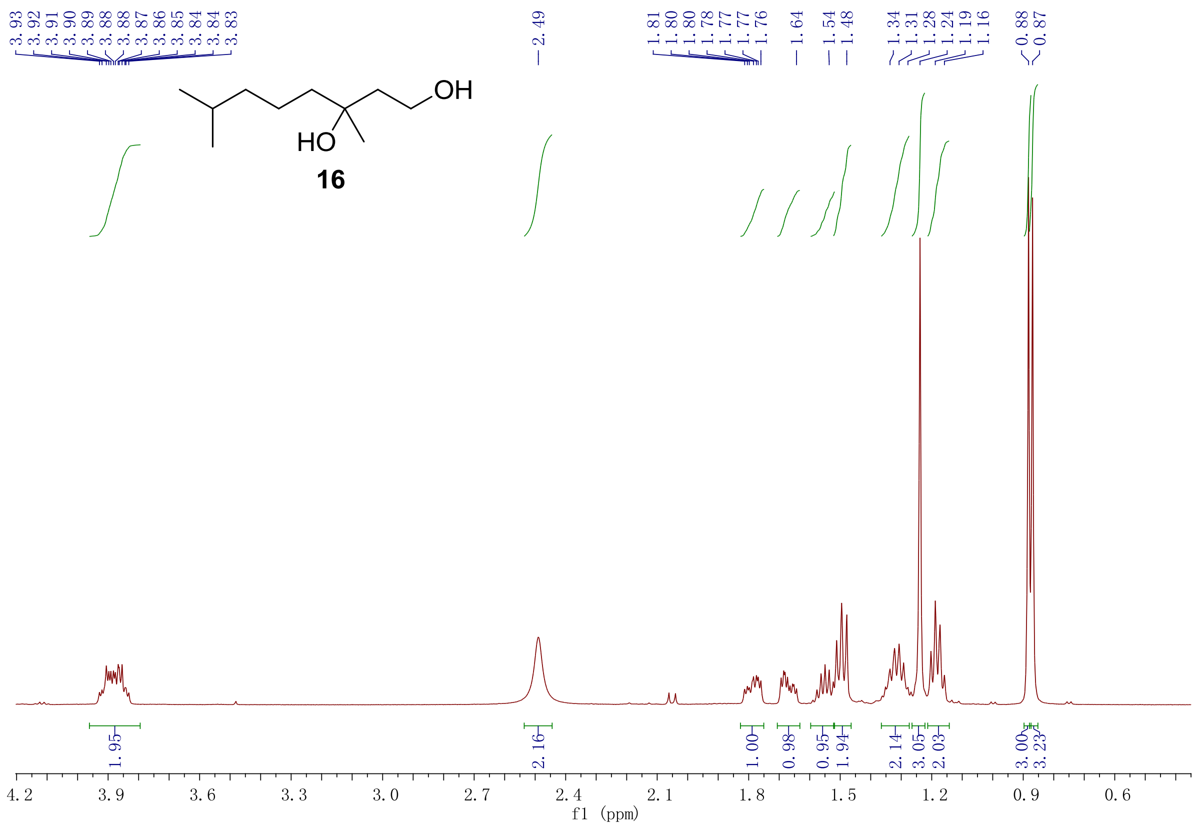

Figure S66. ^{13}C NMR spectrum of compound **16**.

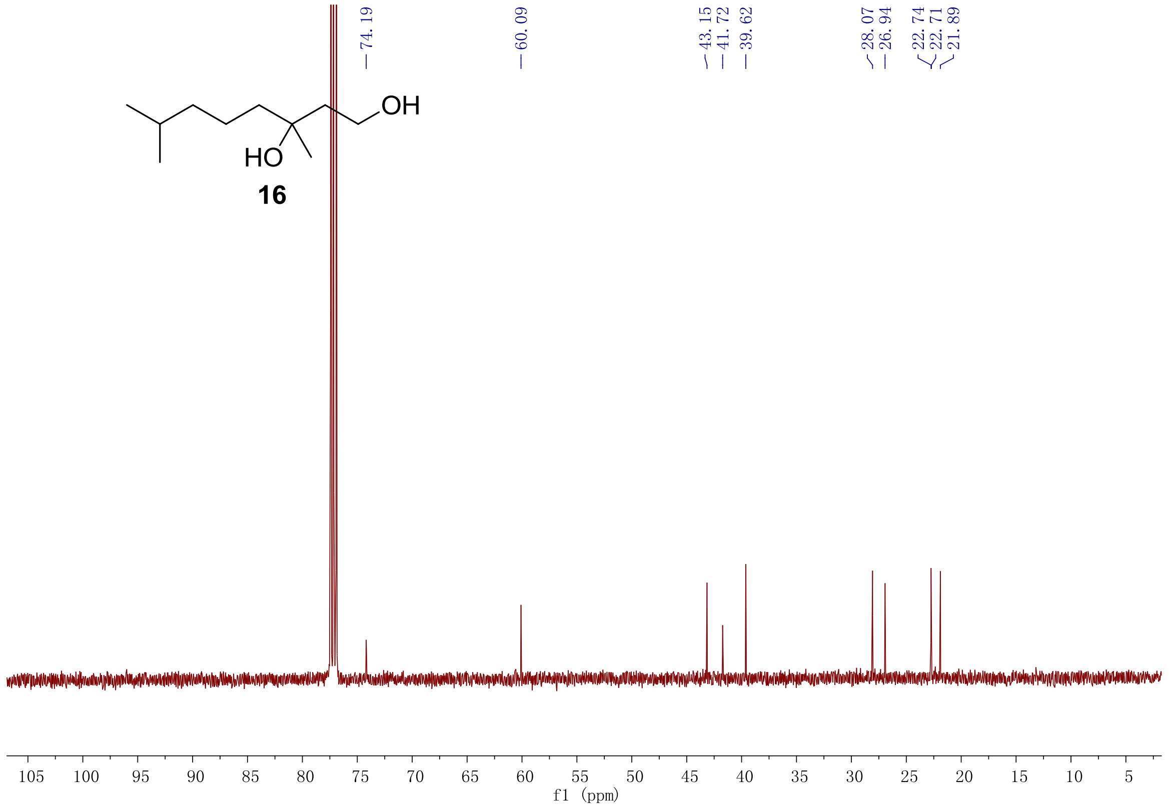

Figure S67. HRMS of compound **16**.

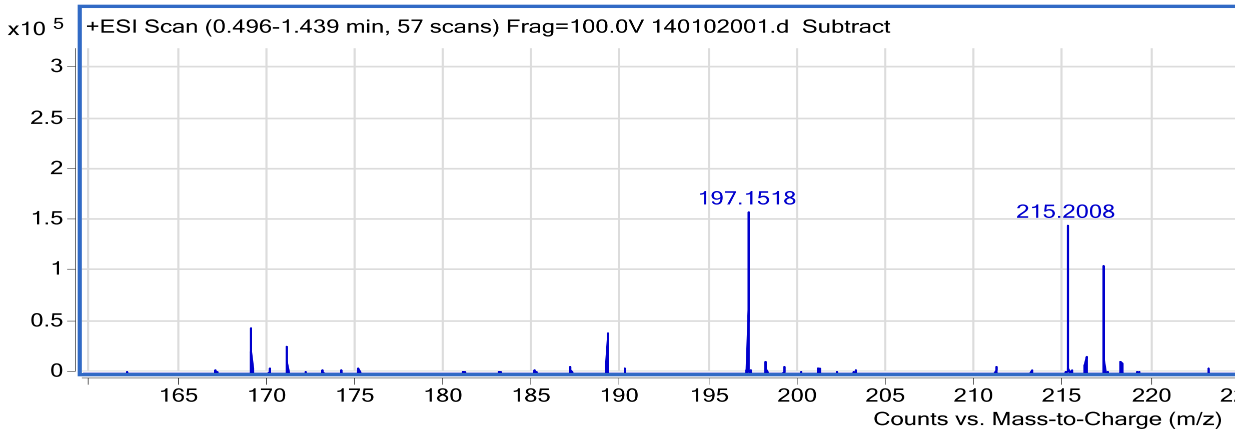

Figure S68. ^{1}H NMR spectrum of compound **17**.

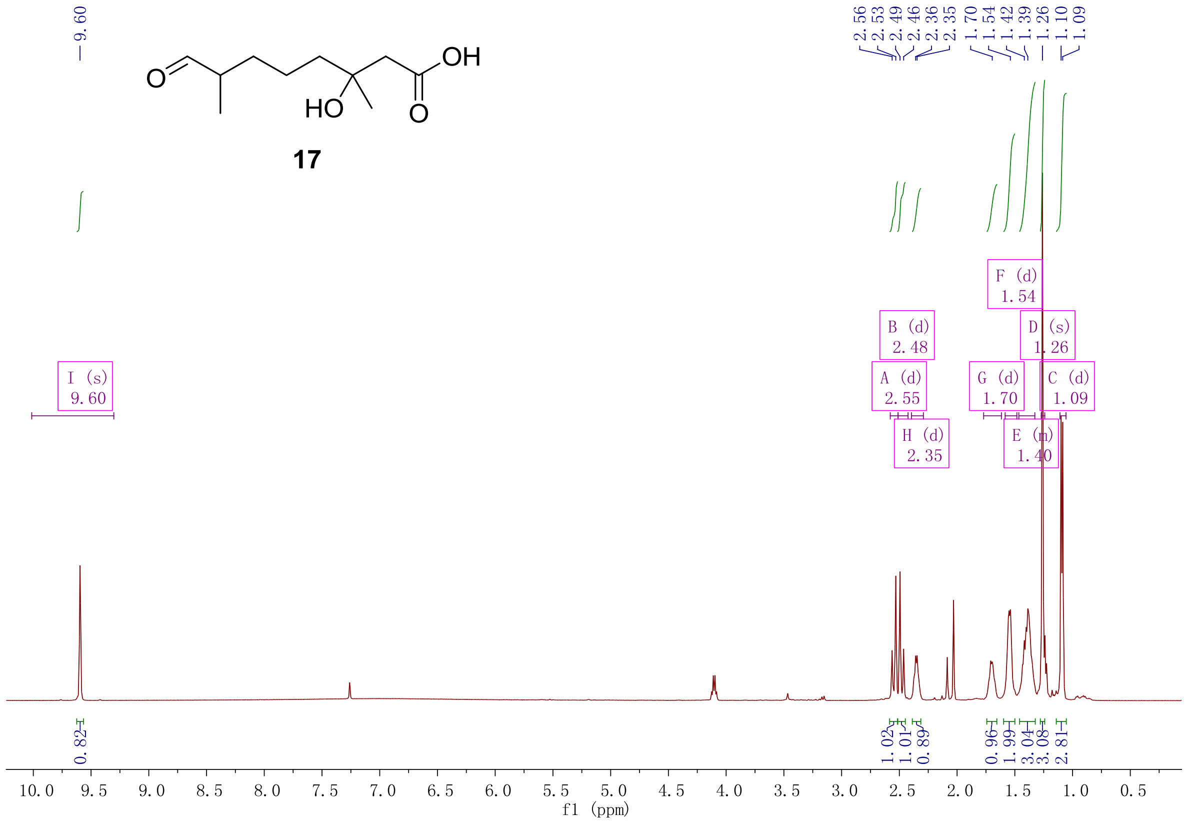

Figure S69. ^{13}C NMR spectrum of compound **17**.

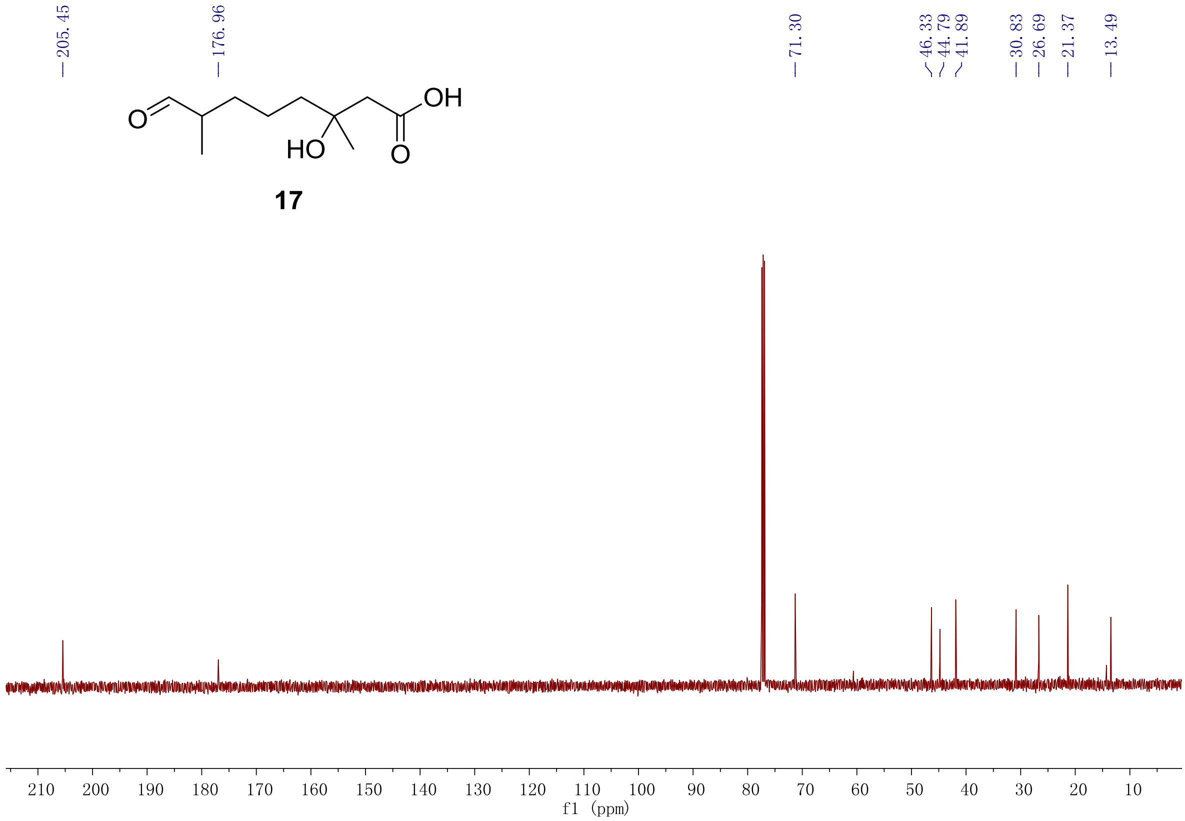

Figure S70. HRMS of compound **17**.

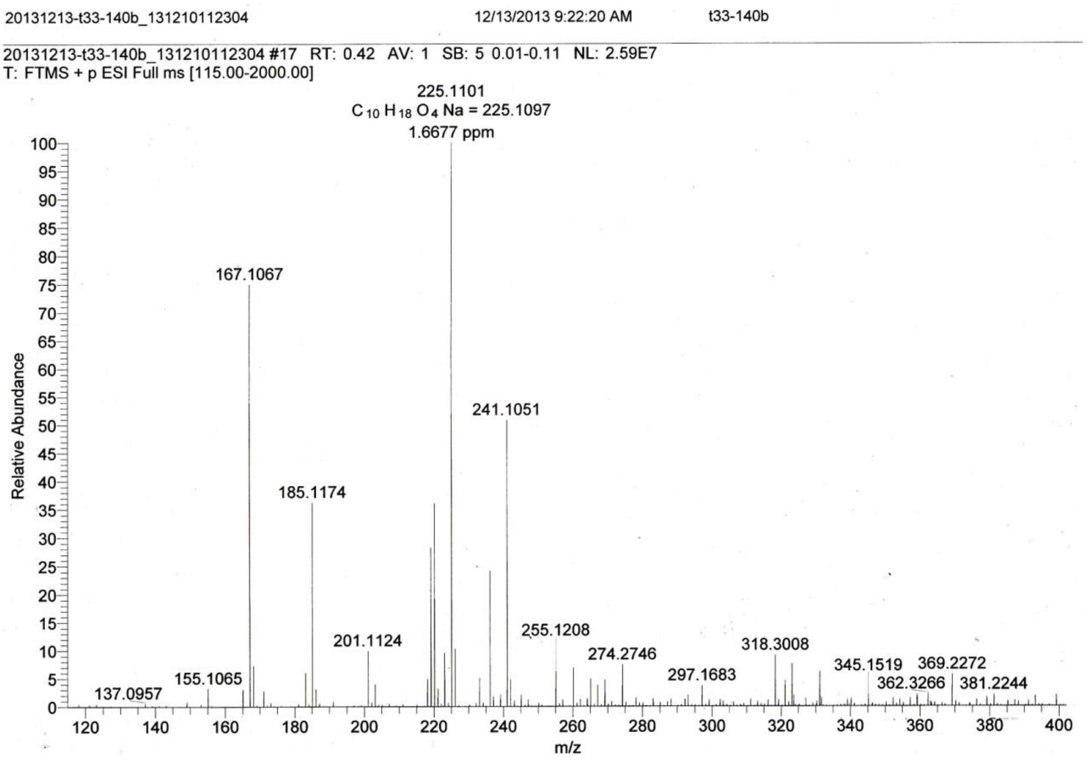

Figure S71. ^{1}H NMR spectrum of compound 18.

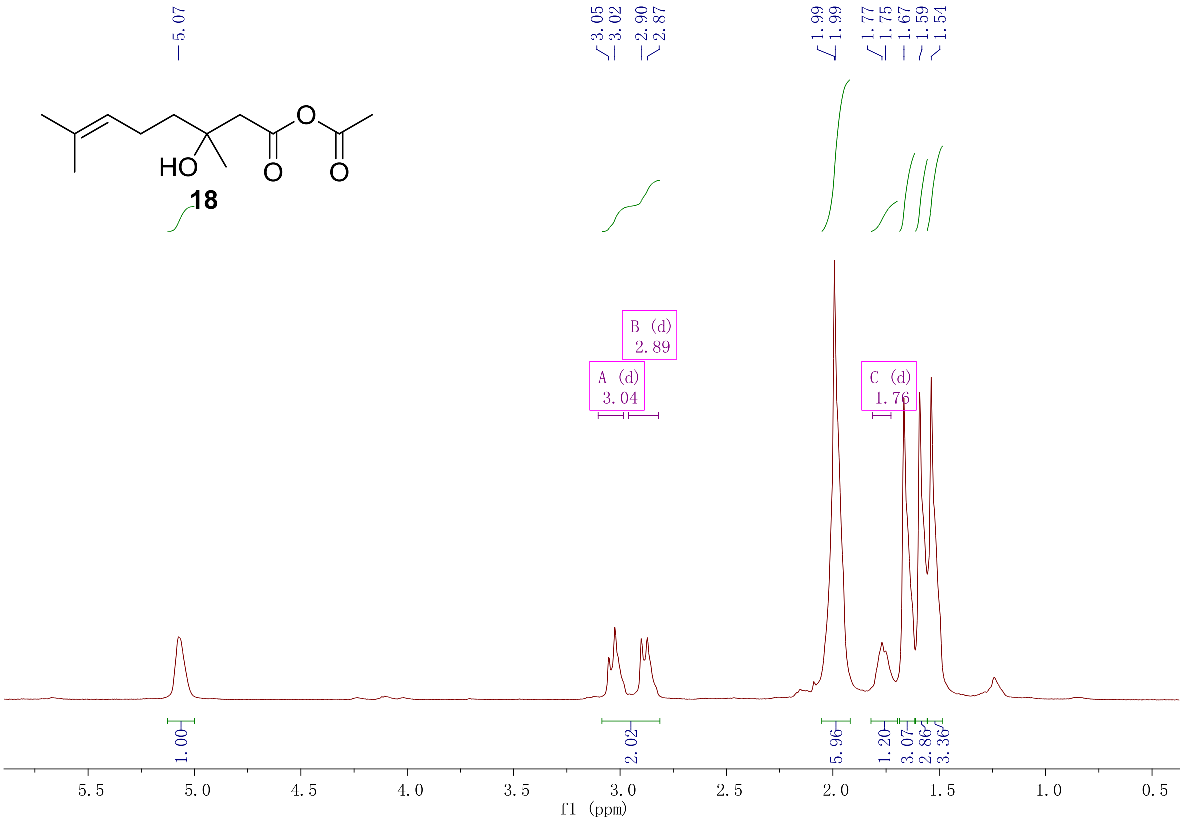

Figure S72. ^{13}C NMR spectrum of compound **18**.

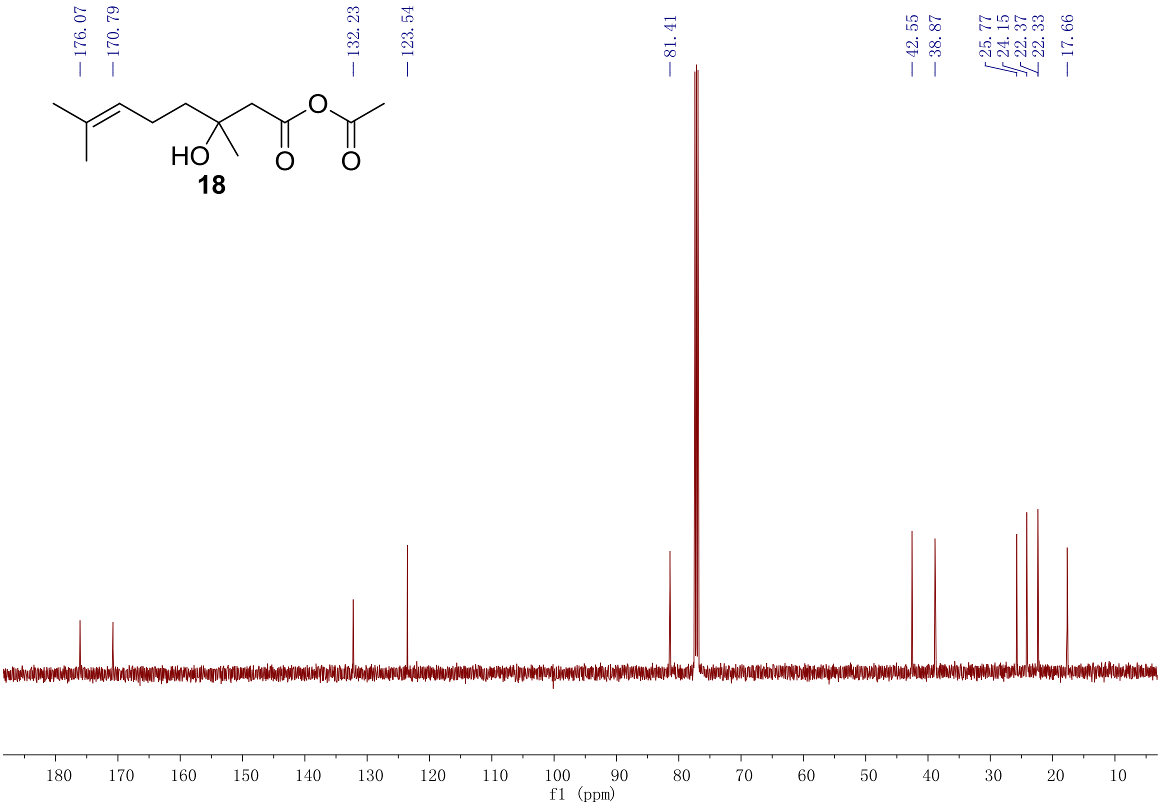

Figure S73. HRMS of compound 18.

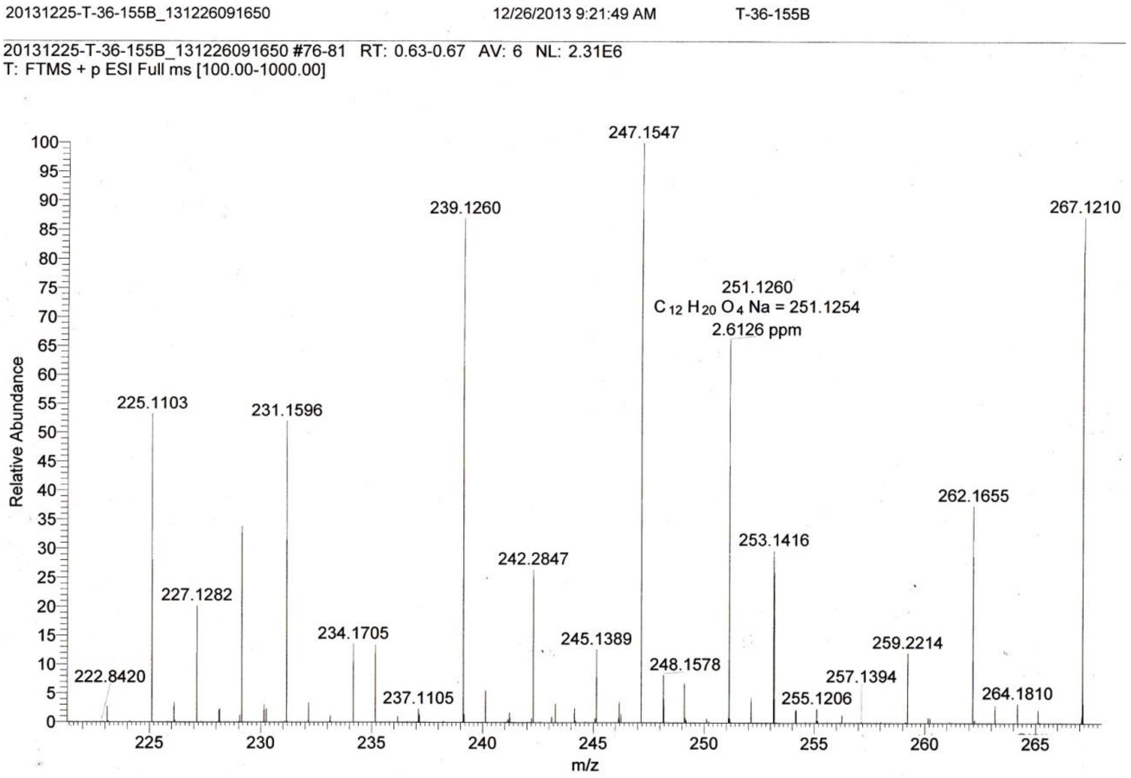

Figure S74. ^{1}H NMR spectrum of compound **19**.

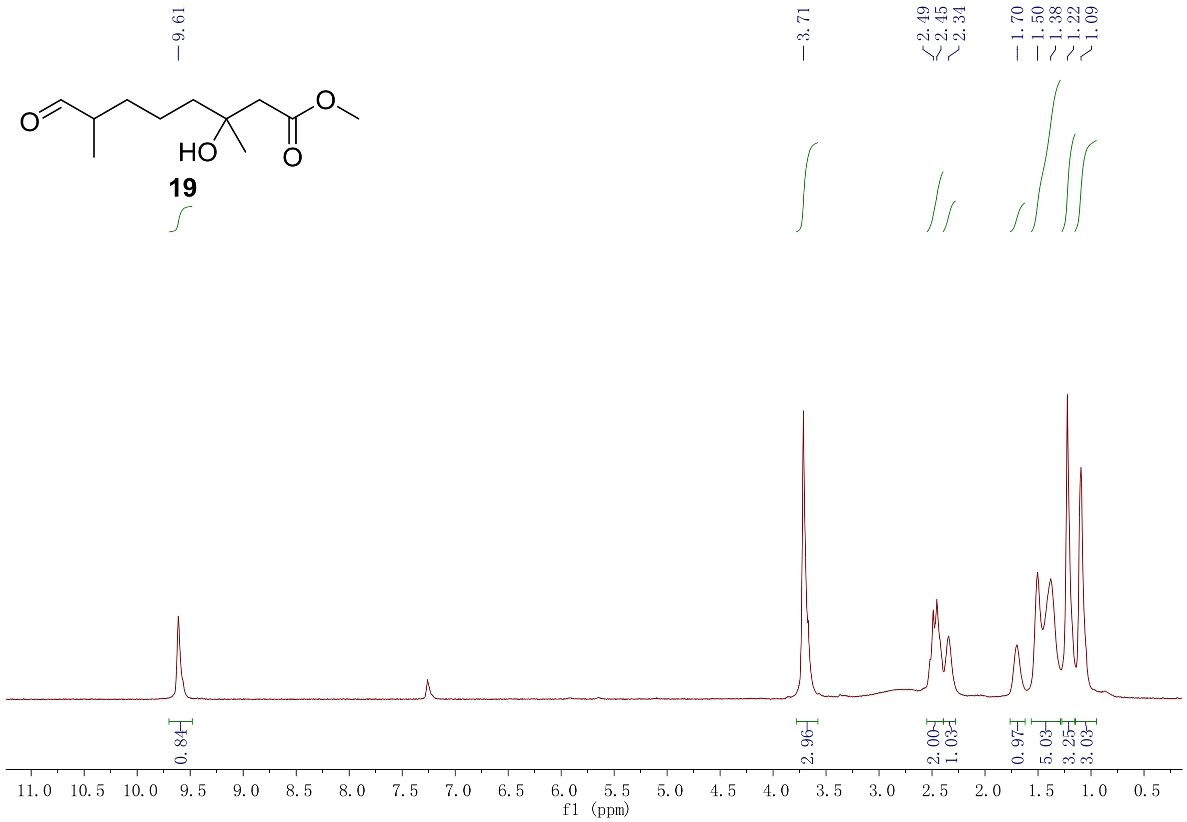

Figure S75. ^{13}C NMR spectrum of compound **19**.

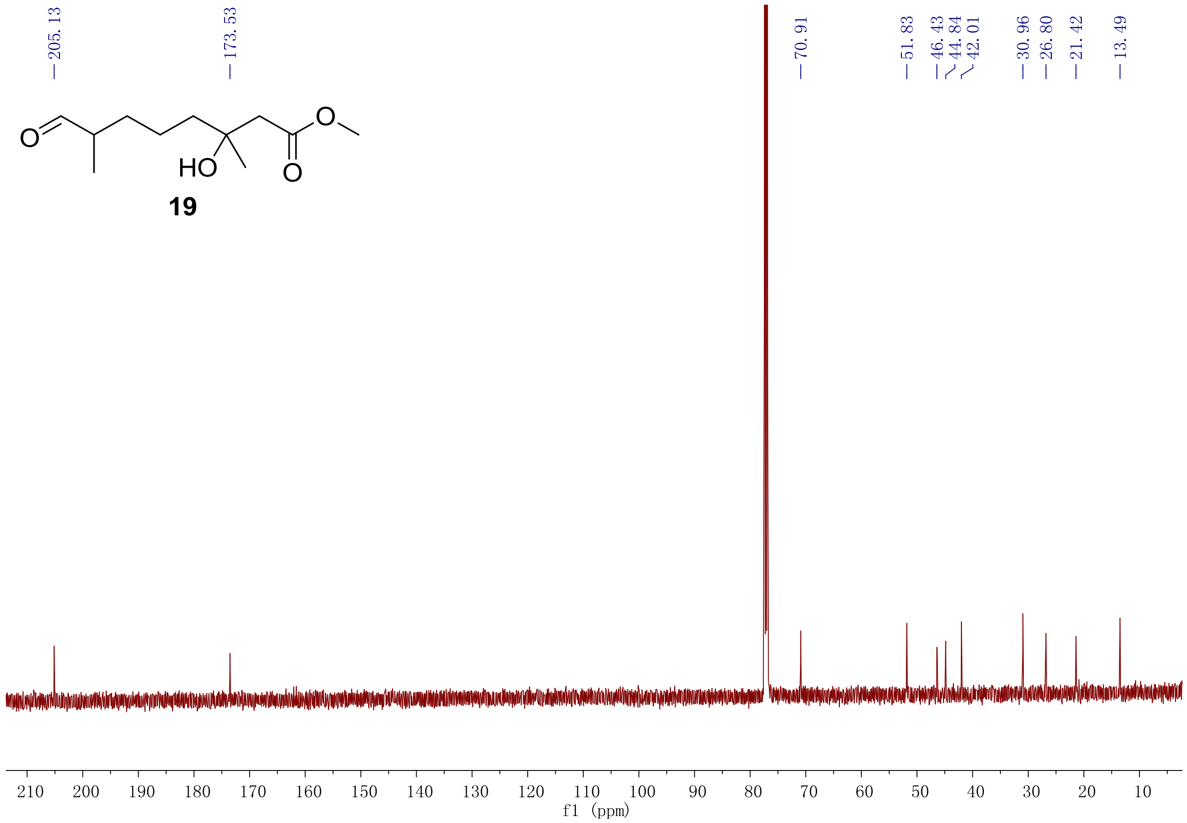

Figure S76. HRMS of compound **19**.

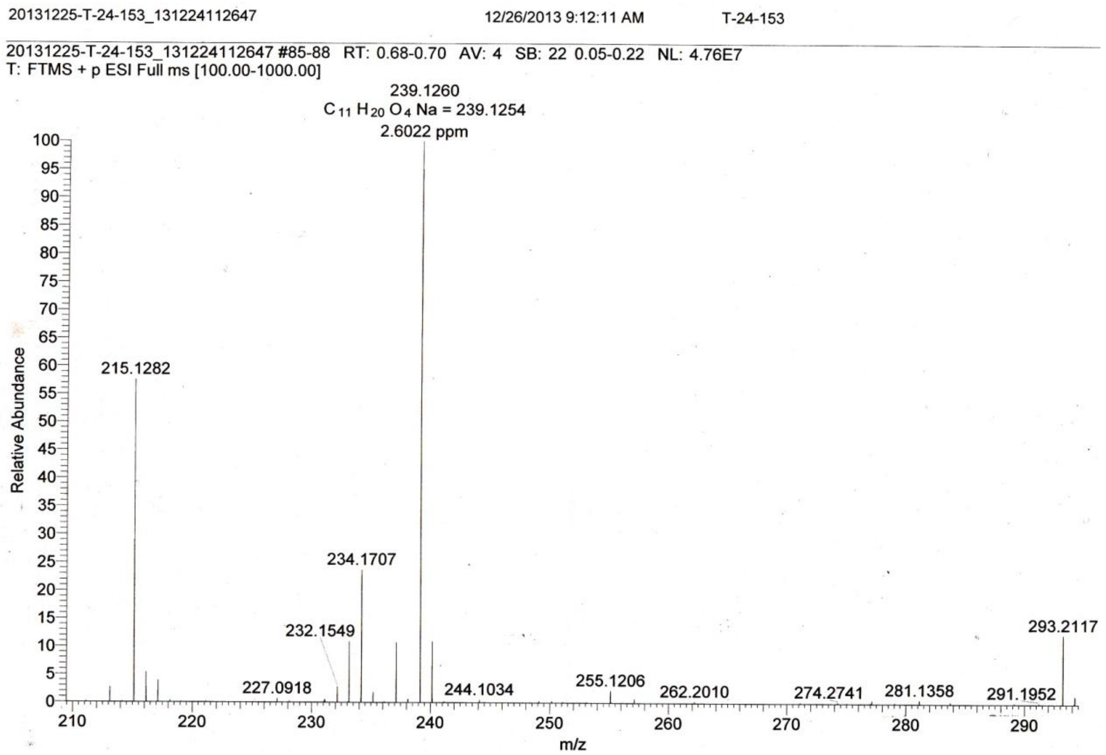

Figure S77. ^{1}H NMR spectrum of compound **20**.

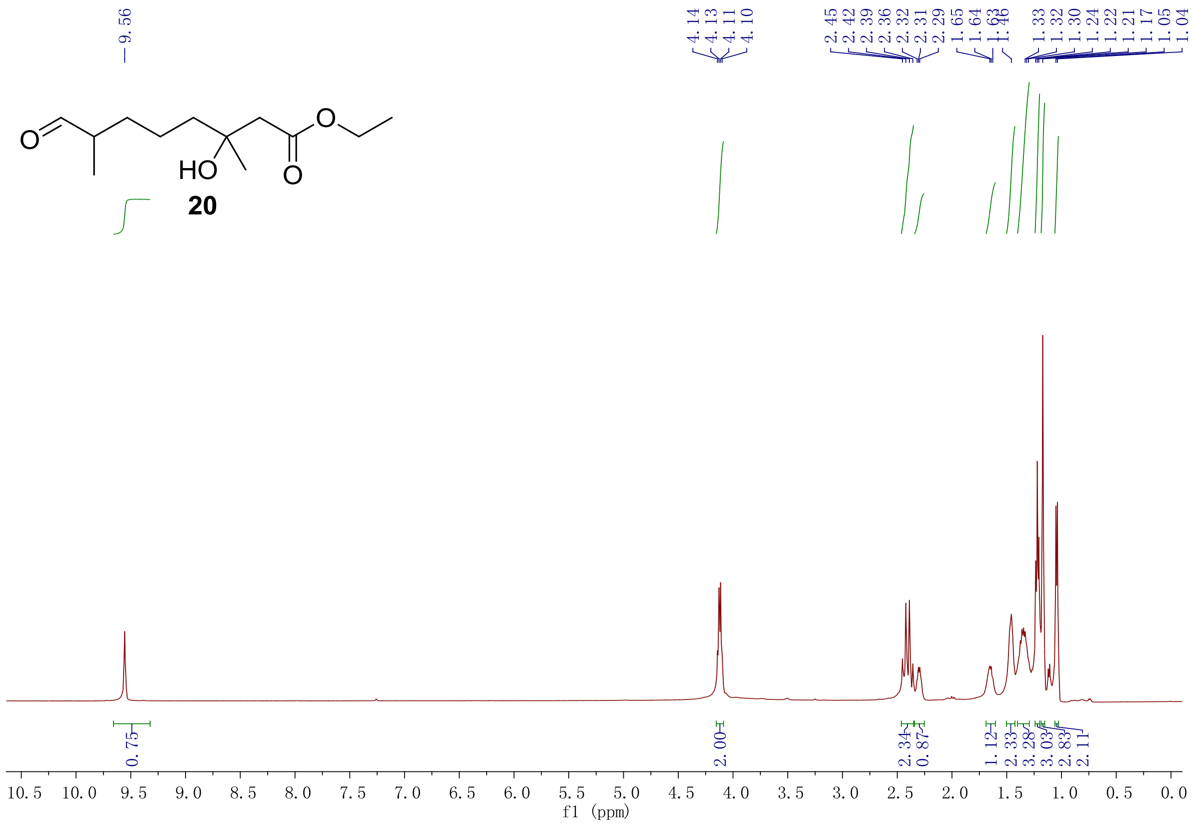

Figure S78. ^{13}C NMR spectrum of compound **20**.

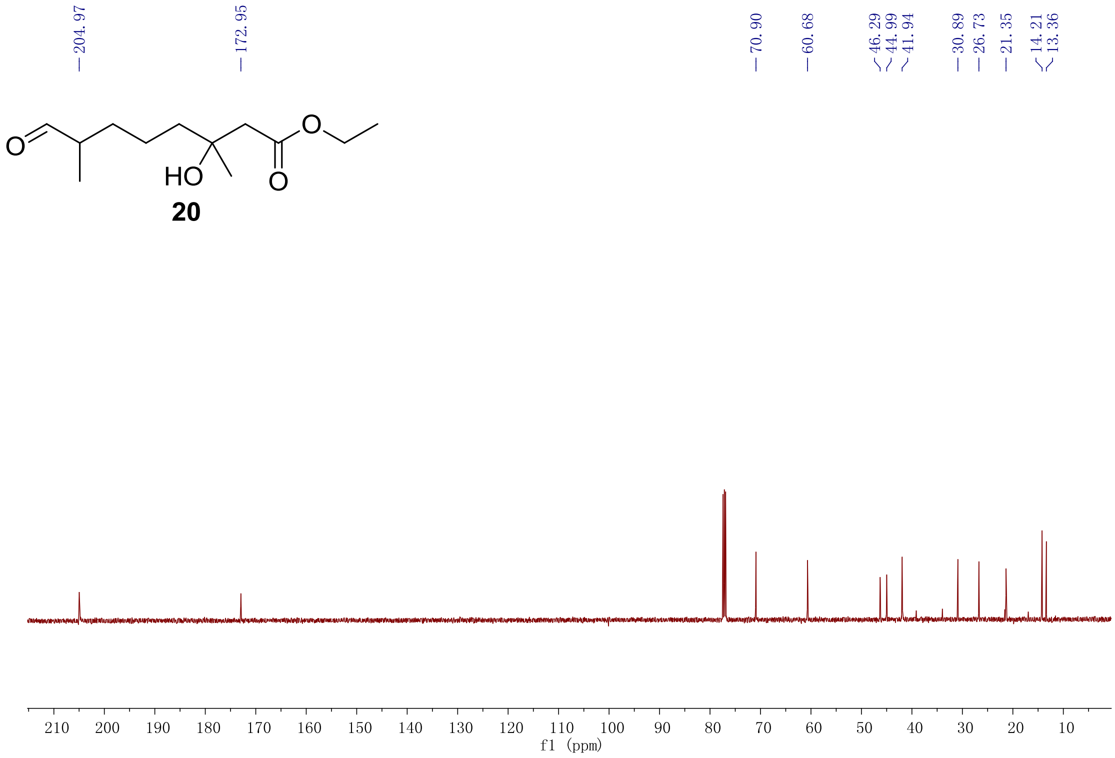

Figure S79. HRMS of compound **20**.

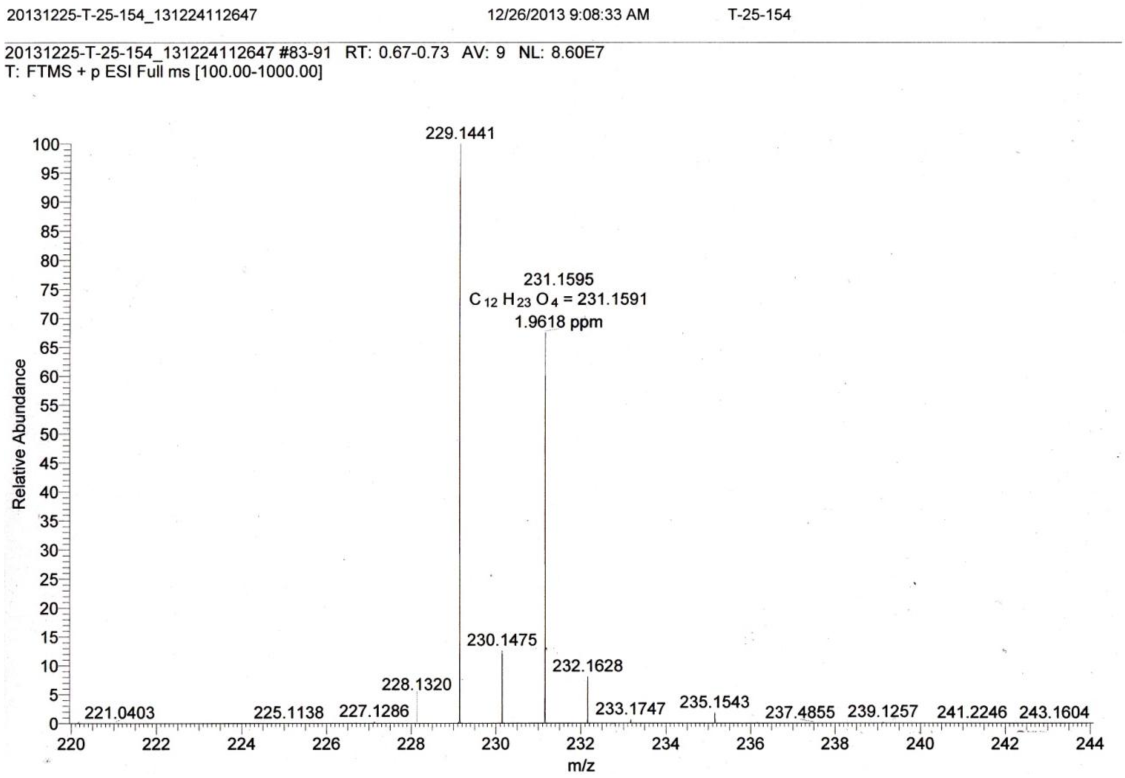

© 2014 by the authors; licensee MDPI, Basel, Switzerland. This article is an open access article distributed under the terms and conditions of the Creative Commons Attribution license (http://creativecommons.org/licenses/by/3.0/).
